# IKAROS is required for the measured response of NOTCH target genes upon external NOTCH signaling

**DOI:** 10.1371/journal.pgen.1009478

**Published:** 2021-03-26

**Authors:** Maud Lemarié, Stefania Bottardi, Lionel Mavoungou, Helen Pak, Eric Milot

**Affiliations:** 1 Maisonneuve-Rosemont Hospital Research Center; CIUSSS de l’est de l’Île de Montréal, Montréal, QC, Canada; 2 Department of Medicine, Université de Montréal, Montréal, Québec, Canada; The Feinstein Institute for Medical Research, UNITED STATES

## Abstract

The tumor suppressor IKAROS binds and represses multiple NOTCH target genes. For their induction upon NOTCH signaling, IKAROS is removed and replaced by NOTCH Intracellular Domain (NICD)-associated proteins. However, IKAROS remains associated to other NOTCH activated genes upon signaling and induction. Whether IKAROS could participate to the induction of this second group of NOTCH activated genes is unknown. We analyzed the combined effect of IKAROS abrogation and NOTCH signaling on the expression of NOTCH activated genes in erythroid cells. In IKAROS-deleted cells, we observed that many of these genes were either overexpressed or no longer responsive to NOTCH signaling. IKAROS is then required for the organization of bivalent chromatin and poised transcription of NOTCH activated genes belonging to either of the aforementioned groups. Furthermore, we show that IKAROS-dependent poised organization of the NOTCH target *Cdkn1a* is also required for its adequate induction upon genotoxic insults. These results highlight the critical role played by IKAROS in establishing bivalent chromatin and transcriptional poised state at target genes for their activation by NOTCH or other stress signals.

## Author summary

NOTCH1 deregulation can favor hematological malignancies. In addition to RBP-Jκ/NICD/MAML1, other regulators are required for the measured activation of NOTCH target genes. IKAROS is a known repressor of many NOTCH targets. Since it can also favor transcriptional activation and control gene expression levels, we questioned whether IKAROS could participate to the activation of specific NOTCH target genes. We are reporting that upon NOTCH induction, the absence of IKAROS impairs the measured activation of two groups of NOTCH target genes: (i) those overexpressed and characterized by an additive effect imposed by the absence of IKAROS and NOTCH induction; and (ii) those ‘desensitized’ and no more activated by NOTCH. At genes of both groups, IKAROS controls the timely recruitment of the chromatin remodelers CHD4 and BRG1. IKAROS then influences the activation of these genes through the organization of chromatin and poised transcription or through transcriptional elongation control. The importance of the IKAROS controlled and measured activation of genes is not limited to NOTCH signaling as it also characterizes *Cdkn1a* expression upon genotoxic stress. Thus, these results provide a new perspective on the importance of IKAROS for the adequate cellular response to stress, whether imposed by NOTCH or genotoxic insults.

## Introduction

NOTCH signaling controls cell proliferation, differentiation, and apoptosis. It is required for hematopoietic stem/progenitor cells (HS/PC) interactions with their environment [1,2]. Disruption or abnormal expression of NOTCH receptors (NOTCH 1 to 4), particularly NOTCH 1, affects hematopoietic cell homeostasis and is common in hematological malignancies including T-cell Acute Lymphocytic Leukemia (T-ALL) [3,4]. NOTCH signaling/induction triggers sequential proteolytic cleavage events that promote nuclear localization of the NOTCH Intracellular Cleaved Domain (NICD). The nuclear NICD forms a ‘ternary complex’ with the transcription factor RBP-Jκ (CBF1) and the coactivator Mastermind-like, thereby favoring the activation of NOTCH target genes. In order to induce transcriptional activation, this ternary complex also recruits co-activators like p300 [5]. Chromatin recruitment of NICD and RBP-Jκ to different target genes can be unstable and may require other transcription factors to be efficiently bound [6]. In mammalian cells different transcription factors including members of the IKZF family such as IKAROS (IKZF1), HELIOS (IKZF2) and AIOLOS (IKZF3) were reported to interact with NICD [7,8]. However, whether these interactions can be functional and stabilize RBP-Jκ/NICD at specific genes is not known.

IKAROS has been identified as an important repressor of NOTCH target genes in lymphoid, myeloid and erythroid cells [7,9–13]. NOTCH target genes downregulated by IKAROS are marked by histone H3K27me3, which is a post-translational modification mainly catalyzed by the Polycomb Repressive Complex 2 (PRC2) methyltransferase enzyme, EZH2. IKAROS, in association with the Nucleosome Remodeling and Deacetylase (NuRD) complex, favors the recruitment of PRC2 to the chromatin [7,13,14] and in erythroid cells, EZH2 is required for IKAROS-dependent repression of *Notch1* and *Hes1* genes [13]. IKAROS can also repress activation of the NOTCH target genes *pTα* and *Hes1* by preventing the recruitment of RBP-Jκ [13,15]. Thus, the repression of NOTCH target genes imposed by IKAROS is likely to be complementary to the canonical mechanism of such repression, which is mediated by chromatin recruitment of RBP-Jκ along with various corepressors [16].

Interestingly, genome wide analyses of transcription factors binding to chromatin suggest that IKAROS can be recruited with NICD to specific NOTCH target genes [7]. However, it remains unclear whether IKAROS participates to the activation of these NOTCH targets upon signaling. Indeed, IKAROS can also act as a transcriptional activator [17–19]. Unlike observed for other transcription factors capable to participate to both transcriptional repression and activation [20], proteomic analyses and target gene characterization have failed to fully clarify how IKAROS can either inhibit or promote transcription, even though this duality may depend on IKAROS ability to associate with several cofactors and complexes on chromatin [21,22]. For instance, the interaction between IKAROS and the NuRD complex, which is critical for chromatin organization of multiple gene regulatory regions in hematopoietic cells [22,23], can lead to either transcriptional repression [24,25] or activation when IKAROS conjointly associates with the NuRD and the Positive-Transcription Elongation Factor b (P-TEFb) complexes [18,23]. P-TEFb must be activated to release CDK9 from the inactive 7SK snRNP complex and promote transcription elongation. Once activated, CDK9 phosphorylates the Pol II C-terminal domain (CTD) at Ser-2, along with the negative elongation factors NELF and DSIF [26] and the conformational changes that follow allow Pol II to enter the productive phase of transcription elongation [27]. IKAROS interacts with CDK9 and PP1 [23,28] and thereby, facilitates their joined recruitment to specific genes [23]. IKAROS mutants with low affinity for PP1 no longer favor proficient transcriptional elongation [23], suggesting that the IKAROS-PP1 interaction promotes P-TEFb activation and, thereby, productive transcriptional elongation. Hence, by associating with different cofactors IKAROS can activate or repress target genes and control the rate of transcriptional elongation [18,23,29].

Homozygous IKAROS knockout mice (Ik^Null^) are characterized by severe lymphopoietic defects [30] and succumb to leukemia or lymphoma with 100% penetrance [31]. IKAROS controls gene expression in HS/PC and several hematopoietic lineage cells [12,29,32–37]. Overexpression of dominant negative IKAROS isoforms frequently characterizes childhood B- and T-ALL [38]. IKAROS is also implicated in erythroid cell gene regulation [13,18,29,39,40] and Ik^Null^ mice develop anemia due to impairment of erythroid cell homeostasis [41,42]. However, it remains to be determined whether the Ik^Null^ effect on these cells is the consequence of the absence of IKAROS regulation imposed on NOTCH target genes.

In this work, we define the crosstalk between IKAROS and NOTCH signaling in erythroid cells. First, we compared the transcriptomes of IKAROS proficient (Ik^WT^) *vs* IKAROS knockout (Ik^Null^) erythroid cells co-cultured with the bone marrow-derived stroma cell line OP9 (control) or OP9-DL1, which ectopically expresses the NOTCH ligand DELTA-like 1 (DL1) [43]. We demonstrate that IKAROS is required for repression of multiple NOTCH target genes and, unexpectedly, is required for activation of others. The molecular characterization of several NOTCH regulated genes indicates that IKAROS and the ATP-dependent chromodomain helicase CHD4 of the NuRD complex are needed to establish bivalent chromatin and poised transcription of NOTCH target genes. Finally, we show that IKAROS is critical for poised transcription and for the controlled expression of the NOTCH target gene *Cdkn1a* (which encodes the P21^WAF1/CIP1^ protein) upon genotoxic insults.

## Results

### Experimental design and gene expression profiles upon IKAROS ablation and NOTCH induction

The main objective of this study was to define whether IKAROS could play a role in activation and repression of NOTCH target genes upon signaling in erythroid cells. To this purpose, embryonic day 14.5 (e14.5) mouse fetal livers were harvested from IKAROS wild type (Ik^WT^) or knockout (Ik^Null^) embryos and co-cultured for 48h with murine bone marrow stromal OP9 or OP9-DL1 cells [43–45]. Since OP9-DL1 cells have been engineered to constitutively express the NOTCH ligand Delta-like 1 (DL1; S1 Fig), they were used to trigger NOTCH signaling. After co-culture, the erythroid cells were isolated by FACS using the erythroid-specific marker Ter119 [46]. RNA was then extracted from Ik^WT^/OP9; Ik^WT^/OP9-DL1; Ik^Null^/OP9; Ik^Null^/OP9-DL1 Ter119^+^ cells and RNA-sequencing (RNA-seq) was performed in biological triplicate. The RNA-seq results were normalized with the DESeq package [47]. Briefly, the read count was normalized by size factor that was calculated by median ratio of gene counts relative to geometric mean per gene. If not otherwise stated, we retained differentially expressed genes displaying log2 fold change ≥ 0.8 or ≤ -0.8 and a False Discovery rate (FDR) < 0.05. A pilot assay revealed that the profile of the Ter119^+^ cells comprised within the P3 and P4 populations (S2 Fig and [13]) does not show variation, regardless of IKAROS expression or co-culture with OP9 or OP9-DL1 cells. Thus, like previously reported [48,49], the absence of IKAROS or induction of the NOTCH pathway only have minor effect on the fetal liver Ter119^+^ erythroid cells population and hence, will allow us to perform comparative analyses.

**Fig 1:**
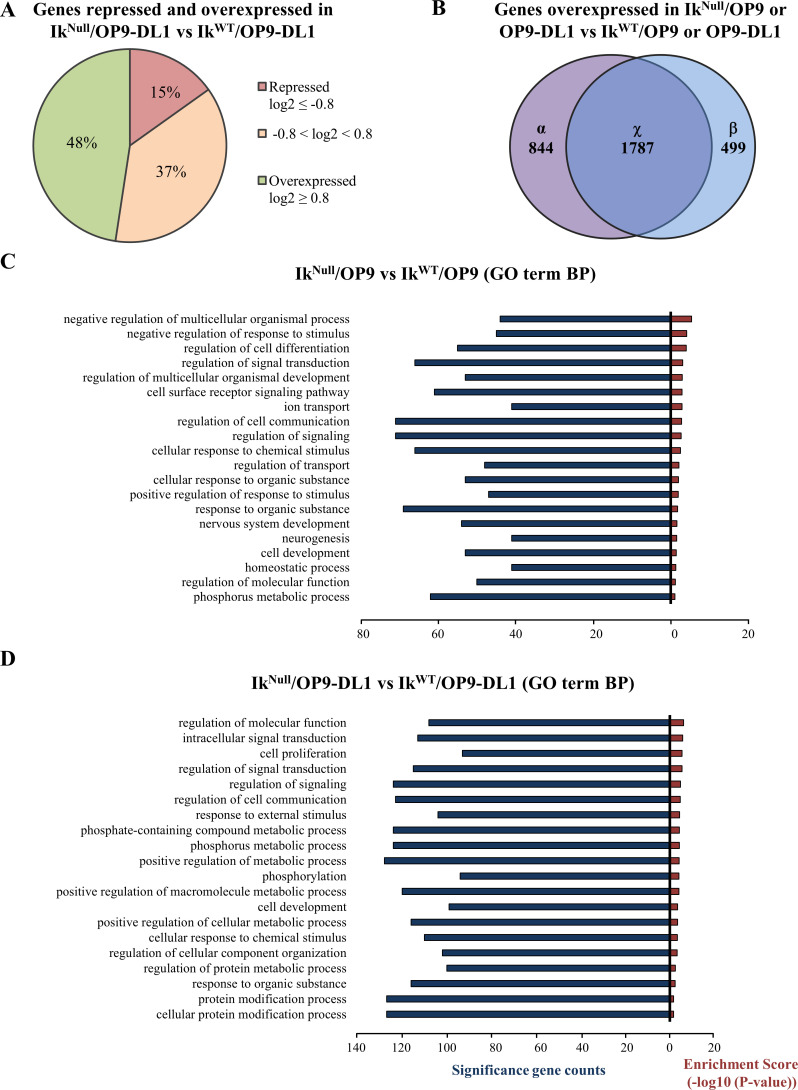
RNA-sequencing analysis of Ik^WT^ and Ik^Null^ Ter119^+^ cells upon IKAROS ablation and NOTCH pathway activation. (A) Circular diagram depicting repressed (red) or overexpressed (green) genes in Ik^Null^/OP9-DL1 compared to Ik^WT^/OP9-DL1 cells (log2 ≥ 0.8 or ≤ -0.8; FDR < 0.05; *p* < 0.05). (B) Venn diagram summarizing the overlap between overexpressed genes (log2 ≥ 0.8) in Ik^Null^ *vs* Ik^WT^ Ter119^+^ cells co-cultured with OP9-DL1 (α; left circle) or OP9 (β; right circle) cells; the number of genes called by both conditions is indicated by the overlap between the two circles (χ; middle circle). (C, D) DAVID functional annotation clustering analyses according to gene biological process; comparative RNA expression of Ik^Null^/OP9 *vs* Ik^WT^/OP9 (panel C) or Ik^Null^/OP9-DL1 *vs* Ik^WT^/OP9-DL1 (panel D) cells; bar graphs indicate the number of genes overexpressed in Ik^Null^ cells (log2 ≥ 0.8; FDR < 0.05; *p* < 0.05).

Although IKAROS is described as a repressor of NOTCH target genes [7,10,11,13,50], our results revealed two opposing patterns of gene regulation imposed by the absence of IKAROS in Ik^Null^ cells upon the induction of the NOTCH pathway, *i*.*e*., overexpression and downregulation. This outcome suggests that upon NOTCH induction, IKAROS can either repress or activate NOTCH target genes (Fig 1A).

We first analyzed genes overexpressed upon the loss of IKAROS. Comparative analysis of RNA-seq results of Ik^Null^ and Ik^WT^ cells co-cultured with either OP9 or OP9-DL1 cells revealed that 3130 genes were overexpressed in Ik^Null^ Ter119^+^ cells (Figs 1B and S3 and S1 File). As expected, and confirming the robustness of our experimental model, the expression of multiple NOTCH target genes increased upon DL1 mediated activation, in Ik^WT^/OP9-DL1 *vs* Ik^WT^/OP9 (S3 Table). These differentially expressed genes were analyzed with the bioinformatic software clustering DAVID [51,52] and regrouped according to cellular function (S4 Table). Analysis of the transcriptome in Ik^Null^/OP9 *vs* Ik^WT^/OP9 indicated that 2286 genes were overexpressed in Ik^Null^/OP9 (χ+β; Figs 1B and S3 and S1 File), whereas analysis of Ik^Null^/OP9-DL1 *vs* Ik^WT^/OP9-DL1 revealed that 2631 genes were overexpressed in Ik^Null^/OP9-DL1 (χ+α; Figs 1B and S3 and S1 File). The majority of the overexpressed genes was common to both comparative analyses (1787 genes: χ; Figs 1B and S3 and S1 File), suggesting that their expression is enhanced by the absence of IKAROS independently of extracellular NOTCH signaling. Functional annotation analysis of these genes revealed that regardless of NOTCH induction, IKAROS deletion has a dominant effect on the erythroid transcriptome and could affect the formation and functionality of erythroid cells since genes associated with cellular differentiation, homeostasis, signaling or iron transport are affected in Ik^Null^ cells (Fig 1C and 1D). The comparative analysis of gene expression profiles indicates that 499 genes are specifically overexpressed in Ik^Null^/OP9 *vs* Ik^WT^/OP9 and 844 genes are uniquely overexpressed in Ik^Null^/OP9-DL1 *vs* Ik^WT^/OP9-DL1 (respectively, β and α sets; Fig 1B and S1 File). Most of the 499 genes specifically overexpressed in Ik^Null^/OP9 cells can be functionally annotated as cell proliferation, homeostasis, cell-to-cell communication, signaling and metabolism (S5 Table). Alternatively, Ik^Null^/OP9-DL1 overexpressed genes are among others, annotated as regulators of gene transcription, inflammation, lymphoid cell activity and cell death (S6 Table). Thus, it can be assumed that the effect of IKAROS depletion and NOTCH induction further affects the transcriptome and, most likely, the function of erythroid cells.

**Fig 2:**
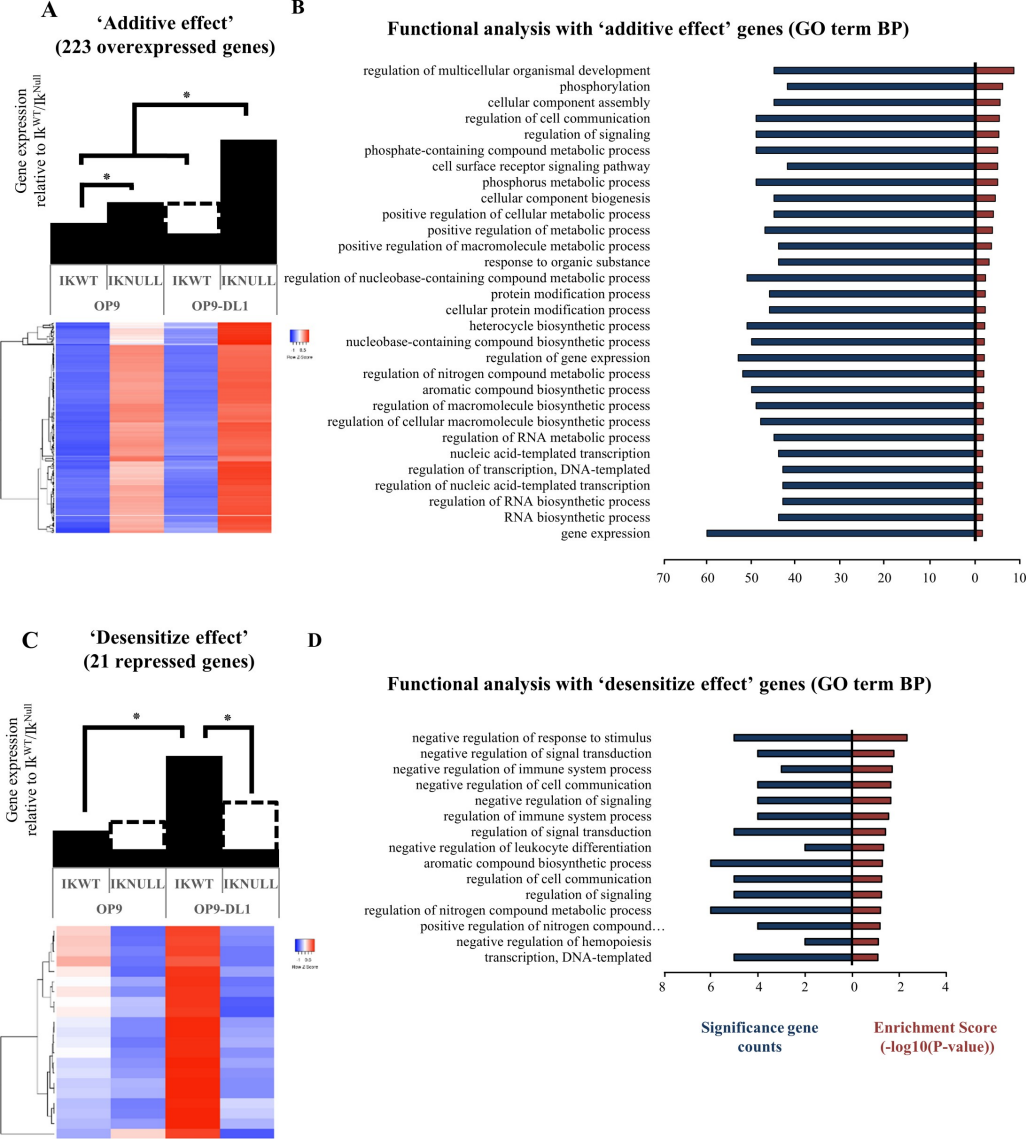
IKAROS deletion promotes deregulation of several genes related to different cellular functions upon NOTCH induction. (A, C) Model bar graphs and heatmaps depicting the expression pattern of 223 overexpressed (panel A) or 21 repressed (panel C) genes upon IKAROS ablation and NOTCH induction (Ik^Null^ *vs* Ik^WT^ on OP9 cells relative to Ik^Null^ *vs* Ik^WT^ on OP9-DL1 cells). Colors on the heatmaps (depicted in the color key) represent the relative expression levels of individual genes in the four conditions used for this study (as indicated above the heatmaps); unsupervised hierarchical clustering (Pearson correlation, average linkage) over the genes included in each group, is depicted on the left side of the heatmaps. (B, D) DAVID functional annotation clustering analyses of genes identified and corresponding to panel A or C according to gene biological process; bar graphs indicate the number of genes differentially expressed (log2 ≥ 0.8; FDR < 0.05; *p* < 0.05).

The role of IKAROS as a repressor of NOTCH target genes has been well described [7,9–11,13]. Our RNA-seq analysis highlighted this repressive effect and, interestingly, allowed the identification of 223 genes whose overexpression requires the conjoint removal of IKAROS and induction of the NOTCH pathway (overexpression in Ik^Null^/OP9-DL1 above all other conditions; Fig 2A and S2 File). We named this phenomenon the ‘additive effect’. Overexpression of two randomly selected genes (*Il6* and *Zfp446*) in Ik^Null^ e14.5 fetal liver erythroid cells was confirmed by qRT-PCR (S4A Fig). The biological processes affected by the ‘additive effect’ include signaling, transcription control, metabolism, cellular growth, and proliferation (Fig 2B). Importantly, several genes characterized by the ‘additive effect’ encode tumor suppressors or oncogenes (S7 Table). Their abnormal expression can perturb hematopoietic cell homeostasis, contribute to malignant transformation, modify cell interactions with the niche, and/or affect cellular capacity to provide an adaptive immune response.

RNA-seq analysis also suggests that IKAROS deletion can transcriptionally desensitize NOTCH target genes to NOTCH induction (repressed genes; Fig 1A). Indeed, our data indicate that among genes activated by NOTCH (Ik^WT^/OP9-DL1 *vs* Ik^WT^/OP9), 21 are characterized by a significant decrease of their expression levels when IKAROS is missing (Ik^Null^/OP9-DL1 *vs* Ik^WT^/OP9-DL1), hence revealing that IKAROS can also contribute to NOTCH-associated gene transcriptional activation (Fig 2C). We named this phenomenon the ‘desensitize effect’. Functional annotation analysis of these genes was performed and indicated that 3 of the genes affected by IKAROS deletion in the context of NOTCH induction are involved in response to stimulus (Fig 2D). Oncogenes and tumor suppressor genes are also among the 21 genes requiring IKAROS to be properly stimulated upon NOTCH induction (S8 Table). In summary, these results suggest that IKAROS is required to prime and favor the physiological activation of different NOTCH target genes upon NOTCH induction.

### IKAROS is required to attain a physiological response upon NOTCH induction

To elucidate the mechanism(s) used by IKAROS to either repress or prime NOTCH target genes, we studied the regulation of representative genes from either ‘groups’ *i*.*e*., those characterized by the ‘additive effect’ or the ‘desensitize effect’.

**Fig 3:**
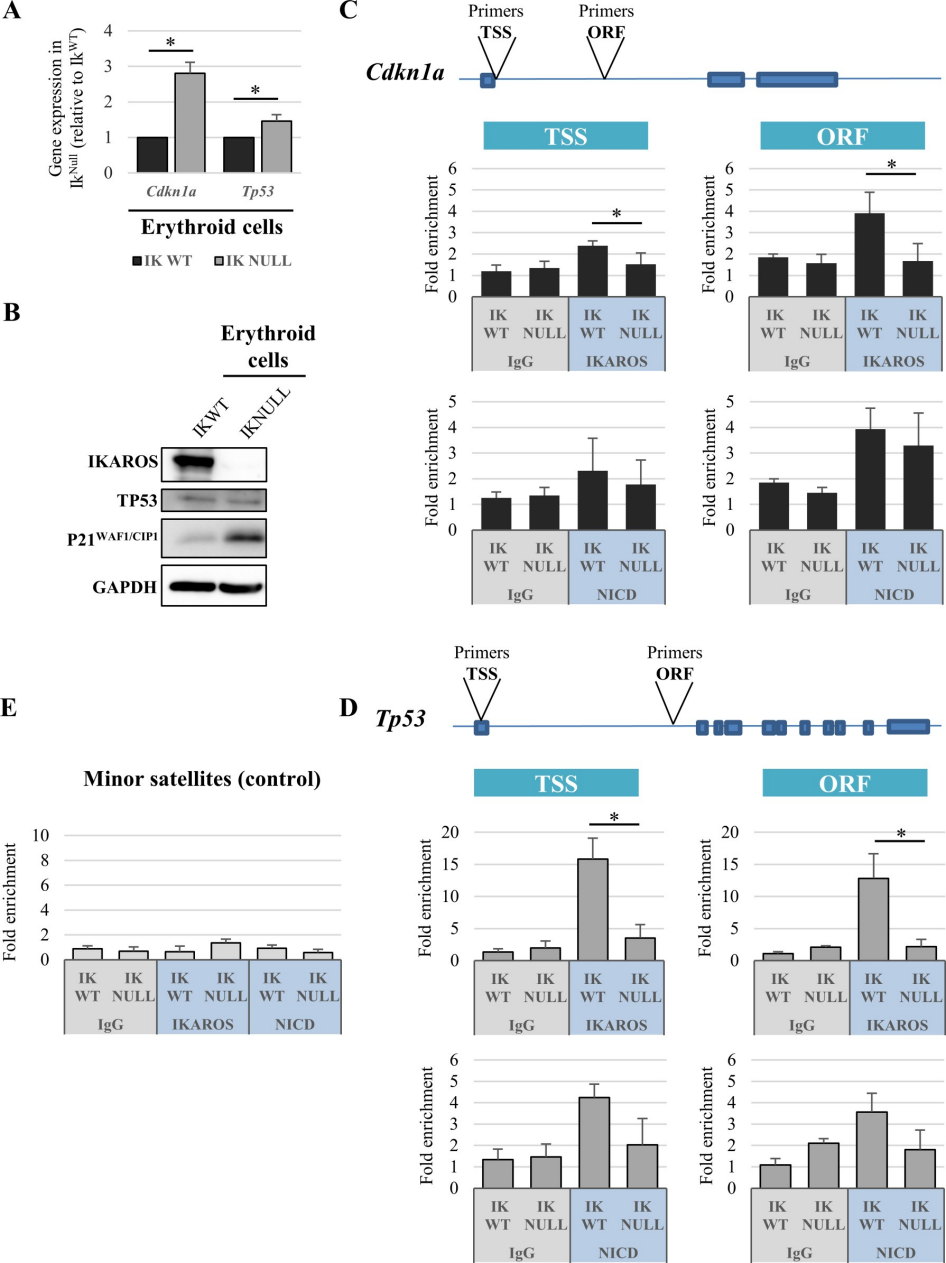
Characterization of *Cdkn1a* and *Tp53* genes in Ik^WT^ and Ik^Null^ erythroid cells. (A) Gene expression; the relative expression levels of *Cdkn1a* and *Tp53* genes in Ik^WT^ or Ik^Null^ erythroid cells were measured by qRT-PCR, calculated according to the Pfaffl equation using *Hprt* as internal control and normalized to Ik^WT^; *y* axis: relative RNA enrichment levels; ratios are represented by bars and are plotted as the mean ± Standard Deviation (SD) of the measurements; data shown are the results of three independent experiments; *: *p* ≤ 0.05 by Student’s *t*-test. (B) Western blot analysis of IKAROS, TP53, P21^WAF1/CIP1^ and GAPDH (used as loading control) expression in Ik^WT^ or Ik^Null^ erythroid cell lysates. (C, D, E) Chromatin immunoprecipitation assays in Ik^WT^ or Ik^Null^ Ter119^+^ cells with IKAROS, NICD antibodies (blue) or isotype-matched IgG (grey); immunoprecipitated and unbound (input) chromatin samples were used as templates in qPCR analysis with primers specific for *Cdkn1a* (C), *Tp53* (D) or the minor satellite repeat control region (E); *y-axis*: fold enrichment levels calculated according to the Pfaffl equation using the *Thp1* promoter region as internal control, are represented by bars and plotted as the mean ± SD of the measurements; a value of 1 indicates no enrichment; data shown are the results of three independent experiments; TSS: transcription start site; ORF: open reading frame; NICD: NOTCH intracellular domain; *: *p* ≤ 0.05 by Student’s *t*-test.

Among the 223 genes repressed by IKAROS and overexpressed upon joint NOTCH induction and IKAROS ablation (additive effect: Fig 2A and S9 Table), we chose *Cdkn1a* and *Tp53*, which encode respectively P21^WAF1/CIP1^ and TP53. TP53 and its downstream effector P21^WAF1/CIP1^ are critical factors in cancer biology (S7 Table). The expression levels of *Cdkn1a* and *Tp53* as well as P21^WAF1/CIP1^ but not TP53, increase in Ik^Null^ erythroid cells (Fig 3A and 3B). Unchanged TP53 level in Ik^Null^ cells most likely relates to the fact that TP53 is highly regulated by post-translational mechanisms [53,54] that can mask the contribution of IKAROS.

*Cdkn1a* and *Tp53* genes were previously identified as NOTCH target genes [55–57]. To determine whether IKAROS can directly regulate *Cdkn1a* and *Tp53* genes, we performed chromatin immunoprecipitation (ChIP) with antibodies directed against IKAROS or NICD. IKAROS and NICD were detected at the Transcription Start Site (TSS) region and Open Reading Frame (ORF) of both genes (Fig 3C and 3E) in Ik^WT^ erythroid cells, thus indicating their recruitment to *Cdkn1a* and *Tp53* NOTCH target genes in these cells. Since NICD binding to chromatin in freshly isolated erythroid cells was unexpected, we investigated NICD recruitment levels upon NOTCH induction in Ik^Null^/OP9-DL1 cells (S4B Fig), whereby the expression of *Cdkn1a* and *Tp53* is optimal. The results of ChIP performed in erythroid cells co-cultured with OP9-DL1 indicate that the recruitment of NICD to the chromatin of target genes dramatically increases, when compared to the 1-to-4-fold enrichment level observed in fetal liver erythroid cells (Figs S4B and 3C and 3D). Therefore, the weak NICD recruitment observed in fetal liver (Fig 3C and 3D) and in Ik^Null^/OP9 (S4B Fig), might not be biologically significant and might be related to background detection associated with this specific antibody under our experimental conditions (*i*.*e*., double HCHO-EGS fixation; see Materials and Methods). Altogether, these results are reminiscent of those reported in thymocytes where IKAROS and RBP-Jκ can be recruited to the same regulatory regions of NOTCH activated genes [7]. Whether IKAROS can be co-recruited with RBP-Jκ and influence NICD recruitment to *Cdkn1a* and *Tp53* in erythroid cells remains to be determined.

**Fig 4:**
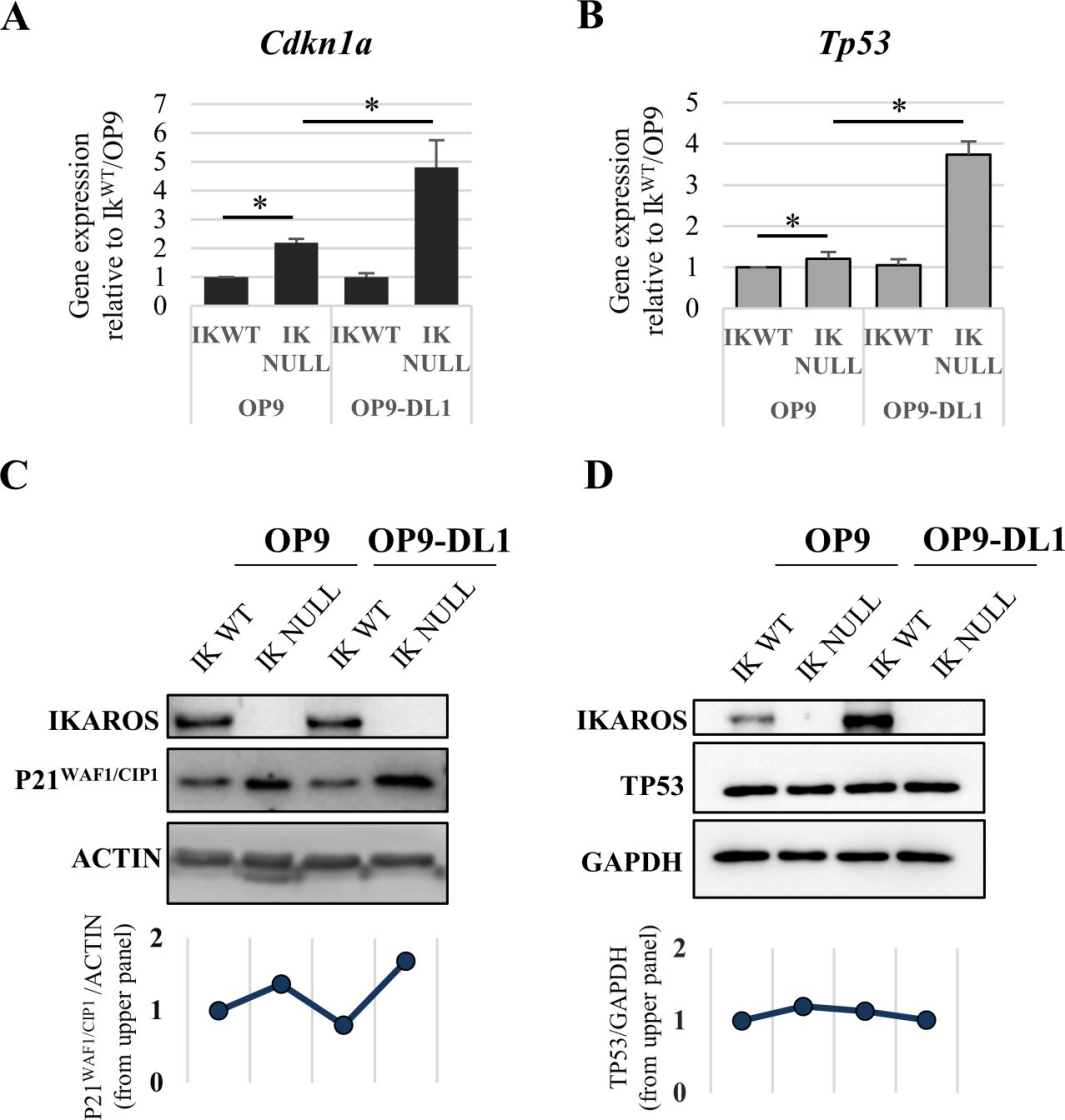
*Cdkn1a* and *Tp53* gene and protein expression in Ik^WT^ and Ik^Null^ Ter119^+^ cells upon NOTCH induction. (A, B) Gene expression; the relative expression levels of *Cdkn1a* and *Tp53* genes in Ik^WT^ and Ik^Null^ Ter119^+^ cells before (OP9 co-culture) or after (OP9-DL1 co-culture) NOTCH induction, were measured by qRT-PCR, calculated according to the Pfaffl equation using *Hprt* as internal control and normalized to Ik^WT^/OP9; *y* axis: relative RNA enrichment levels, ratios are represented by bars and are plotted as the mean ± Standard Deviation (SD) of the measurements; data shown are the results of five independent experiments; *: *p* ≤ 0.05 by Student’s *t*-test. (C, D) Western blot analysis of IKAROS (65 KDa), P21^WAF1/CIP1^ (21 KDa), TP53 (53 KDa) and ACTIN (42 KDa) or GAPDH (38 KDa) [used as loading controls] expression in Ik^WT^ and Ik^Null^ Ter119^+^ cells before (OP9 co-culture) or after (OP9-DL1 co-culture) NOTCH induction; dot graphs: relative quantification of P21^WAF1/CIP1^ (panel C; also refer to S5 Fig) or TP53 (panel D) proteins relative to ACTIN or GAPDH, respectively.

We further analyzed the additive effect of IKAROS abrogation and NOTCH induction by means of the OP9/OP9-DL1 co-culture system. In accordance with RNA-seq results, qRT-PCR analysis indicated that the expression levels of *Cdkn1a* and *Tp53* significantly increase in Ik^Null^/OP9-DL1 cells (Fig 4A and 4B), suggesting that the interplay between IKAROS and NOTCH is biologically relevant and particularly important to control the expression of P21^WAF1/CIP1^ (Figs 4C and 4D and S5), which is a critical regulator of cellular stress response [58,59].

**Fig 5:**
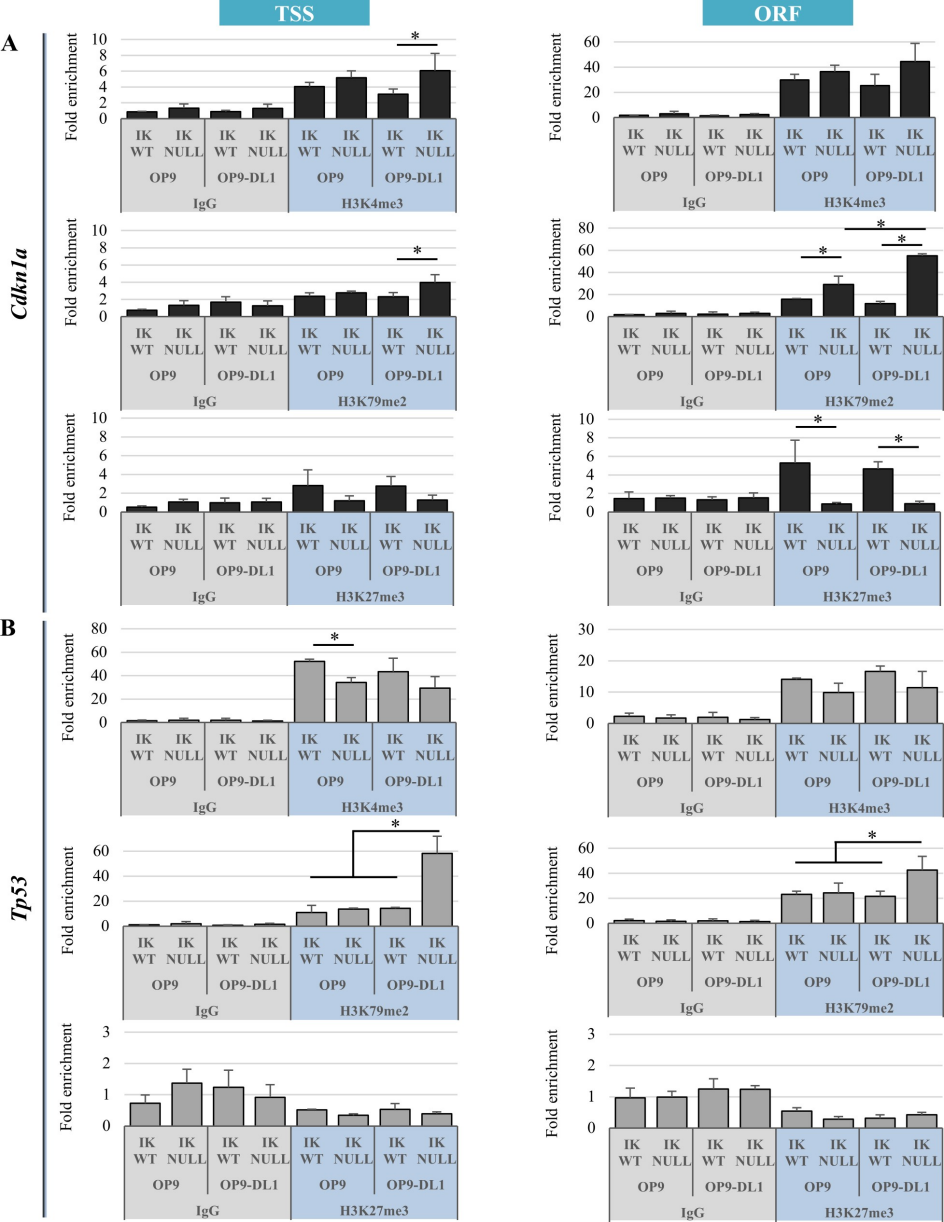
Histone post-translational modifications at *Cdkn1a* and *Tp53* genes in Ik^WT^ and Ik^Null^ Ter119^+^ cells upon NOTCH induction. (A, B) ChIP assays with H3K4me3, H3K79me2, H3K27me3 antibodies (blue) or isotype-matched IgG (grey) were conducted in Ik^WT^ or Ik^Null^ Ter119^+^ cells co-cultured with OP9 or OP9-DL1 cells; immunoprecipitated and unbound (input) chromatin samples were used as templates in qPCR analysis with primers specific for *Cdkn1a* (A) or *Tp53* (B) TSS region and ORF; qPCR analysis of the minor satellite repeat control region is presented in S6A Fig; *y-axis*: fold enrichment levels calculated according to the Pfaffl equation using *Thp1* promoter region as internal control, are represented by bars and plotted as the mean ± Standard Deviation of the measurements; a value of 1 indicates no enrichment; data shown are the results of three independent experiments; *: *p* ≤ 0.05 by Student’s *t*-test.

IKAROS can influence transcriptional activation and elongation at selected target genes [17,36,60]. Hence, we employed ChIP assays to analyze the role of IKAROS for transcription control of *Cdkn1a* and *Tp53* genes, by studying the distribution of histone post-translational modifications. IKAROS synergizes with the PRC2 enzyme EZH2 [13,14] and with the transcription elongation licensing factor P-TEFb [18,61]. Thus, H3K27me3 (deposited by EZH2) as well as H3K4me3 and H3K79me2, which are typical marks of productive elongation [62], were studied to define the role of IKAROS as transcriptional repressor and/or activator. In Ik^WT^/OP9 and Ik^WT^/OP9-DL1 cells, *Cdkn1a* TSS and ORF are marked with H3K4me3, H3K79me2 and H3K27me3 (Figs 5A and S6A). This chromatin state closely resembles bivalent chromatin of hematopoietic stem/progenitor cells harboring H3K4me3 and H3K27me3 [63] and marking genes poised or primed for rapid transcriptional activation [64]. *Cdkn1a* overexpression (Fig 4A), higher H3K79me2 and lower H3K27me3 levels at *Cdkn1a* regulatory regions in Ik^Null^/OP9 and Ik^Null^/OP9-DL1 cells, suggest that IKAROS is required for the bivalent organization of the chromatin and the poised state of this gene (Fig 5A). To test whether IKAROS could be required for the establishment of a bivalent chromatin characterized by the simultaneous co-occupancy of H3K4me3 and H3K27me3 to the *Cdkn1a* TSS, sequential ChIP (re-ChIP) [65] assays were carried out. The results obtained indicate that these histone post-translational modifications are simultaneously enriched at *Cdkn1a* TSS (S6B Fig), hence suggesting that IKAROS favors bivalent chromatin organization of the *Cdkn1a* gene prior to its induction by NOTCH. Finally, the peak of H3K79me2 levels at *Cdkn1a* TSS and ORF in Ik^Null^/OP9-DL1 cells (Fig 5A), strengthened the additive effect imposed by IKAROS ablation and NOTCH induction.

In Ik^WT^ cells, chromatin at *Tp53* contains H3K4me3 and H3K79me2 but not H3K27me3 marks (Fig 5B). The latter suggests that IKAROS does not contribute to PRC2 recruitment, bivalent chromatin organization (S6B Fig) or repression of this gene. Indeed, *Tp53* is transcriptionally active in erythroid cells and we have shown that conjoint IKAROS deletion and NOTCH induction lead to *Tp53* overexpression (Fig 4B). Thus, IKAROS may not trigger *Tp53* repression, but the increase of H3K79 methylation and the ‘additive effect’ observed in Ik^Null^/OP9-DL1 suggest that, by controlling H3K79me2 levels, IKAROS prevents the NOTCH-dependent induction of *Tp53* transcriptional overexpression, which is likely linked to a variation in the control of transcriptional elongation.

To further investigate the importance of H3K79me2 deposition for occurrence of the additive effect, the cells were treated with EPZ004777, a known inhibitor of DOT1L (inhDOT1L), which is the unique methyltransferase capable of H3K79 methylation in mammalian cells [62]. The erythroid model cell line G1E-ER4 was used to define the lowest concentration of EPZ004777 (3μM) capable to decrease DOT1L activity and hence, H3K79me2 deposition (S7A Fig). G1E-ER4 are ES-derived pro-erythroblast GATA-1^null^ cells expressing an inducible GATA-1-estrogen receptor (GATA-1-ER) fusion protein, which accumulates in the nucleus upon Tamoxifen induction and allows differentiation into erythroid cells [66–68]. Treatment with 3μM EPZ004777 severely reduced the cellular levels (S7B Fig) as well as the deposition of H3K79me2 to *Cdkn1a* and *Tp53* TSS region (S7C Fig) in erythroid Ik^WT^ and Ik^Null^ Ter119^+^ cells co-cultured with OP9-DL1. The inhibition of DOT1L disrupts the additive effect imposed by IKAROS deletion and NOTCH induction on *Tp53* but does not significantly change the additive effect detected at *Cdkn1a* (S7D Fig *vs* Fig 4A). This suggests that DOT1L activity is requested for *Tp53* overexpression imposed by the additive effect of IKAROS deletion and NOTCH induction. Currently, it is unclear why DOT1L inhibition and H3K79 methylation reduction do not correlate with a lower expression levels of *Cdkn1a* and *Tp53* in the absence of NOTCH induction. However, it is known that a direct link between H3K79 methylation and expression levels is not common to all genes [69] and, herein, it might be influenced by specific regulatory mechanisms occurring when IKAROS-CHD4 *vs* NICD or BRG1 complexes are alternatively recruited to these genes.

### Disruption of IKAROS-dependent recruitment of the CHD4/NuRD complex leads to the abnormal expression of NOTCH target genes

The implication of chromatin remodeling activities for the ‘additive’ and ‘desensitize’ effects of IKAROS depletion and NOTCH induction was also explored. IKAROS can interact with NuRD and BAF subunits in lymphoid and erythroid cells thereby, controlling gene expression [19,23,70–72]. Interestingly, NICD was also reported to interact with NuRD and PBAF [8]. Therefore, we assessed the recruitment of the remodeling ATPases CHD4 and BRG1, respectively included in NuRD and P/BAF (BAF or PBAF) complex, to genes characterized by the ‘additive’ (*Cdkn1a* and *Tp53*) or ‘desensitize’ (*Prdm16* and *Nrarp*, as described below; S2 File) effect in erythroid cells.

**Fig 6:**
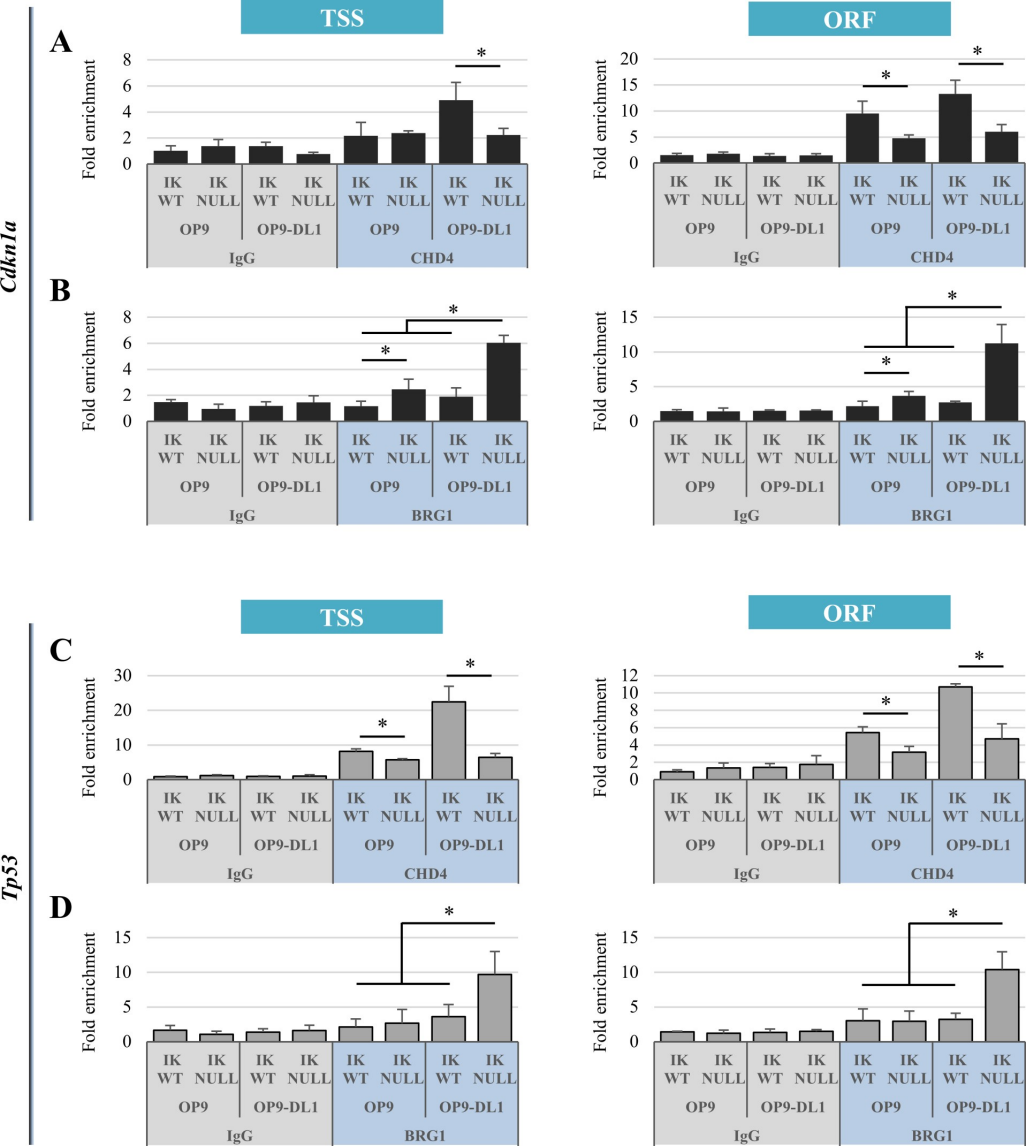
BRG1 and CHD4 recruitment to *Cdkn1a* and *Tp53* genes in Ik^WT^ and Ik^Null^ Ter119^+^ cells upon NOTCH induction. (A-D) ChIP assays with CHD4, BRG1 antibodies (blue) or isotype-matched IgG (grey) were conducted in Ik^WT^ or Ik^Null^ Ter119^+^ cells co-cultured with OP9 or OP9-DL1 cells; immunoprecipitated and unbound (input) chromatin samples were used as templates in qPCR analysis with primers specific for *Cdkn1a* (A, B) or *Tp53* (C, D) TSS and ORF; *y-axis*: fold enrichment levels calculated according to the Pfaffl equation using *Thp1* promoter region as internal control, are represented by bars and plotted as the mean ± Standard Deviation of the measurements; a value of 1 indicates no enrichment; data shown are the results of three independent experiments; *: *p* ≤ 0.05 by Student’s *t*-test.

As expected, CHD4 of the NuRD complex, was recruited to *Cdkn1a* and *Tp53* TSS and ORF in Ik^WT^/OP9 and Ik^WT^/OP9-DL1 cells. CHD4 recruitment to these genes was IKAROS-dependent and higher in Ik^WT^/OP9-DL1 than Ik^WT^/OP9 cells (Fig 6A and 6C), suggesting that it is facilitated by conjoint NICD binding to these genes (Fig 3C–3E). Conversely, the BAF component BRG1 was more efficiently recruited to *Cdkn1a* and *Tp53* regions in Ik^Null^/OP9-DL1 cells (Fig 6B and 6D). Thus, the ‘additive effect’ observed upon IKAROS ablation and NOTCH induction might be due to lower CHD4/NuRD and higher BRG1-P/BAF recruitment to chromatin.

**Fig 7:**
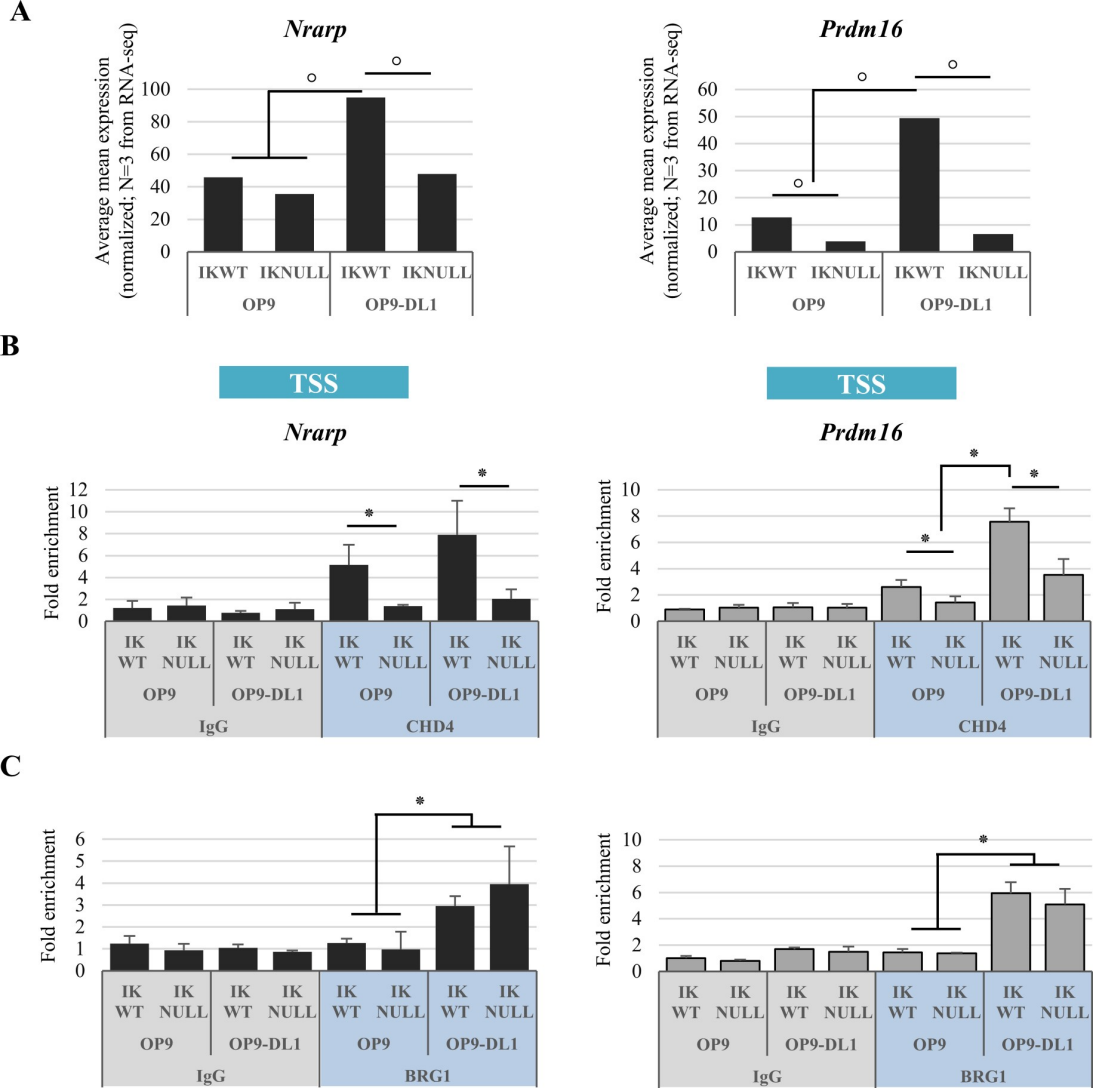
Characterization of *Nrarp* and *Prdm16* genes in Ik^WT^ and Ik^Null^ Ter119^+^ cells upon NOTCH induction. (A) Gene expression; the relative expression levels of *Nrarp* and *Prdm16* genes in Ik^WT^ and Ik^Null^ mouse fetal liver Ter119^+^ cells before (OP9 co-culture) or after (OP9-DL1 co-culture) NOTCH induction, as defined by RNA-sequencing analysis; *y*-axis: fold enrichment levels are represented by bars (average mean expression normalized; from the RNA-seq results; N = 3); (B, C) ChIP assays with CHD4, BRG1 antibodies (blue) or isotype-matched IgG (grey) were conducted in Ik^WT^ or Ik^Null^ Ter119^+^ cells co-cultured with OP9 or OP9-DL1 cells; immunoprecipitated and unbound (input) chromatin samples were used as templates in qPCR analysis with primers specific for *Nrarp* or *Prdm16* TSS; *y-axis*: fold enrichment levels calculated according to the Pfaffl equation using *Thp1* promoter region as internal control, are represented by bars and plotted as the mean ± Standard Deviation of the measurements; a value of 1 indicates no enrichment; data shown are the results of three independent experiments; °: *p* ≤ 0.05 by Student’s *t*-test.

Next, we assessed the recruitment of IKAROS, CHD4 and BRG1 to genes characterized by the ‘desensitize effect’, whereby IKAROS deletion impairs the NOTCH-associated gene activation. *Prdm16* and *Nrarp* genes were selected for this analysis. PRDM16 is an important factor involved in the homeostasis of hematopoietic stem/progenitor cells [73]. It has been identified as a fusion partner in AML [74,75] and its oncogenic behavior seems to be highly dependent of its N-terminal PR-domain [76]. NRARP is a negative feedback regulator of the NOTCH pathway [77]. It also behaves as an oncogene, being overexpressed in breast, liver, thyroid and non-small cell lung cancers [78–81] and in several T-ALL and CLL cell lines upon NOTCH induction [82,83]. Interestingly, IKAROS and NICD can associate with *Nrarp* and *Prdm16* TSS (S8A and S4B Figs). RNA-seq analysis revealed that *Nrarp* and *Prdm16* belong to the group of genes whose expression upon NOTCH induction is affected by IKAROS deletion (Fig 7A). ChIP assays with CHD4 and BRG1 antibodies demonstrated that CHD4 is recruited to *Nrarp* and *Prdm16* TSS, and that this binding is enhanced in Ik^WT^ cells upon NOTCH pathway activation (Ik^WT^/OP9-DL1; Fig 7B), whereas BRG1 recruitment is higher in IKAROS-deleted and NOTCH-activated cells (Ik^Null^/OP9-DL1; Fig 7C). These results suggest that CHD4 and BRG1 are needed to enhance *Prdm16* and *Nrarp* gene expression upon NOTCH induction in Ik^WT^ cells, but that in Ik^Null^ cells, the absence of IKAROS prevents CHD4 recruitment to these genes and their expression upon NOTCH induction. The recruitment of IKAROS-CHD4 to *Prdm16* and *Nrarp* TSS in Ik^WT^ Ter119^+^ cells (Figs S8A and 7B) suggests that IKAROS and CHD4 are required to organize chromatin at regulatory regions for rapid transcriptional activation. Bivalent chromatin characterized by H3K4me3 and H3K27me3 marks, is associated to poised transcription and thereby, rapid transcriptional activation [64]. ChIP assays showed H3K4me3 and H3K27me3 enrichment at *Prdm16* and *Nrarp* chromatin, and significant reduction of H3K4me3 (but not H3K27me3) levels in Ik^Null^/OP9 as well as Ik^Null^/OP9-DL1 cells (S8B Fig). Furthermore, sequential ChIP analysis indicates that IKAROS is important for the organization of bivalent chromatin characterized by the co-occupancy of H3K4me3 and H3K27me3 to the TSS region of these desensitized genes (S8C Fig).

Thus, based on our results we propose that, at genes characterized by the desensitize effect, IKAROS favours the organization of a poised transcription structure marked by bivalent chromatin and thereby, promotes rapid transcriptional activation upon NOTCH signaling.

### IKAROS influences NOTCH targets genes in hematopoietic progenitors and in erythroid cells

Bivalent chromatin and priming of transcriptional activation can be set in hematopoietic stem/progenitor cells (HS/PC) and maintained during cellular differentiation [84–89]. IKAROS is expressed and is an important regulator of genes in HS/PC [37,41]. Accordingly, we observed that IKAROS controls *Cdkn1a* and *Prdm16* gene expression in fetal liver-derived hematopoietic progenitor (lin^-^) cells (S9A Fig). Thus, we tested whether the additive and desensitize effects observed in the Ik^Null^ erythroid cells originate from the absence of IKAROS in HS/PC or can be triggered by the ablation of IKAROS at later stages of erythroid differentiation. To address this issue, non-target siRNA (Ik^NT^: expressing IKAROS at wild type level) or IKAROS knock-down (Ik^KD^) e14.5 fetal liver erythroid cells were co-cultured for 48h on OP9 or OP9-DL1 cells. Less than 40% of *Ikaros* mRNA remains in the Ik^KD^/OP9 or Ik^KD^/OP9-DL1 *vs* Ik^NT^/OP9 or Ik^NT^/OP9-DL1 cells (S9B Fig). Next, we performed a comparative analysis of the expression levels of *Cdkn1a*, *Tp53*, *Nrarp* and *Prdm16* genes in Ik^KD^/OP9 or Ik^KD^/OP9-DL1 *vs* Ik^NT^/OP9 or Ik^NT^/OP9-DL1 cells. The results obtained indicate that, as observed in erythroid cells derived from Ik^Null^ mice, IKAROS knock-down induced by siRNA transfection in erythroid cells favors the additive (*Cdkn1a* and *Tp53* genes) or the desensitize (*Nrarp* and *Prdm16* genes) effect upon NOTCH induction (S9C Fig).

To confirm that the NOTCH induction worked as expected in the siRNA ‘nucleofected’ erythroid cells, the expression levels of *Cxcl1* and *Mmp23*, two gene responding to NOTCH pathway induction but not to variation of IKAROS expression levels (RNA-seq analysis; GEO accession ID: GSE122951), were investigated. The expression of these two genes does not vary between Ik^NT^ (wild type IKAROS expression levels) and IKAROS knock-down cells, and their overexpression solely depends on NOTCH induction upon OP9-DL1 co-culture (S9D and S9E Fig).

Together, these results suggest that IKAROS provides the adequate regulation of NOTCH target genes in hematopoietic progenitor as well erythroid cells.

### IKAROS is required to prevent the overexpression of P21^WAF1/CIP1^ upon DNA damage induction

**Fig 8:**
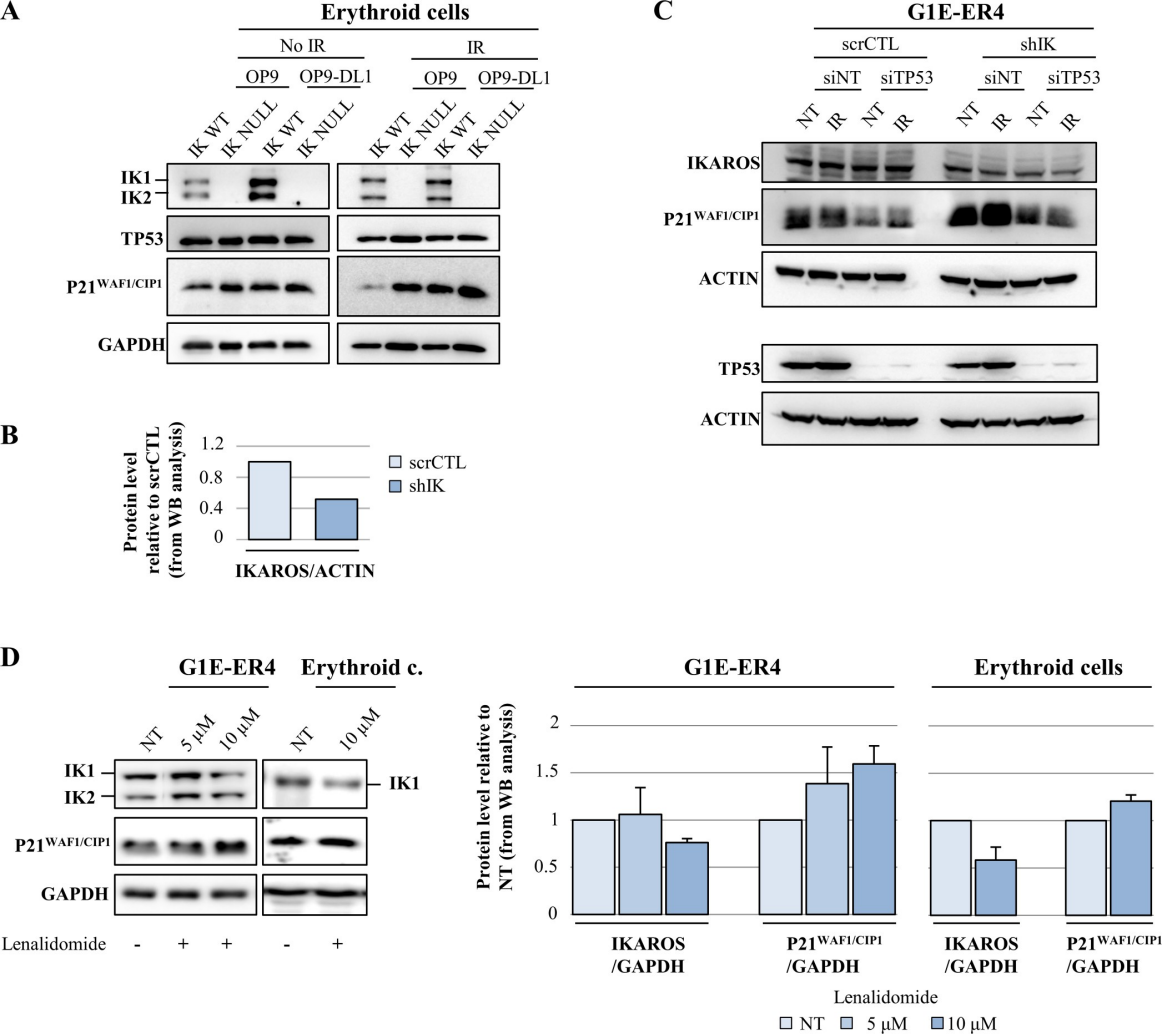
IKAROS is required to prevent the overexpression of P21^WAF1/CIP1^ upon DNA damage induction. Western blot analyses of IKAROS, TP53, P21^WAF1/CIP1^ and GAPDH or ACTIN (loading controls) expression in total cell lysates. All experiments were performed in triplicate and representative results are shown in panels A-D. (A) Ik^WT^ or Ik^Null^ Ter119^+^ cells were harvested after 48h of co-culture with OP9 or OP9-DL1 cells, irradiated (3.5 Gy) and collected 3h post-irradiation. (B) Protein expression; the relative expression levels of IKAROS in G1E-ER4 cells stably transfected with either scrCTL (non-target control) or shIK (IKAROS shRNA), were obtained by dividing the densitometric values (calculated using the Multi Gauge V3.0 software; right panel) of IKAROS bands by the values of ACTIN bands (IK/ACTIN ratios); *y* axis: IKAROS/ACTIN protein level in shIK relative to scrCTL. (C) G1E-ER4 cells transfected either with scrCTL (non-target control) or shIK (IKAROS shRNA); stable clones were transiently transfected with either siNT (control) or siTP53 and irradiated (3.5 Gy); cells were collected 3h post-irradiation. (D) G1E-ER4 or erythroid cells were treated with 5 μM or 10 μM lenalidomide; cells were collected 24h after the treatment; the relative expression levels were obtained dividing the densitometric values (calculated using the Multi Gauge V3.0 software) of IKAROS bands by the values of GAPDH bands (IK/GAPDH ratios) or P21^WAF1/CIP1^ bands by the value of GAPDH bands (P21^WAF1/CIP1^/GAPDH ratios); relative expression levels are represented by bars and plotted as the mean ± Standard Deviation of the measurements (right panel); *: p ≤ 0.05 by Student’s t-test; IK1: IKAROS splicing isoform 1; IK2: IKAROS splicing isoform 2.

Having established the role of IKAROS as regulator of *Tp53* and *Cdkn1a* genes, we then questioned the biological relevance of such regulation upon induction of DNA damage signaling that induces TP53 and P21^WAF1/CIP1^ activation [90]. The levels of IKAROS, TP53 and P21^WAF1/CIP1^ were analyzed in Ik^WT^ and Ik^Null^ Ter119^+^ cells co-cultured with OP9 or OP9-DL1 cells exposed to ionizing radiation (IR; 3.5 Gy). P21^WAF1/CIP1^ levels increased in IKAROS depleted cells whether or not exposed to IR, although IR exposure and NOTCH signaling induced the highest protein levels (Figs 3B and 8A). To address the importance of TP53 for IKAROS-induced P21^WAF1/CIP1^ overexpression, we knocked down *Tp53* or *Tp53* together with *Ikaros* in the erythroid model cell line G1E-ER4 (Fig 8B and 8C). As in Ik^Null^ Ter119^+^ cells, *Ikaros* knock-down in G1E-ER4 cells results in overexpression of P21^WAF1/CIP1^, particularly upon cell irradiation (Fig 8C). Though to a lesser extent, *Tp53* and *Ikaros* double knock-down induced P21^WAF1/CIP1^ overexpression, when compared with *Tp53* knock-down only. Thus, TP53 is required for maximal P21^WAF1/CIP1^ overexpression after irradiation, but IKAROS has an effect by itself on P21^WAF1/CIP1^ regulation in TP53 depleted cells (Fig 8C). To assess the potential clinical importance of IKAROS for P21^WAF1/CIP1^ expression, we tested the effect of lenalidomide on P21^WAF1/CIP1^ levels. Lenalidomide is a thalidomide homologue that promotes ubiquitination and proteasomal degradation of IKAROS as well as other IKZF family members [91,92], and is used for the treatment of patients with myelodysplastic syndrome or multiple myeloma [93,94]. As presented in Fig 8D, lenalidomide treatment of our erythroid cell models (G1E-ER4 and erythroid cells) led to IKAROS depletion and P21^WAF1/CIP1^ overexpression, suggesting that the induction of apoptosis imposed by lenalidomide treatment of hematopoietic cells [81] is likely related to the regulation of *Cdkn1a*/P21^WAF1/CIP1^ forced by IKAROS.

Thus, IKAROS regulation of *Cdkn1a*/P21^WAF1/CIP1^ (i) can occur in association with TP53 or be mediated by IKAROS alone; (ii) is important upon NOTCH induction but is also highly relevant to control *Cdkn1a* induction upon genotoxic insults, *e*.*g*., IR exposure.

## Discussion

The IKAROS-mediated repression of NOTCH target genes is critical at early stages of lymphopoiesis. In here, we demonstrate that IKAROS, not only represses NOTCH target genes in the absence of NOTCH signaling, it can also restrain their activation upon NOTCH induction. This phenomenon was termed the ‘additive effect’ since the absence of IKAROS added to NOTCH induction results in the atypical overexpression of these genes. Our study also revealed the existence of another group of NOTCH target genes, which require IKAROS for transcriptional activation upon NOTCH signaling. These genes, which are not responsive to NOTCH induction in IKAROS depleted cells, are characterized by what we named the ‘desensitize effect’. To both groups of genes IKAROS is required for efficient recruitment of the remodeling ATPase CHD4 and bivalent chromatin organization associated with priming of transcriptional activation. Thus, IKAROS controls NOTCH target gene expression and promotes an adequate response to stress imposed by NOTCH induction or genotoxic insults.

### IKAROS influences the recruitment of chromatin remodelers to NOTCH target genes

Gene expression is precisely regulated to maintain cell functions, specificity, and viability. Cellular stress, imposed by modification of cell homeostasis, infection, or genotoxic agents, activates intracellular signaling pathways including NOTCH, and provides the adequate response to protect the cell. Nonetheless, uncontrolled activation of NOTCH target genes, especially oncogenes, can disrupt cell functions and favor carcinogenesis. IKAROS is known to downregulate or silence many NOTCH target genes. It then prevents their activation in the absence of signaling. Moreover, the additive effect described in here and detected at multiple NOTCH targets, indicates that IKAROS controls the extent of the activation/expression levels of these genes upon NOTCH induction. This view is supported by ChIP-seq data, which indicate that recruitment of IKAROS to multiple NOTCH target genes can be maintained upon NOTCH signaling and gene activation [7]. The mechanism(s) used by IKAROS to control the expression levels of NOTCH target genes upon signaling remains to be fully elucidated. Nonetheless, the fluctuation of CHD4 and BRG1 recruitment to the NOTCH target genes studied suggests that: (i) IKAROS largely controls recruitment of these chromatin remodelers; and (ii) the timely recruitment of both remodelers is required for gene-controlled activation and expression. Indeed, the overexpression imposed by the additive effect of IKAROS depletion and NOTCH signaling is linked to variations in the recruitment of chromatin remodeling activities to NOTCH target genes. According to this view, IKAROS and CHD4 are required to prevent atypical gene overexpression upon NOTCH induction by restraining BRG1 recruitment to genes characterized by the additive effect. However, to *Nrarp* and *Prdm16* genes, which are characterized by the desensitize effect, IKAROS and CHD4 recruitment precedes and is maintained upon BRG1 recruitment and gene activation. Then, the simultaneous presence of CHD4 and BRG1 is associated to full transcriptional activation upon NOTCH induction. Together with chromatin studies, these results reinforce the importance of IKAROS-mediated CHD4 activity for chromatin remodeling and promoter organization, which cannot be compensated by BRG1 recruitment. Hence, for both classes of genes, chromatin remodeling and transcriptional priming imposed by IKAROS and CHD4 are required for the physiological transcriptional activation of target genes upon induction of the NOTCH pathway.

The proximity between the nucleosome remodeling ATPases CHD4 and BRG1, which are best described as components of the NuRD and P/BAF complexes, has been demonstrated in osteosarcoma and hematopoietic cells [95,96]. Furthermore, genome wide analysis of CHD4 and BRG1 chromatin binding suggests their potential co-recruitment to genes repressed in embryonic stem cells [97–99]. However, in other cells, their sequential recruitment is required for the timely regulation of multiple genes [100]. For instance, the epithelial-mesenchymal transition in oral cancer cells is regulated by the sequential recruitment of these remodeling complexes to common genes, which facilitates the switching of their transcriptional state [101]. Similar to what we observed at *Cdkn1a* and *Tp53* genes (Fig 6), NuRD was reported to antagonize recruitment and activation of the BAF complex [101]. The opposing activities of CHD4/NuRD and BRG1/BAF could therefore contribute to fine-tune gene expression in response to external signals [97,102]. However, the co-recruitment of CHD4 and BRG1 to genes displaying the desensitize effect does not corroborate with these observations whereby CHD4/NuRD acts as a transcriptional repressor and prevents the recruitment/activity of BRG1/BAF. The proximity and co-localization of these remodelers to transcriptionally active genes rather suggest that they might exert complementary actions to regulate gene expression. The cooperation between remodelers is required for proper chromatin organization and is best described during DNA repair (reviewed in [103]). Different remodelers including CHD4, BRG1 and SNF2H are recruited to DNA double strand breaks (DSBs) and participate to DSB detection and repair by homologous recombination. The joint recruitment of remodelers has also been described to transcriptionally active genes, such as *hsp70* gene in *Drosophila* whereby the SWI/SNF complex and an ISWI-related complex are simultaneously located at the active promoter [104]. The complementary activity of remodelers can indeed be required for recruitment of different transcription factors or to control specific steps of transcription including poised organization, transcriptional initiation, elongation and/or termination [105–107].

### IKAROS establishes bivalent chromatin and poised transcription of NOTCH target genes

Histone post-translational modifications were tested to define the mechanism(s) used by IKAROS to influence the chromatin organization and regulation of the NOTCH target genes of both above-mentioned groups. With the exception of the transcriptionally active *Tp53* gene, *Cdkn1a*, *Nrarp* and *Prdm16* genes were characterized by H3K4me3 and H3K27me3, which are histone post-translational modifications that usually characterize bivalent chromatin and mark genes primed/poised for transcriptional activation [64]. According to the canonical model, NOTCH activation leads to NICD translocation to the nucleus where the binding to NOTCH target genes is dynamic and frequently influenced by IKAROS [7,11,13,14,108]. In the absence of IKAROS, NOTCH activation *per se* associates with increased H3K79me2 levels at *Cdkn1a* and *Tp53* TSS and ORF. However, NOTCH activation does not perturb the levels of H3K4me3 or H3K27me3 marks, which require the additional effect of IKAROS deletion to significantly change. According with this observation, *Cdkn1a* and *Tp53* gene expression increases upon NOTCH pathway activation, although their overexpression is more important in cells that also lack IKAROS protein. Of note, sequential ChIP analyses demonstrate the importance of IKAROS for the establishment of bivalent chromatin domains characterized by the co-occupancy of H3K4me3 and H3K27me3 to *Cdkn1a*, *Nrarp* and *Prdm16* genes, prior to their activation upon NOTCH induction. Previous reports suggested that IKAROS could be associated to bivalent chromatin at the *CD8* locus in CD8^-^/CD4^-^ double negative thymocytes, hence before transcriptional activation of the *CD8* gene [109]. IKAROS then remains associated with chromatin during thymocyte differentiation to CD8^+^/CD4^+^ double positive stage, when *CD8* is transcriptionally activated. Furthermore, Ding et al. [110] proposed that IKAROS can have a pioneering activity by favoring chromatin organization granting accessibility of other regulators to enhancers and gene promoters during lymphoid cell differentiation. Whether IKAROS could influence the timely recruitment of CHD4 and BRG1 to these genes remains unclear.

Unlike the other NOTCH target genes that we have characterized, *Tp53* has a transcriptional activity that is further enhanced by NOTCH induction. The overexpression of *Tp53* related to the additive effect is characterized by an increase of H3K79 methylation, which is associated with productive transcriptional elongation [69,111,112]. H3K79 methylation is mediated by DOT1L, a methyltransferase important for transcription elongation [113]. The importance of DOT1L for the additive effect was demonstrated upon treatment with the DOT1L inhibitor EPZ004777. DOT1L inhibition affects *Tp53* and also *Cdkn1a* overexpression in NOTCH-activated cells whereby IKAROS had been deleted, thus suggesting that IKAROS can restrain DOT1L activity and productive transcription elongation of these genes. In support of this finding, it has been shown that DOT1L activity can correlate with high-level gene expression and the DOT1L yeast homolog, Dot1, genetically and physically interacts with PAF1, a potent regulator of transcription elongation [113]. How IKAROS can provide a rate limiting DOT1L activity to NOTCH targets is presently unknown, but could be related to the composition of the Pol II transcriptional elongation complex or to the chromatin organization at transcribed region of target genes. Indeed, IKAROS modulation of CHD4/NuRD and BRG1/BAF recruitment to chromatin could be pivotal since in addition to their well described function in regulating gene activation and repression, both remodeling complexes are reported to directly influence transcriptional elongation [17,23,104,114]. In fact, the CHD4/NuRD complex can interact with P-TEFb, which enhances the release of promoter-proximal paused Pol II [18,23] and BRG1/BAF, which promotes chromatin remodeling associated with Pol II transcription elongation of the HSF1 target gene *Hsp70* in mammalian fibroblasts [104,114].

**Fig 9:**
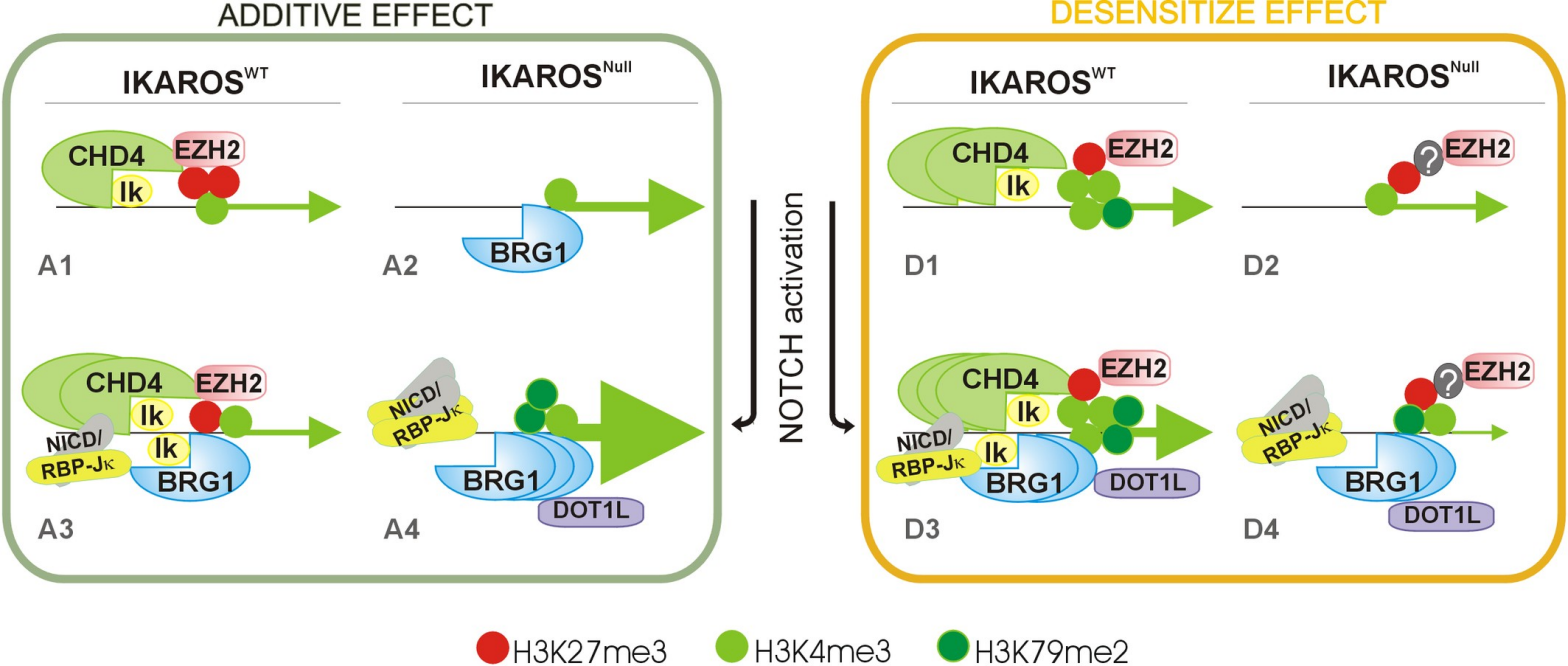
Proposed model of IKAROS measured response of NOTCH target genes upon NOTCH signaling in erythroid cells. This model summarizes the contribution of IKAROS in the regulation of NOTCH target genes. Two groups of genes were identified and investigated: genes overexpressed when IKAROS is absent and the NOTCH pathway is induced (‘additive effect’, left panel); and genes downregulated by the absence of IKAROS combined to the NOTCH pathway induction (‘desensitize effect’, right panel). Based on the results presented in here, IKAROS and NuRD are first and foremost required for priming and transcriptional poised chromatin organization of these genes. Both, additive as well as desensitize genes share common regulation when IKAROS is present and the NOTCH pathway is not induced (A1, D1). IKAROS then binds chromatin and establishes competence for CHD4/NuRD (CHD4) and EZH2/PRC2 (EZH2) recruitment. H3K27me3, which is produced by the methyltransferase EZH2, marks the chromatin of *Cdkn1a*, *Prdm16* and *Nrarp*, but not the chromatin of the transcriptionally active *Tp53* gene. Whether EZH2 is recruited or the H3K27me3 mark removed rapidly from this transcriptionally active gene, is not known. H3K4me3, which results from the activity of MLL/SETD1 methyltransferases, also characterizes the four target genes when IKAROS is present and the NOTCH pathway is not induced. Together, H3K27me3 with H3K4me3 typify bivalent chromatin associated to many primed genes and transcription poised organization. In the absence of IKAROS and/or induction of NOTCH (A2-A4 and D2-D4), the chromatin association of IKAROS and CHD4/NuRD can hinder efficient/optimal recruitment of BRG1/BAF and restrict transcriptional enhancement; the substantial recruitment of CHD4/NuRD to chromatin requires IKAROS but can be further enhanced upon NOTCH activation (NICD might also interact with NuRD [8]); the high expression or overexpression of additive and desensitize genes is associated with increasing H3K79me2, which is deposited by DOT1L and is associated to the transcriptional elongation machinery. The regulation of additive and desensitized gene differs in the ratio between the H3K27me3 and H3K4me3 that characterizes their chromatin upon NOTCH pathway induction and/or the absence of IKAROS. In summary, the regulation of these genes is similar in IKAROS expressing cells prior to NOTCH pathway induction. After NOTCH induction, the regulation of additive genes and desensitize genes diverge. Thus, we propose that IKAROS is a pioneer transcription factor granting chromatin organisation and competence for gene expression, which are critical for rapid and physiological transcription induction upon signaling. Grey line: gene TSS (transcriptional start site); Ik: IKAROS; green arrows: active transcription, which is proportional to the size of the arrow; orange filled dot; histone H3K27me3; light green filled dot histone H3K4me3; dark green filled dot: histone H3K79me2;?: unknown additional factors.

In summary, our results support a model whereby the IKAROS-CHD4 association does not account for individual gene activation or repression, but rather assures appropriate chromatin conformation and poised transcriptional organization required for transcriptional activation upon NOTCH induction (Fig 9). Without the dominant contribution of IKAROS, chromatin ‘pre-setting’ either before NOTCH induction or genotoxic insults, will be abnormal and most likely influenced by other regulatory elements that can: (i) promote BRG1 recruitment and atypical overexpression (additive effect); or (ii) preclude BRG1 recruitment and transcriptional activation (desensitize effect) of the NOTCH target genes. At NOTCH target genes, IKAROS largely controls the timely recruitment of the chromatin remodelers CHD4 and BRG1, and thereby, gene activation/expression levels. The results presented here provide a new perspective on the critical role played by IKAROS to promote adequate and controlled cell response to stress, whether imposed by NOTCH induction or genotoxic insults.

## Materials and methods

### Ethics statement

Animal experiments were conducted in accordance with the Canadian Council on Animal Care (CCAC) guidelines and approved by the Maisonneuve-Rosemont Hospital animal care committee (protocol number: 2015–15).

### Mouse line

The Ik^Null^ mouse model is characterized by the deletion of the last translated exon of the *Ikaros* (*Ikzf1*) gene, which results in protein instability and the absence of IKAROS protein in all tissues [30]. Heterozygous male and female were bred, embryonic day 14.5 (e14.5) homozygote Ik^WT^ (IKAROS wild-type) or Ik^Null^ embryos were harvested and fetal livers were isolated. Since at this developmental stage the fetal liver contains nearly exclusively (~80%) erythroid cells [115,116], e14.5 fetal liver-derived cells are referred to as erythroid cells throughout the paper. Animals were sacrificed by cervical dislocation. All mutant embryos were compared with wild type littermates, to reduce difference in genetic background or developmental stage. Fetal liver DNA was extracted with DNAzol reagent (ThermoFisher) and samples were genotyped by PCR as described [30]. Animal experiments were conducted in accordance with the Canadian Council on Animal Care (CCAC) guidelines and approved by the Maisonneuve-Rosemont Hospital animal care committee.

### RNA-sequencing and analysis

Ik^WT^ or Ik^Null^ e14.5 fetal liver cells were co-cultured for 48h on OP9 or OP9-DL1 stromal cell lines. Cells were then washed, resuspended in PBS-5% heat-inactivated FBS and Ter119^+^ cells were sorted by high-speed fluorescence-activated cell sorting (FACS). RNA from Ik^WT^/OP9, Ik^WT^/OP9-DL1, Ik^Null^/OP9 and Ik^Null^/OP9-DL1 Ter119^+^ cells was isolated by Trizol (ThermoFisher) extraction and purified with the RNeasy column kit (Qiagen) according to manufacturer instructions. RNA was further purified with mRNA enrichment with oligodT beads. The library was performed with the TruSeq RNA Sample Preparation kit (Illumina). Samples were sequenced on the HiSeq2000 Illumina sequencer following standard procedures. mRNA profiles were generated in biological triplicate, using Illumina cBot 2 System. The quality of the raw reads was assessed with FASTQC. Reports show no pool imbalance. After examining the quality of the raw reads, no trimming was deemed necessary. The reads were aligned to the GRCm38/mm10 genome with TopHat. The raw alignment counts were calculated with htseq-count. The RNA-seq results were normalized with the DESeq package [47]. Briefly, the read count was normalized by size factor that was calculated by median ratio of gene counts relative to geometric mean per gene. The log2 fold change is an estimate of the fold change between the conditions, based on the distribution of the reads. qRT-PCR validation was performed using specific primer sets in real-time PCR with SYBR Green.

### Cell culture

G1E-ER4 (GATA-1 knockout ES-derived pro-erythroblast cell line expressing an inducible GATA-1-ER protein) cells [66–68] were cultured in Iscove’s modified Dulbecco’s medium (IMDM; ThermoFisher) containing 13% fetal bovine serum (FBS; Sigma), 1.7% penicillin-streptomycin (Wisent), 1.8 U/ml erythropoietin (Eprex), 1.1 mM 1-thioglycerol (Sigma), and 0.5% conditioned medium from a Kit ligand-producing CHO cell line.

To produce IKAROS knock-down clones, G1E-ER4 cells were infected with MISSION pLKO.1-puro vector (Sigma) containing short hairpin RNA (shRNA) specific to *Ikaros* mRNA (shIK) or a nonspecific scrambled shRNA (scrCTL). Positive cells were selected with Puromycin (2 μg/ml). OP9-DL1 cells are murine bone marrow stromal OP9 cells engineered to express constitutively the NOTCH ligand DELTA-like 1 (DL1). These cells were used in a co-culture system to activate the NOTCH signaling in e14.5 mouse fetal liver erythroid cells. OP9 and OP9-DL1 cells were seeded in α-Minimum Essential Medium (α-MEM; ThermoFisher) for four days. Then, Ik^WT^ or Ik^Null^ fetal liver cells were layered onto OP9 and OP9-DL1 in α-MEM added with 2 U/ml erythropoietin (Eprex) and cultured for 48h. Where indicated, cells were treated with the DOT1L inhibitor EPZ004777 (Calbiochem), at a concentration of 3 μM for 48h or with equal volume of DMSO (diluent control).

### siRNA nucleofection

Knock-down experiments with small interfering RNA (siRNA) were performed in G1E-ER4 cells or e14.5 fetal liver erythroid cells (Ik^KD^ cells). The non-target control siRNA (siNT) and *Tp53* siRNA (siP53) were purchased from Dharmacon (siGENOME SMARTpool siRNA); *Ikaros* pre-designed siRNA (siIK) (SASI_Mm01_00065776; SASI_Mm01_00065777; SASI_Mm02_00287954) were purchased from MilliporeSigma. For each nucleofection, 3×10^6^ G1E-ER4 cells or 10^6^ erythroid cells were washed PBS and resuspended in 100 μl of Ingenio Solution (Mirus). Next, 25 μl (20 μM) of either siNT or siP53 (for G1E-ER4 cells) or 5 μg of siNT or siIK (for erythroid cells) were added to the cell suspension, which was immediately nucleofected (Amaxa Nucleofector II, Lonza) with program G-016 (G1E-ER4 cells) or X-001 (erythroid cells) according to the literature [117] and manufacturer’ instructions.

### Cell irradiation

E14.5 fetal liver or G1E-ER4 cells were irradiated with a dose of 3.5 Gy, using a gamma cell irradiator. Cells were maintained at 37°C and 5% CO2 and harvested after 3h-incubation.

### Protein analysis

For Western blot assays, cells were lysed in Laemmli buffer (1 mM PMSF, 150 mM NaCl, 50 mM Tris HCl pH 6.8, 10% glycerol, 2% sodium dodecyl sulfate, 0.1% bromophenol blue, 2.5% β-mercaptoethanol), then sonicated, boiled for 10 minutes, migrated on SDS-polyacrylamide gel and transferred to nitrocellulose membrane (Bio-Rad). IKAROS (Cell signaling #D6N9Y), P21WAF1/CIP1 (BD Biosciences #556431), TP53 (Santa Cruz #393031), GAPDH (MilliporeSigma) or ACTIN (NeoMarkers) and horseradish peroxidase (HRP)-conjugated antibody (Jackson) were used for immunoblotting. Proteins were detected with Clarity Western enhanced chemiluminescence substrate (ECL; Bio-Rad) with a Fujifilm LAS-4000 luminescent image analyzer (GE Healthcare).

### Quantitative reverse transcription PCR (qRT-PCR)

One to three million cells were washed in PBS and total RNA was isolated with TRIzol reagent (ThermoFisher). cDNA was generated using the All-in-One cDNA Synthesis SuperMix according to manufacturer’s instructions (Bimake). Quantitative PCR was carried out on an Applied Biosystems machine (Thermo-Fisher) using SYBR Green qPCR Master Mix (Biotool). Relative transcript levels were calculated using *Hprt* or *Actin* as control. To compare gene expression levels between conditions, results are presented as the ratio of the expression of each gene relative to the control expression. Where indicated, results were expressed as a fold change relative to the control. Quantitative analysis was performed according to Pfaffl’s method [118]. Primer sequences are detailed in S1 Table.

### ChIP-qPCR assay

Cells were fixed in PBS/1.5 mM Ethylene glycol bis(succinimidyl succinate) (EGS) for 30 minutes, at room temperature, with gentle agitation and protected from light. Cells were complementary cross-linked for 5 minutes with 1% formaldehyde (Sigma) and fixation was stopped with Glycine (20 mM final) for 10 minutes. Cells were resuspended in SDS lysis buffer (1% SDS; 10 mM EDTA; 50 mM Tris-HCl pH 8.1) for 10 minutes and sonicated (Branson-Digital Sonifier) in order to obtain chromatin shear ranging 300 to 500 bp. Samples were then centrifuged, diluted in dilution buffer (0.01% SDS; 1.1% Triton; 1.2 mM EDTA; 16.7 mM Tris-HCl pH 8.0; 165 mM NaCl), pre-cleared with protein A Sepharose beads (MilliporeSigma) and incubated overnight with H3K4me3 (Abcam#8580), H3K27me3 (Milipore#17–622), H3K36me3 (Milipore#17100–32), H3K79me2 (Cell Signaling#5427), IKAROS (Santa-Cruz#13039), NOTCH/NICD (Abcam#8925) antibodies or isotype-matched immunoglobulins. Protein-DNA complexes were retrieved with protein A Sepharose beads (MilliporeSigma) and sequentially washed with the following buffers: low salt (0.1% SDS; 1% Triton; 2 mM EDTA; 20 mM Tris HCl pH 8; 150 mM NaCl), high salt (0.1% SDS; 1% Triton; 2 mM EDTA; 20 mM Tris HCl pH 8.0; 500 mM NaCl), LiCl (0.25 M LiCl; 1% NP-40; 1% Sodium Deoxycholate; 1 mM EDTA; 10 mM Tris-HCl pH 8.0) and TE (1 mM EDTA; 10 mM Tris HCl pH 8.0). After de-crosslinking and proteinase K digestion, the genomic DNA was purified and used as template in qPCR reactions.

Quantitative PCR (qPCR) were carried out as reported in Bottardi et *al*. [119] with minor modifications. About 1/30 of immunoprecipitated and 1/10 of unbound (input) material was used as a template for qPCR using SYBR Green (Bimake) on an ABI 7300 Fast real-time PCR system (Applied Biosystem). Quantification was performed according to the 2^-ΔΔCt^ method [120] where ΔΔCt = ΔCt (test)—ΔCt (reference); ΔCt (test) = Ct (ChIP test)–Ct (input test); ΔCt (reference) = Ct (ChIP reference); test corresponds to the gene of interest; reference, to *Thp* promoter. All data showed are the results of at least four independent ChIP experiments with qPCR from each ChIP performed in triplicate and averaged (standard deviation). To correctly interpret the amplification values obtained by qPCR, the efficiency of all primer sets was carefully checked and primer pairs displaying an amplification efficiency ranging from 95 to 102% were chosen. Primer sequences are detailed in S2 Table.

### Ter119/CD71 fetal liver cell sorting

Ik^WT^ or Ik^Null^ e14.5 fetal liver cells were washed and resuspended in PBS-5% heat-inactivated FBS. Cells were sequentially incubated for 30 minutes on ice with rat anti-Ter119 hybridoma, anti-rat Fluorescein Isothiocyanate (FITC)-conjugated (BD-Pharmingen) and Phycoerythrin (PE)-conjugated anti-CD71 (BioLegends) antibodies. Cells were sorted by high-speed fluorescence-activated cell sorting (FACS Vantage; Becton, Dickinson).

### Lineage negative hematopoietic progenitor (lin^-^) cell purification

Ik^WT^ or Ik^Null^ e14.5 embryos were harvested, and fetal livers were collected and mechanically dissociated in 1X PBS, 5% FBS to generate a single-cell suspension and then centrifuged. Mature erythrocytes were lysed as follows: cell pellet was resuspended in 10 ml of 0.2% NaCl and after 40 second, 10 ml of 1.6% NaCl was added to re-establish osmolarity. Cells were centrifuged at 400g for 10 minutes, resuspended in 1X PBS, 5% FBS, passed through a 40 μm strainer to remove debris and then centrifuged for 5 minutes at 250g at 4°C. Lin^-^ cells were obtained using the easySep mouse hematopoietic progenitor enrichment kit (StemCell Technology). RNA extraction and qRT-PCR analysis were carried out as described above (‘Quantitative reverse transcription PCR (qRT-PCR)’). Lin^-^ cell purity and composition was assessed by *ex-vivo* clonogenic assays in complete methylcellulose medium (MethoCult M3434 StemCell Technology) and scored under contrast microscopy at day 14 [23].

### Sequential ChIP (re-ChIP)

Ik^WT^ or Ik^Null^ e14.5 embryos were harvested, and fetal livers were isolated. Erythroid cells (20 X 10^6^ cells per re-ChIP) were fixed in PBS, 1% formaldehyde (MilliporeSigma) for 10 minutes at room temperature with gentle agitation and fixation was quenched with Glycine (20 mM final) for 10 minutes. The first round of precipitation was carried out as described above (‘ChIP-qPCR assay’). After the second wash in TE buffer, immunoprecipitated complexes were eluted twice in buffer E (50 mM Tris-HCl pH 8.0; 10 mM EDTA; 1% SDS) by gentle shacking at 42°C for 20 min. Eluates were pooled, diluted 1:10 in ChIP dilution buffer and subject to the second round of immunoprecipitation. After an overnight incubation at 4°C, washes, chromatin elution, de-crosslinking, proteinase K digestion and qPCR were carried out as described above (‘ChIP-qPCR assay’). Re-ChIP assays were carried out with H3K4me3, H3K27me3 antibodies or isotype-matched IgG. Fold enrichment levels were calculated according to the “percentage input method” (https://www.thermofisher.com/ca/en/home/life-science/epigenetics-noncoding-rnaresearch/chromatin-remodeling/chromatin-immunoprecipitation-chip/chip-analysis.html).

## Supporting information

S1 Table Oligonucleotides used for qRT-PCR analysis. (DOCX)

S2 Table Oligonucleotides used for ChIP analysis. (DOCX)

S3 Table Genes overexpressed in Ik^WT^ Ter119^+^ upon NOTCH induction. Genes overexpressed in Ik^WT^/OP9-DL1 relative to Ik^WT^/OP9 cells. Log2 ˃ 0.8; FDR < 0.05.
(DOCX)

S4 Table Principal functional annotations of genes listed in S3 Table. (DOCX)

S5 Table Principal functional annotations of the 499 genes specifically overexpressed in Ik^Null^/OP9 cells; log2 ≥ 0.08; *p* < 0.05. (DOCX)

S6 Table Principal functional annotations of the 844 genes specifically overexpressed in Ik^Null^/OP9-DL1 *vs*. Ik^WT^/OP9-DL1 cells; log2 ≥ 0.08; *p* < 0.05. (DOCX)

S7 Table Tumor suppressors and oncogenes included in the list of the 223 genes characterized by the ‘additive effect’ (see Fig 2A). (DOCX)

S8 Table Tumor suppressors and oncogenes included in the list of the 21 genes characterized by the ‘desensitize effect’ (see Fig 2B). (DOCX)

S9 Table List of the 223 genes characterized by the ‘additive effect’ (see Fig 2A). (DOCX)

S1 Fig Expression of the Delta-like 1 ligand in OP9 cells. Western blot analysis of Delta-like 1 ligand (DL1) and ACTIN (loading control) expression in OP9 or OP9-DL1 total cell lysates.
(TIF)

S2 Fig Distribution of the erythroid populations in Ik^Null^ and Ik^WT^ e14.5 mouse fetal livers co-cultured with OP9 or OP9-DL1 cells. Ik^Null^ and Ik^WT^ e14.5 fetal livers were isolated and single-cell suspensions were co-cultured with OP9 or OP9-DL1 cells; cells were collected after 48h and analyzed by flow cytometry on the basis of Ter119 and CD71 expression levels: P1, CD71^med^/Ter119^neg/low^; P2, CD71^high^/Ter119^neg/low^; P3, CD71^high^/Ter119^high^; P4, CD71^med^/Ter119^high^; P1 is enriched in erythroid precursors (BFU-E: burst-forming unit-erythroid and CFU-E: colony-forming unit erythroid), P2 is enriched in proerythroblasts and early basophilic erythroblasts, P3 is enriched in basophilic and chromatophilic erythroblasts, and P4 is enriched in orthochromatic erythroblasts; *x*-axis: cell populations; *y*-axis: percentage of positive cells.
(TIF)

S3 Fig Heatmaps corresponding to the genes overexpressed in Ik^Null^/OP9 or OP9-DL1 vs Ik^WT^/OP9 or OP9-DL1. These heatmaps are complementary to Fig 1B. Colors on the heatmaps (depicted in the color key) represent the relative expression levels of individual genes in the four conditions used for this study (indicated at the bottom of the heatmaps). Unsupervised hierarchical clustering (Pearson correlation, average linkage) over the genes included in each group is presented on the left side of the heatmaps.
(TIF)

S4 Fig Gene expression and NICD recruitment in Ik^WT^ and Ik^Null^ Ter119^+^ cells upon NOTCH induction. (A) Gene expression; the relative expression levels of *Il6* and *Zfp446* genes were measured by qRT-PCR, calculated according to the Pfaffl equation using *Hprt* as internal control and normalized to Ik^WT^/OP9; *y* axis: relative RNA enrichment levels, ratios are represented by bars and are plotted as the mean ± Standard Deviation (SD) of the measurements; data shown are the results of three independent experiments; *: *p* ≤ 0.05 by Student’s *t*-test. (B) Chromatin immunoprecipitation assays in Ik^Null^/OP9 or Ik^WT^/OP9-DL1 cell with NICD antibodies; immunoprecipitated and unbound (input) chromatin samples were used as templates in qPCR analysis with primers specific for *Cdkn1a*, *Tp53*, *Prmd16*, *Nrarp* or *Hes1* transcription start site (TSS) regions; *y-axis*: fold enrichment levels calculated according to the Pfaffl equation using the *Thp1* promoter region as internal control, are represented by bars and plotted as the mean ± SD of the measurements; a value of 1 indicates no enrichment; data shown are the results of three independent experiments; TSS: transcription start site; *: *p* ≤ 0.05 by Student’s *t*-test.
(TIF)

S5 Fig Western blot quantification presented in Fig 4C. This figure provides further information regarding the quantification of the Western blot results presented in Fig 4C. The red boxes indicate selected areas subject to quantification. After background subtraction, the ratio of P21^WAF1/CIP1^/ACTIN was calculated and depicted as graph (bottom part of Fig 4C). The same procedure was used for the quantification of the Western blot results presented in Fig 4D.
(TIF)

S6 Fig Determination of histone post-translational modifications and sequential ChIP analysis carried out to test the co-occupancy of H3K4me3 and H3K27me3 at *Cdkn1a* and *Tp53* TSS. (A) ChIP assays with H3K4me3, H3K79me2, H3K27me3 antibodies (blue) or isotype-matched IgG (grey) were conducted in Ik^WT^ or Ik^Null^ Ter119^+^ cells co-cultured with OP9 or OP9-DL1 cells; immunoprecipitated and unbound (input) chromatin samples were used as templates in qPCR analyses with primers specific for the minor satellite repeat sequence, which is used as the control region for the ChIP results presented in Fig 5; *y-axis*: fold enrichment levels calculated according to the Pfaffl equation using *Thp1* promoter region as internal control, are represented by bars and plotted as the mean ± Standard Deviation of the measurements; a value of 1 indicates no enrichment; data shown are the results of three independent experiments. (B) Sequential ChIP (re-ChIP) assays carried out on erythroid cells isolated from e14.5 Ik^WT^ or Ik^Null^ fetal livers with antibodies directed against H3K4me3, H3K27me3 or isotype-matched IgG. K4/K27 bars: H3K4me3 antibodies were used for the first round of precipitation and H3K27me3 antibodies for the second ChIP; K27/K4 bars: H3K27me3 antibodies were used for the first round of precipitation and H3K4me3 antibodies for the second ChIP. Top panels: immunoprecipitated and unbound (input, I) chromatin samples were used as templates in qPCR analysis with primers specific for *Cdkn1a* or *Tp53* TSS; *y-axis*: fold enrichment levels calculated according to the “percentage input method” (https://www.thermofisher.com/ca/en/home/life-science/epigenetics-noncoding-rnaresearch/chromatin-remodeling/chromatin-immunoprecipitation-chip/chip-analysis.html) are represented by bars and plotted as the mean ± Standard Deviation of the measurements; in the re-ChIP samples, no amplification for *Thp1* promoter region (used as internal control) could be observed; similarly, no amplification for *Cdkn1a* or *Tp53* TSS regions was observed when isotype-matched IgG were used for the second round of precipitation; data shown are the results of three independent experiments; *: *p* ≤ 0.05 by Student’s *t*-test. Bottom panels: PCR samples were resolved on 2% agarose gel. Ab1: antibodies used for the first round of precipitation; Ab2: antibodies or isotype-matched IgG used for the second round of precipitation; I: input.
(TIF)

S7 Fig Effect of DOT1L inhibition on *Cdkn1a* and *Tp53* gene expression in G1E-ER4 cells or Ik^WT^ and Ik^Null^ Ter119^+^ cells upon NOTCH induction. (A) Western blot analysis of H3K79me2 and ACTIN (loading control) in total lysates of G1E-ER4 cells treated for 24h or 48h with 3 μM, 10 μM or 20 μM DOT1L inhibitor (inhDOT1L: EPZ004777) or equal volume of DMSO diluent. (B) Western blot analysis of H3K79me2 and ACTIN (loading control) in total lysates of Ik^Null^ and Ik^WT^ Ter119^+^ cells treated with 3 μM DOT1L inhibitor or equal volume of DMSO and co-cultured with OP9 or OP9-DL1 cells for 48h. (C) ChIP assays with H3K79me2 antibodies or isotype-matched IgG conducted in Ik^WT^ or Ik^Null^ Ter119^+^ cells after 48h of co-culture with OP9-DL1 cells and treatment with 3 μM DOT1L inhibitor or equal volume of DMSO diluent; immunoprecipitated and unbound (input) chromatin samples were used as templates in qPCR analysis with primers specific for *Cdkn1a* or *Tp53* TSS; *y-axis*: fold enrichment levels calculated according to the Pfaffl equation using the *Thp1* promoter region as internal control, are represented by bars and plotted as the mean ± Standard Deviation (SD) of the measurements; a value of 1 indicates no enrichment; data shown are the results of three independent experiments; *: *p* ≤ 0.05 by Student’s *t*-test. (D) Gene expression; the relative expression levels of *Cdkn1a* and *Tp53* genes in Ik^WT^ or Ik^Null^ Ter119^+^ cells after 48h of co-culture with OP9-DL1 cells and treatment with 3 μM DOT1L inhibitor or equal volume of DMSO diluent, were measured by qRT-qPCR, calculated according to the Pfaffl equation using *Hprt* as internal control and normalized to Ik^WT^/OP9 DMSO-treated; *y*-axis: relative fold enrichment levels are represented by bars and plotted as the mean ± SD of the measurements; data shown are the results of three independent experiments; *: *p* ≤ 0.05 by Student’s *t*-test.
(TIF)

S8 Fig Histone post-translational modifications at *Nrarp* and *Prdm16* genes in Ik^WT^ and Ik^Null^ Ter119^+^ cells upon NOTCH induction. (A-B) ChIP assays with IKAROS, H3K4me3, H3K79me2, H3K27me3 antibodies (blue) or isotype-matched IgG (grey) were conducted in Ik^WT^ or Ik^Null^ fetal liver erythroid cells (panel A) or Ik^WT^ or Ik^Null^ Ter119^+^ cells co-cultured with OP9 or OP9-DL1 cells (panel B); immunoprecipitated and unbound (input) chromatin samples were used as templates in qPCR analysis with primers specific for *Nrarp*, *Prdm16* TSS, *Hes1* promoter (IKAROS positive control in panel A); the minor satellite region was the negative control for IKAROS ChIP assay (presented in Fig 3E), and for the H3K4me3, H3K79me2, H3K27me3 ChIP assays (presented in S6A Fig); *y-axis*: fold enrichment levels calculated according to the Pfaffl equation using the *Thp1* promoter region as internal control, are represented by bars and plotted as the mean ± Standard Deviation of the measurements; a value of 1 indicates no enrichment; data shown are the results of three independent experiments; *: *p* ≤ 0.05 by Student’s *t*-test. (C) Sequential ChIP (re-ChIP) assays carried out on erythroid cells isolated from e14.5 Ik^WT^ or Ik^Null^ fetal livers with antibodies against H3K4me3 or H3K27me3. K4/K27: H3K4me3 antibodies were used for the first round of precipitation and H3K27me3 antibodies for the second ChIP; K27/K4: H3K27me3 antibodies were used for the first round of precipitation and H3K4me3 antibodies for the second ChIP. Immunoprecipitated and unbound (input) chromatin samples were used as templates in qPCR analysis with primers specific for *Prdm16* or *Nrarp* TSS; *y-axis*: fold enrichment levels calculated according to the “percentage input method” (https://www.thermofisher.com/ca/en/home/life-science/epigenetics-noncoding-rnaresearch/chromatin-remodeling/chromatin-immunoprecipitation-chip/chip-analysis.html) are represented by bars and plotted as the mean ± Standard Deviation of the measurements; in the re-ChIP samples, no amplification for *Thp1* promoter region (used as internal control) could be observed; as well, no amplification for *Prdm16* or *Nrarp* TSS was observed when isotype-matched IgG were used for the second round of precipitation; data shown are the results of three independent experiments; *: *p* ≤ 0.05 by Student’s *t*-test.
(TIF)

S9 Fig Effect of IKAROS abrogation on *Cdkn1a*, *Tp53*, *Nrarp* and *Prdm16* gene expression in lineage negative hematopoietic progenitor (lin^-^) and erythroid cells. The relative expression level of *Cdkn1a*, *Tp53*, *Nrarp*, *Prdm16*, *Cxcl1* and *Mmp23* genes in Ik^Null^ lin^-^ (A) as well as in Ikaros knock-down (siIK) erythroid cells (B-E) was measured by qRT-PCR, calculated according to the Pfaffl equation using *Actin* as internal control. (A) Ikaros target gene expression values in Ik^Null^ lin^-^ cells were normalized to the values obtained in Ik^WT^ lin^-^ cells. (B, C, D) Ikaros (B) or target gene (C, D) expression values in siIK-transfected erythroid cells (Ik^KD^ in the main text) were normalized to the values obtained in siNT (non-target siRNA)-transfected erythroid cells; (E) expression values in siIK-transfected (left panel) or siNT-transfected (right panel) erythroid cells co-cultured with OP9 or OP9-DL1 cells; gene expression values in siIK/OP9-DL1 or siNT/OP9-DL1 were normalized to the values obtained in siIK/OP9 or siNT/OP9 cells respectively; *y* axis: relative RNA enrichment levels, ratios are represented by bars and are plotted as the mean ± Standard Deviation (SD) of the measurements; data shown are the results of three independent experiments; *: *p* ≤ 0.05 by Student’s *t*-test.
(TIF)

S1 File Genes overexpressed in IkNull/OP9 or OP9-DL1 vs IkWT/OP9 or OP9-DL1. The genes composing the group α, β and χ of Figs 1B and S3 are listed in the excel file. The average mean expression of the RNA-sequencing results obtained from biological triplicate is indicated for each gene in the 4 conditions used for this study.
(XLSX)

S2 File Genes characterized by the additive or the desensitize effect imposed by the absence of IKAROS and the induction of NOTCH. Excel file presenting the list of genes of the two groups. The average mean expression of the RNA-sequencing results obtained from biological triplicate is indicated for each gene in the 4 conditions used for this study.
(XLSX)
