# Supplementary material for: IKAROS is required for the measured response of NOTCH target genes upon external NOTCH signaling
Source: PLoS Genet. 2021 Mar 26;17(3):e1009478. doi: 10.1371/journal.pgen.1009478 (PMC8026084; doi:10.1371/journal.pgen.1009478)
Supplement: S1 Table — (DOCX) [file pgen.1009478.s001.docx]

| Gene | Primer Forward (5’) | Primer Reverse (3’) |
| --- | --- | --- |
| *Cdkn1a* | TGTCTTGCACTCTGGTGTCTG | ATCTGCGCTTGGAGTGATAGA |
| *Tp53* | ACGTGCTCACCCTGGCTAAA | GACACCCTGCTGGGAAGGAG |
| *Hprt* | CACAGGACTAGAACACCTGC | GCTGGTGAAAAGGACCTC |
| *Actin* | ATCGTGGGCCGCCCTAGGCACCA | TCCATGTCGTCCCAGTTGGTAACAA |
| *Il6* | TGGAGTCACAGAAGGAGTGGC | AACGCACTAGGTTTGCCGAG |
| *Zfp446* | AGGAGGCCGCAGTTCTTGTT | GGACTCCGTTGCTGCTGAGA |
| *Cxcl1* | TTGACGCTTCCCTTGGACAT | CTTTGAACGTCTCTGTCCCGA |
| *Mmp23* | AGGCACAGCTGAGCATCATT | GTAGGTGCTGAGAACACGCT |
| *Hes1* | CTCTTCCTCCCATTGGCTGA | GCACCAGCTCCAGATCCTGT |

**Table S1.** Oligonucleotides used for qRT-PCR analysis
