# Supplementary material for: IKAROS is required for the measured response of NOTCH target genes upon external NOTCH signaling
Source: PLoS Genet. 2021 Mar 26;17(3):e1009478. doi: 10.1371/journal.pgen.1009478 (PMC8026084; doi:10.1371/journal.pgen.1009478)
Supplement: S2 Table — (DOCX) [file pgen.1009478.s002.docx]

**Table S2.** Oligonucleotides used for ChIP analysis

| Gene | Primer Forward (5’) | Primer Reverse (3’) |
| --- | --- | --- |
| *Cdkn1a TSS* | ATTCACGTGCAATGGTGTGC | AACTTCTGGCTTCCCAGAGC |
| *Cdkn1a +3kb (ORF)* | TAGAGCTTAGCGCAGAGCGG | GCACTTGGTTCACAAGGACC |
| *Tp53 TSS* | CGACTACAGTTAGGGGGCAC | AGAGGTCTCGTCACGCTCAT |
| *Tp53 +5kb (ORF)* | GAGATTGGCTGGCTGTGACT | TCCCTAGGCTATGAGGTAGGC |
| *Prdm16 TSS* | GCGAAAAGTTGACGAAAGGGG | GCCCACTATTTCAAAAGCCCG |
| *Nrarp TSS* | CTAGCTCTGCGGCAACATGA | CGTCGATGACTGACTGGTGT |
| *Thp.1 (Uromodulin)* | GGTGGATGGTGTGGTCACAAC | GGTCTTGACACACCAGCTTT |
| *Minor satellites* | CATGGAAAATGATAAAAACC | CATCTAATATGTTCTACAGTGTG |
