# Supplementary material for: IKAROS is required for the measured response of NOTCH target genes upon external NOTCH signaling
Source: PLoS Genet. 2021 Mar 26;17(3):e1009478. doi: 10.1371/journal.pgen.1009478 (PMC8026084; doi:10.1371/journal.pgen.1009478)
Supplement: S3 Table — Genes overexpressed in IkWT/OP9-DL1 relative to IkWT/OP9 cells. Log2 ˃ 0.8; FDR < 0.05. (DOCX) [file pgen.1009478.s003.docx]

**Table S3.** Genes overexpressed in Ik^WT^ Ter119^+^ upon NOTCH induction. Genes overexpressed in Ik^WT^/OP9-DL1 relative to Ik^WT^/OP9 cells. Log2 ˃ 0.8; FDR < 0.05.

| Gene ID | Mean Ik^WT^/OP9-DL1 | Mean Ik^WT^/OP9 | log2 Fold Change | p-value |
| --- | --- | --- | --- | --- |
| Dll1 | 374.409654 | 2.4955434 | 5.12548337 | 6.5781E-41 |
| Gzma | 5.96295911 | 0 | 2.20612762 | 1.1803E-05 |
| Col15a1 | 49.2931872 | 6.11287759 | 2.13331756 | 1.9748E-07 |
| Timd4 | 15.7527483 | 1.14563451 | 2.03425605 | 1.7441E-05 |
| Rtn1 | 5.19379225 | 0.41302414 | 1.8978292 | 0.00020869 |
| Col5a3 | 32.868579 | 3.24107695 | 1.89351554 | 2.2942E-05 |
| Cd248 | 25.8382656 | 4.29551122 | 1.80412032 | 1.659E-05 |
| Naalad2 | 5.31916952 | 0 | 1.73919665 | 0.00051657 |
| Spic | 54.6004498 | 13.7754631 | 1.72092092 | 1.0538E-05 |
| Mcpt1 | 66.8600995 | 1.34990889 | 1.7125855 | 0.00016759 |
| Prdm16 | 49.4958878 | 12.747881 | 1.6530614 | 0.00023828 |
| Gzmb | 35.8065236 | 9.47100113 | 1.64289005 | 0.00024902 |
| Emr4 | 22.9281237 | 3.89317257 | 1.63020646 | 0.00016089 |
| Fbn1 | 96.669504 | 22.8031183 | 1.60322398 | 5.6896E-06 |
| Pdgfrb | 60.2114676 | 11.5231987 | 1.59160697 | 0.00014637 |
| Rspo2 | 6.98077957 | 0.95204562 | 1.54101545 | 0.00164776 |
| Col3a1 | 151.385148 | 35.3655904 | 1.53357536 | 6.6842E-05 |
| Cxcl5 | 74.2617979 | 10.1126463 | 1.52115691 | 0.00091317 |
| Mcpt2 | 19.6800847 | 1.5760572 | 1.51520148 | 0.00049571 |
| Wisp2 | 49.7841916 | 7.03236379 | 1.51013464 | 0.00154178 |
| Bank1 | 15.7207982 | 3.59322258 | 1.50259607 | 0.00099099 |
| Abcg3 | 72.5177376 | 17.2968538 | 1.49533374 | 0.00067467 |
| Itga9 | 40.4060238 | 9.77592941 | 1.49324521 | 6.6977E-05 |
| Ccl28 | 3.42471079 | 0 | 1.48330708 | 0.00328461 |
| Adamts2 | 8.34034056 | 0.82604828 | 1.48080601 | 0.00124941 |
| Padi2 | 356.601025 | 113.190666 | 1.47087647 | 0.0004964 |
| Scara5 | 2.74897112 | 0 | 1.46108161 | 0.00378887 |
| Zfp366 | 3.92765845 | 0.20651207 | 1.4166771 | 0.00500694 |
| D430019H16Rik | 11.9164161 | 2.81065426 | 1.39199841 | 0.0060801 |
| Inhbb | 5.51374715 | 0.31734854 | 1.37758121 | 0.00432629 |
| Smc1b | 2.76091441 | 0.20651207 | 1.37059076 | 0.00628462 |
| Tmem184a | 523.334056 | 177.757942 | 1.36159479 | 0.00478474 |
| Cav1 | 99.2316208 | 34.4244982 | 1.3485142 | 1.0121E-06 |
| Ogn | 3.98525579 | 0.63693476 | 1.32659682 | 0.00854978 |
| Kirrel2 | 4.06673969 | 0.63469708 | 1.3253283 | 0.00902595 |
| Olfml2a | 6.85926392 | 0.63693476 | 1.324215 | 0.0058919 |
| Aldh3a1 | 17.0427687 | 4.29551122 | 1.31459819 | 0.00631698 |
| Rasgrp1 | 18.4715301 | 5.37802954 | 1.30130576 | 0.00095563 |
| Adam12 | 9.96225912 | 2.52810281 | 1.28230408 | 0.00280925 |
| Rnf122 | 3.5876786 | 0 | 1.27322851 | 0.00886229 |
| Arsj | 5.41645834 | 1.36954513 | 1.27042589 | 0.00829008 |
| Hes1 | 34.725429 | 10.7605344 | 1.26884951 | 0.00169301 |
| Lrig1 | 302.696028 | 120.504426 | 1.26435942 | 0.00013759 |
| Paqr6 | 330.220748 | 111.164249 | 1.2494097 | 0.00068274 |
| Krt13 | 9.72378813 | 2.82805281 | 1.24398217 | 0.00788387 |
| Gm7862 | 4.5061093 | 1.0499589 | 1.20735209 | 0.01413077 |
| Thsd7b | 8.16719014 | 3.17398831 | 1.188397 | 0.01627807 |
| Gm7117 | 2.28185332 | 0.20651207 | 1.18104537 | 0.01942679 |
| Trabd2b | 10.3201448 | 2.94560233 | 1.17891103 | 0.00498456 |
| Liph | 6.42023457 | 1.5760572 | 1.14773557 | 0.0096683 |
| Ocstamp | 9.68021686 | 1.48485696 | 1.14460355 | 0.01393286 |
| Col1a1 | 212.509144 | 83.1446155 | 1.14318965 | 0.00017221 |
| Hsd11b2 | 9.64052537 | 1.68913135 | 1.13934796 | 0.01966345 |
| Cxcl1 | 52.8355339 | 17.9231031 | 1.13893915 | 0.00604384 |
| Aqp1 | 6599.5668 | 2760.00252 | 1.13852392 | 0.00019064 |
| Col6a1 | 427.854573 | 162.851524 | 1.13060137 | 0.00183668 |
| Zfp534 | 3.34322688 | 0.52609829 | 1.12503156 | 0.02681894 |
| Col18a1 | 196.442193 | 76.9651522 | 1.11979502 | 0.00075013 |
| Cdh11 | 30.1888083 | 11.0644569 | 1.11254252 | 0.00421008 |
| Eva1c | 6.57336003 | 3.05867648 | 1.11163945 | 0.0243206 |
| Col5a2 | 131.12385 | 50.2822455 | 1.10694299 | 0.00212313 |
| Vill | 22.4887359 | 9.07364078 | 1.10189258 | 0.00238284 |
| Gpr37 | 3.0928126 | 1.16527074 | 1.06684285 | 0.03807821 |
| Cd207 | 3.88972765 | 0 | 1.06437925 | 0.03457128 |
| AC153987.1 | 8.01616562 | 3.77165061 | 1.05256569 | 0.02677349 |
| Rph3al | 381.126067 | 174.121345 | 1.05113146 | 0.00035962 |
| Clec4f | 63.6568838 | 5.79278844 | 1.04944643 | 0.03336474 |
| Atoh8 | 23.7506678 | 10.2125622 | 1.04432404 | 0.00215699 |
| 1700024P16Rik | 3.65545854 | 0.63469708 | 1.03712082 | 0.04091674 |
| Tmem100 | 1.38941013 | 0 | 1.03140092 | 0.0279601 |
| Col6a2 | 273.873633 | 106.520053 | 1.03035388 | 0.00835309 |
| Asb2 | 10.0479449 | 3.55842549 | 1.02730353 | 0.02939609 |
| Fam167a | 6.65484394 | 1.57381951 | 1.01767686 | 0.02427301 |
| A930017K11Rik | 5.62121857 | 2.73909025 | 1.01688461 | 0.03623603 |
| Lilra5 | 6.55545418 | 3.27811173 | 1.01307054 | 0.04494724 |
| Mmp23 | 16.6627571 | 7.27591064 | 1.00985778 | 0.00493012 |
| Dcaf12l1 | 8.63254726 | 2.93044146 | 1.00641356 | 0.03657599 |
| Nrarp | 94.972046 | 45.9238531 | 1.00466295 | 0.00014579 |
| Abcc9 | 2.43287784 | 0 | 1.00167663 | 0.04616703 |
| Hmgb1-ps3 | 10.1610386 | 4.10863538 | 1.00164038 | 0.01727098 |
| Gas1 | 44.3357832 | 17.6119646 | 0.99474603 | 0.01839658 |
| Slc28a1 | 1.51058552 | 0 | 0.98215517 | 0.01784198 |
| Wisp1 | 35.360833 | 13.3430706 | 0.98044872 | 0.02237125 |
| Col12a1 | 29.5351945 | 9.98244148 | 0.97946423 | 0.01751053 |
| Slc12a3 | 1.00377623 | 0 | 0.97537886 | 0.02235874 |
| Dnase1l3 | 11.18096 | 1.5760572 | 0.97491358 | 0.02894645 |
| Cyp4f39 | 11.89395 | 6.75876308 | 0.96872813 | 0.04542702 |
| Snta1 | 14.4163934 | 6.08876599 | 0.96708309 | 0.015174 |
| Tspan15 | 22.8269369 | 9.98641393 | 0.96591205 | 0.0119584 |
| Esm1 | 5.50004318 | 2.00647989 | 0.95814321 | 0.04541969 |
| Cd247 | 15.3186608 | 7.61065773 | 0.94813025 | 0.02951023 |
| Gm6257 | 4.27360087 | 1.0499589 | 0.94591691 | 0.04788675 |
| S1pr3 | 54.5558577 | 20.8816286 | 0.94232183 | 0.01888015 |
| Cacng7 | 9.01818116 | 4.00848441 | 0.93917819 | 0.02258948 |
| Gm5939 | 1.42910161 | 0.31958622 | 0.93577073 | 0.04553372 |
| Tpbg | 17.1976548 | 7.70633333 | 0.93287743 | 0.01857993 |
| AC144852.1 | 6.62675549 | 2.53481587 | 0.93211973 | 0.04333174 |
| Ntn1 | 19.0780694 | 7.97769635 | 0.93047436 | 0.03862783 |
| Actg2 | 9.67601498 | 3.24555232 | 0.92701479 | 0.02305384 |
| Tlx2 | 1.34761771 | 0.31958622 | 0.92268665 | 0.04879221 |
| Sspn | 8.35614547 | 3.16951294 | 0.92175661 | 0.04124776 |
| Spa17 | 9.39751224 | 3.78681147 | 0.91601136 | 0.04512782 |
| Bend6 | 11.133205 | 4.53905807 | 0.90872316 | 0.03076154 |
| Foxd2 | 1.45895075 | 0.20651207 | 0.8954676 | 0.04312438 |
| B4galnt3 | 10.9460104 | 6.43246381 | 0.89432984 | 0.04033596 |
| GM12302 | 1.31986951 | 0 | 0.89269413 | 0.04482166 |
| Col16a1 | 43.5483702 | 18.6663989 | 0.89062732 | 0.03076839 |
| Gm15731 | 1.48283732 | 0 | 0.889261 | 0.04479118 |
| Fstl1 | 275.018872 | 125.259473 | 0.87856039 | 0.01895808 |
| Ssc5d | 10.3219055 | 4.01295978 | 0.86708132 | 0.04160147 |
| 9030025P20Rik | 27.6962321 | 13.4322682 | 0.86410103 | 0.00443528 |
| Gm12418 | 8.53701913 | 3.67597501 | 0.86390743 | 0.04961127 |
| Aif1 | 54.7992111 | 23.4638968 | 0.84868591 | 0.02487323 |
| Ddit4l | 11.9339817 | 5.47146746 | 0.84776617 | 0.0341641 |
| 4930502E18Rik | 1.07331685 | 0 | 0.84104439 | 0.03341233 |
| 2610027K06Rik | 59.7626735 | 35.8991726 | 0.84061643 | 0.00465132 |
| Dclk2 | 288.191864 | 208.459032 | 0.8381586 | 0.04539156 |
| Gpc6 | 11.6596809 | 5.15411892 | 0.83037105 | 0.03466637 |
| Ttc39d | 0.81095928 | 0 | 0.82404948 | 0.04066625 |
| Gm12664 | 0.81095928 | 0 | 0.82404948 | 0.04066625 |
| Epb4.1l3 | 70.3293115 | 33.1792156 | 0.82169186 | 0.04231606 |
| Angptl2 | 107.563558 | 56.1773897 | 0.81984491 | 0.00512081 |
| Jag1 | 29.4452887 | 15.0779196 | 0.81066807 | 0.00616268 |
| Loxl1 | 16.5433606 | 7.6973826 | 0.80708755 | 0.04502712 |
| Fgf13 | 31.1806594 | 17.4191466 | 0.80689698 | 0.02150124 |
| Zfp532 | 12.7807527 | 5.46922977 | 0.80676916 | 0.04988182 |
| Nkapl | 11.53044197 | 5.980167202 | 0.806698222 | 0.039221133 |
