# Supplementary material for: IKAROS is required for the measured response of NOTCH target genes upon external NOTCH signaling
Source: PLoS Genet. 2021 Mar 26;17(3):e1009478. doi: 10.1371/journal.pgen.1009478 (PMC8026084; doi:10.1371/journal.pgen.1009478)
Supplement: S4 Table — (DOCX) [file pgen.1009478.s004.docx]

**Table S4.** Principal functional annotations of genes listed in Table S3. Annotations with a minimum of 5 genes are presented.

| **Term** | Count | P-value |
| --- | --- | --- |
| **cell surface receptor signaling pathway** | 30 | 3,10E-06 |
| **response to organic substance** | 29 | 1,00E-03 |
| **regulation of multicellular organismal development** | 27 | 2,90E-06 |
| **regulation of signal transduction** | 27 | 3,00E-04 |
| **regulation of cell communication** | 27 | 1,90E-03 |
| **regulation of signaling** | 27 | 2,10E-03 |
| **cellular response to chemical stimulus** | 26 | 1,60E-03 |
| **cell adhesion** | 24 | 5,20E-06 |
| **biological adhesion** | 24 | 5,90E-06 |
| **cell development** | 23 | 3,00E-03 |
| **response to external stimulus** | 23 | 3,40E-03 |
| **regulation of cell differentiation** | 22 | 3,50E-04 |
| **cellular response to organic substance** | 22 | 2,60E-03 |
| **movement of cell or subcellular component** | 21 | 5,30E-04 |
| **nervous system development** | 21 | 1,30E-02 |
| **positive regulation of multicellular organismal process** | 20 | 5,50E-04 |
| **regulation of cell proliferation** | 20 | 7,80E-04 |
| **response to oxygen-containing compound** | 20 | 9,50E-04 |
| **positive regulation of response to stimulus** | 20 | 2,80E-03 |
| **regulation of cellular component organization** | 20 | 4,20E-02 |
| **regulation of immune system process** | 19 | 7,50E-05 |
| **locomotion** | 19 | 9,00E-04 |
| **response to endogenous stimulus** | 19 | 1,60E-03 |
| **cell proliferation** | 19 | 8,10E-03 |
| **cell migration** | 17 | 4,70E-04 |
| **positive regulation of developmental process** | 17 | 7,60E-04 |
| **positive regulation of signal transduction** | 17 | 1,40E-03 |
| **cell motility** | 17 | 1,60E-03 |
| **localization of cell** | 17 | 1,60E-03 |
| **positive regulation of cell communication** | 17 | 4,50E-03 |
| **positive regulation of signaling** | 17 | 4,70E-03 |
| **cell death** | 17 | 5,70E-02 |
| **positive regulation of cell proliferation** | 16 | 1,10E-04 |
| **organ morphogenesis** | 16 | 3,20E-04 |
| **negative regulation of multicellular organismal process** | 16 | 9,80E-04 |
| **embryo development** | 16 | 1,00E-03 |
| **anatomical structure formation involved in morphogenesis** | 16 | 2,30E-03 |
| **neurogenesis** | 16 | 2,30E-02 |
| **response to lipid** | 15 | 6,40E-04 |
| **cardiovascular system development** | 15 | 1,20E-03 |
| **circulatory system development** | 15 | 1,20E-03 |
| **cellular response to endogenous stimulus** | 15 | 3,50E-03 |
| **negative regulation of response to stimulus** | 15 | 1,10E-02 |
| **protein complex subunit organization** | 15 | 1,50E-02 |
| **generation of neurons** | 15 | 2,80E-02 |
| **regulation of transport** | 15 | 9,50E-02 |
| **negative regulation of developmental process** | 14 | 4,60E-04 |
| **regulation of cell development** | 14 | 2,20E-03 |
| **immune response** | 14 | 1,20E-02 |
| **neuron differentiation** | 14 | 2,80E-02 |
| **proteolysis** | 14 | 6,30E-02 |
| **blood vessel development** | 13 | 1,30E-04 |
| **vasculature development** | 13 | 2,20E-04 |
| **negative regulation of cell differentiation** | 13 | 7,80E-04 |
| **regulation of cellular component movement** | 13 | 1,50E-03 |
| **positive regulation of cell differentiation** | 13 | 5,20E-03 |
| **epithelium development** | 13 | 1,80E-02 |
| **negative regulation of cell communication** | 13 | 1,80E-02 |
| **negative regulation of signaling** | 13 | 1,80E-02 |
| **sensory organ development** | 12 | 3,40E-04 |
| **tube development** | 12 | 1,10E-03 |
| **regulation of cell migration** | 12 | 1,70E-03 |
| **regulation of cell motility** | 12 | 2,60E-03 |
| **regulation of locomotion** | 12 | 3,70E-03 |
| **enzyme linked receptor protein signaling pathway** | 12 | 3,90E-03 |
| **response to organonitrogen compound** | 12 | 6,20E-03 |
| **hematopoietic or lymphoid organ development** | 12 | 1,00E-02 |
| **immune system development** | 12 | 1,40E-02 |
| **response to nitrogen compound** | 12 | 1,60E-02 |
| **negative regulation of signal transduction** | 12 | 1,80E-02 |
| **regulation of anatomical structure morphogenesis** | 12 | 1,90E-02 |
| **response to abiotic stimulus** | 12 | 3,80E-02 |
| **positive regulation of cellular component organization** | 12 | 6,30E-02 |
| **protein complex assembly** | 12 | 7,10E-02 |
| **protein complex biogenesis** | 12 | 7,10E-02 |
| **blood vessel morphogenesis** | 11 | 5,30E-04 |
| **embryonic morphogenesis** | 11 | 2,10E-03 |
| **hemopoiesis** | 11 | 1,80E-02 |
| **regulation of nervous system development** | 11 | 2,30E-02 |
| **response to organic cyclic compound** | 11 | 3,80E-02 |
| **cellular response to oxygen-containing compound** | 11 | 5,20E-02 |
| **negative regulation of cell development** | 10 | 1,20E-04 |
| **regulation of hemopoiesis** | 10 | 1,60E-04 |
| **cellular response to growth factor stimulus** | 10 | 4,20E-03 |
| **response to growth factor** | 10 | 5,40E-03 |
| **tissue morphogenesis** | 10 | 6,90E-03 |
| **positive regulation of immune system process** | 10 | 2,20E-02 |
| **metal ion transport** | 10 | 2,20E-02 |
| **cell morphogenesis involved in differentiation** | 10 | 2,50E-02 |
| **response to hormone** | 10 | 2,90E-02 |
| **regulation of neurogenesis** | 10 | 2,90E-02 |
| **cation transport** | 10 | 4,90E-02 |
| **growth** | 10 | 9,30E-02 |
| **Notch signaling pathway** | 9 | 3,20E-06 |
| **response to steroid hormone** | 9 | 7,30E-04 |
| **response to acid chemical** | 9 | 1,00E-03 |
| **negative regulation of immune system process** | 9 | 1,30E-03 |
| **positive regulation of cell motility** | 9 | 2,90E-03 |
| **positive regulation of cellular component movement** | 9 | 3,40E-03 |
| **positive regulation of locomotion** | 9 | 3,50E-03 |
| **cellular response to organonitrogen compound** | 9 | 6,30E-03 |
| **chemotaxis** | 9 | 7,20E-03 |
| **taxis** | 9 | 7,30E-03 |
| **cellular response to nitrogen compound** | 9 | 1,40E-02 |
| **regulation of cell adhesion** | 9 | 1,90E-02 |
| **regulation of neuron differentiation** | 9 | 2,70E-02 |
| **single organismal cell-cell adhesion** | 9 | 4,00E-02 |
| **immune effector process** | 9 | 4,50E-02 |
| **head development** | 9 | 5,60E-02 |
| **single organism cell adhesion** | 9 | 5,80E-02 |
| **positive regulation of protein phosphorylation** | 9 | 9,70E-02 |
| **sensory organ morphogenesis** | 8 | 9,40E-04 |
| **negative regulation of neurogenesis** | 8 | 9,80E-04 |
| **negative regulation of nervous system development** | 8 | 1,50E-03 |
| **transmembrane receptor protein serine/threonine kinase signaling pathway** | 8 | 2,00E-03 |
| **angiogenesis** | 8 | 8,80E-03 |
| **positive regulation of cell migration** | 8 | 9,00E-03 |
| **response to drug** | 8 | 9,10E-03 |
| **response to wounding** | 8 | 1,90E-02 |
| **heart development** | 8 | 3,90E-02 |
| **epithelial cell differentiation** | 8 | 4,90E-02 |
| **response to cytokine** | 8 | 6,40E-02 |
| **lymphocyte activation** | 8 | 6,80E-02 |
| **chordate embryonic development** | 8 | 8,90E-02 |
| **embryo development ending in birth or egg hatching** | 8 | 9,30E-02 |
| **brain development** | 8 | 9,70E-02 |
| **extracellular matrix organization** | 7 | 1,30E-03 |
| **extracellular structure organization** | 7 | 1,30E-03 |
| **negative regulation of neuron differentiation** | 7 | 1,30E-03 |
| **ear development** | 7 | 1,70E-03 |
| **cellular response to acid chemical** | 7 | 2,10E-03 |
| **cell chemotaxis** | 7 | 2,50E-03 |
| **eye development** | 7 | 2,00E-02 |
| **wound healing** | 7 | 2,00E-02 |
| **response to extracellular stimulus** | 7 | 3,50E-02 |
| **skeletal system development** | 7 | 4,70E-02 |
| **regulation of system process** | 7 | 4,90E-02 |
| **leukocyte cell-cell adhesion** | 7 | 5,10E-02 |
| **regulation of cell activation** | 7 | 5,40E-02 |
| **cellular response to organic cyclic compound** | 7 | 5,60E-02 |
| **protein oligomerization** | 7 | 5,80E-02 |
| **cell morphogenesis involved in neuron differentiation** | 7 | 6,00E-02 |
| **cation transmembrane transport** | 7 | 6,10E-02 |
| **cellular response to cytokine stimulus** | 7 | 6,60E-02 |
| **leukocyte differentiation** | 7 | 6,60E-02 |
| **regulation of cytokine production** | 7 | 7,60E-02 |
| **regulation of Notch signaling pathway** | 6 | 5,20E-05 |
| **regulation of epithelial cell differentiation** | 6 | 7,00E-04 |
| **regulation of myeloid cell differentiation** | 6 | 4,80E-03 |
| **inner ear development** | 6 | 5,20E-03 |
| **mesenchyme development** | 6 | 9,60E-03 |
| **protein maturation** | 6 | 1,30E-02 |
| **regulation of leukocyte differentiation** | 6 | 1,40E-02 |
| **regulation of leukocyte cell-cell adhesion** | 6 | 2,00E-02 |
| **kidney development** | 6 | 2,10E-02 |
| **leukocyte migration** | 6 | 2,20E-02 |
| **renal system development** | 6 | 2,70E-02 |
| **urogenital system development** | 6 | 4,30E-02 |
| **positive regulation of cell adhesion** | 6 | 4,40E-02 |
| **response to oxygen levels** | 6 | 4,60E-02 |
| **regulation of cell-cell adhesion** | 6 | 5,00E-02 |
| **myeloid cell differentiation** | 6 | 5,30E-02 |
| **Wnt signaling pathway** | 6 | 5,40E-02 |
| **cell-cell signaling by wnt** | 6 | 5,50E-02 |
| **regulation of cell growth** | 6 | 6,50E-02 |
| **regulation of lymphocyte activation** | 6 | 6,60E-02 |
| **tube morphogenesis** | 6 | 6,60E-02 |
| **monovalent inorganic cation transport** | 6 | 7,10E-02 |
| **response to nutrient levels** | 6 | 7,60E-02 |
| **muscle tissue development** | 6 | 8,10E-02 |
| **T cell aggregation** | 6 | 9,70E-02 |
| **T cell activation** | 6 | 9,70E-02 |
| **cell surface receptor signaling pathway involved in cell-cell signaling** | 6 | 9,80E-02 |
| **lymphocyte aggregation** | 6 | 9,80E-02 |
| **aorta development** | 5 | 2,80E-04 |
| **artery development** | 5 | 1,60E-03 |
| **endothelial cell differentiation** | 5 | 1,70E-03 |
| **response to estrogen** | 5 | 2,80E-03 |
| **endothelium development** | 5 | 3,10E-03 |
| **formation of primary germ layer** | 5 | 3,70E-03 |
| **negative regulation of hemopoiesis** | 5 | 6,70E-03 |
| **cellular response to amino acid stimulus** | 5 | 7,00E-03 |
| **nephron development** | 5 | 8,40E-03 |
| **regulation of leukocyte migration** | 5 | 1,30E-02 |
| **eye morphogenesis** | 5 | 1,30E-02 |
| **response to corticosteroid** | 5 | 1,50E-02 |
| **gastrulation** | 5 | 1,60E-02 |
| **response to amino acid** | 5 | 1,70E-02 |
| **positive regulation of hemopoiesis** | 5 | 1,80E-02 |
| **leukocyte chemotaxis** | 5 | 2,00E-02 |
| **regulation of chemotaxis** | 5 | 2,10E-02 |
| **cellular response to transforming growth factor beta stimulus** | 5 | 2,20E-02 |
| **response to transforming growth factor beta** | 5 | 2,30E-02 |
| **regulation of transmembrane receptor protein serine/threonine kinase signaling pathway** | 5 | 2,50E-02 |
| **protein processing** | 5 | 3,20E-02 |
| **response to mechanical stimulus** | 5 | 3,40E-02 |
| **regulation of cellular response to growth factor stimulus** | 5 | 3,70E-02 |
| **regulation of Wnt signaling pathway** | 5 | 4,00E-02 |
| **telencephalon development** | 5 | 5,20E-02 |
| **cellular response to external stimulus** | 5 | 5,70E-02 |
| **skin development** | 5 | 5,80E-02 |
| **regulation of T cell activation** | 5 | 6,00E-02 |
| **cell-substrate adhesion** | 5 | 7,10E-02 |
| **Ras protein signal transduction** | 5 | 7,50E-02 |
| **positive regulation of cell activation** | 5 | 8,00E-02 |
| **embryonic organ morphogenesis** | 5 | 8,90E-02 |
| **regulation of epithelial cell proliferation** | 5 | 9,00E-02 |
| **leukocyte mediated immunity** | 5 | 9,90E-02 |
