# Supplementary material for: IKAROS is required for the measured response of NOTCH target genes upon external NOTCH signaling
Source: PLoS Genet. 2021 Mar 26;17(3):e1009478. doi: 10.1371/journal.pgen.1009478 (PMC8026084; doi:10.1371/journal.pgen.1009478)
Supplement: S5 Table — (DOCX) [file pgen.1009478.s005.docx]

**Table S5.** Principal functional annotations of the 499 genes specifically overexpressed in Ik^Null^/OP9 cells; log2 ≥ 0.08; *p* < 0.05. Annotations with a minimum of 20 genes are presented (see Fig 1B).

| **Term** | Count | P-value |
| --- | --- | --- |
| **regulation of cell communication** | 71 | 1,60E-03 |
| **regulation of signaling** | 71 | 2,00E-03 |
| **response to organic substance** | 69 | 1,70E-02 |
| **regulation of signal transduction** | 66 | 7,40E-04 |
| **cellular response to chemical stimulus** | 66 | 2,90E-03 |
| **phosphorus metabolic process** | 62 | 8,20E-02 |
| **cell surface receptor signaling pathway** | 61 | 1,20E-03 |
| **regulation of cell differentiation** | 55 | 1,00E-04 |
| **nervous system development** | 54 | 2,30E-02 |
| **regulation of multicellular organismal development** | 53 | 1,00E-03 |
| **cellular response to organic substance** | 53 | 9,40E-03 |
| **cell development** | 53 | 3,70E-02 |
| **regulation of molecular function** | 50 | 6,10E-02 |
| **regulation of transport** | 48 | 6,90E-03 |
| **positive regulation of response to stimulus** | 47 | 1,10E-02 |
| **negative regulation of response to stimulus** | 45 | 7,60E-05 |
| **negative regulation of multicellular organismal process** | 44 | 4,00E-06 |
| **ion transport** | 41 | 1,20E-03 |
| **neurogenesis** | 41 | 2,60E-02 |
| **homeostatic process** | 41 | 4,60E-02 |
| **cell adhesion** | 40 | 2,50E-02 |
| **biological adhesion** | 40 | 2,80E-02 |
| **response to endogenous stimulus** | 40 | 3,10E-02 |
| **positive regulation of cell communication** | 39 | 1,70E-02 |
| **positive regulation of signaling** | 39 | 1,80E-02 |
| **regulation of phosphate metabolic process** | 39 | 5,40E-02 |
| **regulation of phosphorus metabolic process** | 39 | 5,50E-02 |
| **regulation of catalytic activity** | 39 | 6,60E-02 |
| **response to oxygen-containing compound** | 39 | 7,20E-02 |
| **generation of neurons** | 38 | 3,50E-02 |
| **positive regulation of signal transduction** | 37 | 8,10E-03 |
| **defense response** | 37 | 3,60E-02 |
| **regulation of intracellular signal transduction** | 37 | 6,40E-02 |
| **anatomical structure formation involved in morphogenesis** | 36 | 6,50E-03 |
| **locomotion** | 36 | 7,70E-02 |
| **neuron differentiation** | 35 | 3,40E-02 |
| **negative regulation of cell communication** | 34 | 5,60E-03 |
| **negative regulation of signaling** | 34 | 5,90E-03 |
| **cellular component morphogenesis** | 34 | 5,40E-02 |
| **cell projection organization** | 34 | 5,80E-02 |
| **regulation of phosphorylation** | 34 | 6,60E-02 |
| **regulation of response to stress** | 33 | 1,40E-02 |
| **lipid metabolic process** | 33 | 1,80E-02 |
| **regulation of immune system process** | 33 | 2,50E-02 |
| **localization of cell** | 33 | 6,00E-02 |
| **cell motility** | 33 | 6,00E-02 |
| **negative regulation of developmental process** | 32 | 1,30E-04 |
| **response to abiotic stimulus** | 32 | 1,40E-02 |
| **cellular response to endogenous stimulus** | 32 | 2,20E-02 |
| **cell morphogenesis** | 32 | 5,30E-02 |
| **negative regulation of cell differentiation** | 31 | 7,10E-05 |
| **epithelium development** | 31 | 2,60E-02 |
| **immune response** | 31 | 5,20E-02 |
| **regulation of nervous system development** | 30 | 2,30E-03 |
| **organ morphogenesis** | 30 | 1,20E-02 |
| **chemical homeostasis** | 30 | 2,40E-02 |
| **cell migration** | 30 | 6,00E-02 |
| **regulation of hydrolase activity** | 29 | 3,00E-03 |
| **cation transport** | 29 | 3,60E-03 |
| **positive regulation of transport** | 29 | 1,30E-02 |
| **regulation of cell development** | 29 | 1,40E-02 |
| **negative regulation of signal transduction** | 29 | 1,70E-02 |
| **embryo development** | 29 | 6,60E-02 |
| **metal ion transport** | 28 | 9,30E-04 |
| **regulation of anatomical structure morphogenesis** | 28 | 3,10E-02 |
| **cellular response to oxygen-containing compound** | 28 | 4,20E-02 |
| **regulation of neurogenesis** | 27 | 3,60E-03 |
| **cardiovascular system development** | 27 | 6,40E-02 |
| **circulatory system development** | 27 | 6,40E-02 |
| **neuron development** | 27 | 8,30E-02 |
| **regulation of cellular component movement** | 26 | 9,80E-03 |
| **negative regulation of molecular function** | 26 | 2,50E-02 |
| **positive regulation of cell differentiation** | 26 | 4,70E-02 |
| **positive regulation of phosphate metabolic process** | 26 | 8,70E-02 |
| **positive regulation of phosphorus metabolic process** | 26 | 8,70E-02 |
| **regulation of locomotion** | 25 | 1,20E-02 |
| **neuron projection development** | 25 | 4,10E-02 |
| **organophosphate metabolic process** | 25 | 6,10E-02 |
| **response to lipid** | 25 | 8,00E-02 |
| **response to organic cyclic compound** | 25 | 9,10E-02 |
| **regulation of neuron differentiation** | 24 | 2,70E-03 |
| **inorganic ion homeostasis** | 24 | 2,90E-03 |
| **ion homeostasis** | 24 | 5,50E-03 |
| **regulation of response to external stimulus** | 24 | 1,10E-02 |
| **regulation of cell motility** | 24 | 1,40E-02 |
| **cation homeostasis** | 23 | 4,60E-03 |
| **regulation of cell migration** | 23 | 1,40E-02 |
| **cell morphogenesis involved in differentiation** | 23 | 3,10E-02 |
| **enzyme linked receptor protein signaling pathway** | 23 | 3,90E-02 |
| **intracellular protein transport** | 23 | 8,60E-02 |
| **positive regulation of intracellular signal transduction** | 23 | 8,90E-02 |
| **regulation of ion transport** | 22 | 1,90E-03 |
| **cellular chemical homeostasis** | 22 | 3,30E-02 |
| **response to hormone** | 22 | 6,40E-02 |
| **cellular homeostasis** | 22 | 9,40E-02 |
| **blood vessel morphogenesis** | 21 | 1,80E-03 |
| **cellular ion homeostasis** | 21 | 6,00E-03 |
| **blood vessel development** | 21 | 1,10E-02 |
| **vasculature development** | 21 | 2,00E-02 |
| **single-organism catabolic process** | 21 | 3,30E-02 |
| **inflammatory response** | 20 | 1,50E-02 |
| **positive regulation of cell death** | 20 | 1,60E-02 |
| **epithelial cell differentiation** | 20 | 2,00E-02 |
| **negative regulation of catalytic activity** | 20 | 2,80E-02 |
| **response to cytokine** | 20 | 3,40E-02 |
