# Supplementary material for: IKAROS is required for the measured response of NOTCH target genes upon external NOTCH signaling
Source: PLoS Genet. 2021 Mar 26;17(3):e1009478. doi: 10.1371/journal.pgen.1009478 (PMC8026084; doi:10.1371/journal.pgen.1009478)
Supplement: S6 Table — (DOCX) [file pgen.1009478.s006.docx]

**Table S6.** Principal functional annotations of the 844 genes specifically overexpressed in Ik^Null^/OP9-DL1 *vs.* Ik^WT^/OP9-DL1 cells; log2 ≥ 0.08; *p* < 0.05. Annotations with a minimum of 20 genes are presented (see Fig 1B).

| **Term** | Count | P-value |
| --- | --- | --- |
| **positive regulation of metabolic process** | 128 | 5,10E-05 |
| **cellular protein modification process** | 126 | 2,40E-02 |
| **protein modification process** | 126 | 2,40E-02 |
| **regulation of signaling** | 124 | 1,40E-05 |
| **regulation of cell communication** | 123 | 1,50E-05 |
| **phosphate-containing compound metabolic process** | 123 | 6,00E-05 |
| **phosphorus metabolic process** | 123 | 6,30E-05 |
| **positive regulation of macromolecule metabolic process** | 120 | 6,90E-05 |
| **positive regulation of cellular metabolic process** | 116 | 2,70E-04 |
| **regulation of signal transduction** | 115 | 2,80E-06 |
| **response to organic substance** | 115 | 4,80E-03 |
| **intracellular signal transduction** | 113 | 1,40E-06 |
| **cellular response to chemical stimulus** | 109 | 4,80E-04 |
| **regulation of molecular function** | 108 | 5,40E-07 |
| **response to external stimulus** | 104 | 3,50E-05 |
| **regulation of cellular component organization** | 102 | 4,30E-04 |
| **cell development** | 99 | 2,50E-04 |
| **regulation of protein metabolic process** | 99 | 4,00E-03 |
| **cell proliferation** | 93 | 2,70E-06 |
| **phosphorylation** | 93 | 1,10E-04 |
| **regulation of catalytic activity** | 92 | 2,50E-08 |
| **negative regulation of metabolic process** | 92 | 7,50E-02 |
| **regulation of cellular protein metabolic process** | 91 | 7,50E-03 |
| **regulation of multicellular organismal development** | 90 | 2,00E-05 |
| **cellular response to organic substance** | 89 | 1,30E-03 |
| **regulation of cell proliferation** | 88 | 2,30E-07 |
| **movement of cell or subcellular component** | 88 | 1,80E-06 |
| **cell surface receptor signaling pathway** | 87 | 2,10E-02 |
| **positive regulation of response to stimulus** | 86 | 5,80E-05 |
| **negative regulation of cellular metabolic process** | 85 | 8,60E-02 |
| **nervous system development** | 83 | 5,40E-02 |
| **locomotion** | 82 | 6,40E-07 |
| **protein phosphorylation** | 82 | 7,30E-05 |
| **regulation of intracellular signal transduction** | 80 | 4,20E-06 |
| **positive regulation of multicellular organismal process** | 80 | 1,20E-05 |
| **regulation of phosphate metabolic process** | 79 | 4,50E-05 |
| **regulation of phosphorus metabolic process** | 79 | 4,60E-05 |
| **regulation of cell differentiation** | 77 | 2,30E-03 |
| **defense response** | 76 | 9,10E-06 |
| **cell death** | 76 | 3,70E-02 |
| **cell adhesion** | 75 | 1,40E-04 |
| **biological adhesion** | 75 | 1,80E-04 |
| **localization of cell** | 74 | 1,10E-06 |
| **cell motility** | 74 | 1,10E-06 |
| **positive regulation of cellular biosynthetic process** | 74 | 2,00E-03 |
| **positive regulation of biosynthetic process** | 74 | 3,40E-03 |
| **programmed cell death** | 74 | 1,70E-02 |
| **response to oxygen-containing compound** | 73 | 1,70E-03 |
| **regulation of immune system process** | 71 | 5,50E-07 |
| **positive regulation of protein metabolic process** | 70 | 9,20E-05 |
| **positive regulation of nitrogen compound metabolic process** | 70 | 1,50E-02 |
| **regulation of transcription from RNA polymerase II promoter** | 70 | 2,10E-02 |
| **apoptotic process** | 70 | 2,20E-02 |
| **cell migration** | 69 | 5,30E-07 |
| **immune response** | 69 | 1,40E-06 |
| **regulation of phosphorylation** | 69 | 1,10E-04 |
| **positive regulation of gene expression** | 69 | 1,40E-02 |
| **single-organism organelle organization** | 69 | 1,50E-02 |
| **regulation of protein modification process** | 68 | 6,30E-03 |
| **regulation of transport** | 68 | 6,60E-02 |
| **regulation of cell death** | 67 | 9,50E-03 |
| **negative regulation of multicellular organismal process** | 66 | 1,40E-06 |
| **positive regulation of signaling** | 66 | 2,50E-03 |
| **regulation of programmed cell death** | 66 | 2,50E-03 |
| **regulation of protein phosphorylation** | 65 | 1,20E-04 |
| **positive regulation of cell communication** | 65 | 3,40E-03 |
| **transcription from RNA polymerase II promoter** | 65 | 4,60E-02 |
| **regulation of apoptotic process** | 64 | 4,60E-03 |
| **positive regulation of macromolecule biosynthetic process** | 64 | 1,70E-02 |
| **response to endogenous stimulus** | 64 | 2,40E-02 |
| **cellular component morphogenesis** | 63 | 1,40E-03 |
| **homeostatic process** | 63 | 8,60E-02 |
| **positive regulation of nucleobase-containing compound metabolic process** | 62 | 6,10E-02 |
| **positive regulation of molecular function** | 61 | 4,90E-04 |
| **positive regulation of signal transduction** | 60 | 2,30E-03 |
| **positive regulation of cellular protein metabolic process** | 60 | 2,60E-03 |
| **neurogenesis** | 60 | 1,00E-01 |
| **positive regulation of developmental process** | 59 | 9,80E-04 |
| **cell morphogenesis** | 59 | 1,70E-03 |
| **proteolysis** | 59 | 6,50E-02 |
| **positive regulation of cellular component organization** | 58 | 1,60E-03 |
| **regulation of cellular component movement** | 56 | 1,10E-07 |
| **cell activation** | 56 | 1,80E-06 |
| **negative regulation of response to stimulus** | 56 | 1,70E-02 |
| **response to biotic stimulus** | 55 | 2,70E-04 |
| **cytoskeleton organization** | 55 | 5,90E-04 |
| **regulation of response to stress** | 55 | 2,30E-03 |
| **ion transport** | 55 | 2,60E-02 |
| **positive regulation of RNA metabolic process** | 55 | 4,30E-02 |
| **positive regulation of catalytic activity** | 54 | 2,60E-05 |
| **protein complex subunit organization** | 54 | 7,10E-02 |
| **negative regulation of molecular function** | 53 | 1,50E-05 |
| **response to other organism** | 53 | 2,40E-04 |
| **response to external biotic stimulus** | 53 | 2,50E-04 |
| **organ morphogenesis** | 53 | 3,10E-04 |
| **positive regulation of phosphorus metabolic process** | 53 | 4,20E-04 |
| **positive regulation of phosphate metabolic process** | 53 | 4,20E-04 |
| **circulatory system development** | 53 | 5,30E-04 |
| **cardiovascular system development** | 53 | 5,30E-04 |
| **anatomical structure formation involved in morphogenesis** | 53 | 1,70E-02 |
| **positive regulation of RNA biosynthetic process** | 53 | 4,40E-02 |
| **positive regulation of nucleic acid-templated transcription** | 52 | 5,80E-02 |
| **positive regulation of transcription, DNA-templated** | 52 | 5,80E-02 |
| **regulation of anatomical structure morphogenesis** | 51 | 8,70E-04 |
| **cell-cell adhesion** | 51 | 3,20E-03 |
| **embryo development** | 51 | 8,90E-03 |
| **regulation of hydrolase activity** | 50 | 5,80E-05 |
| **lipid metabolic process** | 50 | 2,90E-02 |
| **regulation of locomotion** | 49 | 1,20E-05 |
| **positive regulation of cell proliferation** | 49 | 3,70E-04 |
| **immune system development** | 49 | 9,30E-04 |
| **epithelium development** | 49 | 2,00E-02 |
| **immune effector process** | 48 | 3,10E-06 |
| **regulation of cell motility** | 48 | 7,80E-06 |
| **hematopoietic or lymphoid organ development** | 48 | 6,20E-04 |
| **regulation of organelle organization** | 48 | 2,50E-02 |
| **negative regulation of cell communication** | 48 | 2,90E-02 |
| **negative regulation of signaling** | 48 | 3,00E-02 |
| **regulation of cell migration** | 47 | 4,40E-06 |
| **positive regulation of immune system process** | 47 | 1,70E-05 |
| **positive regulation of cell differentiation** | 47 | 2,60E-03 |
| **regulation of cell development** | 47 | 4,10E-03 |
| **cellular response to oxygen-containing compound** | 47 | 1,10E-02 |
| **hemopoiesis** | 46 | 6,70E-04 |
| **positive regulation of phosphorylation** | 46 | 1,70E-03 |
| **negative regulation of programmed cell death** | 46 | 2,40E-03 |
| **growth** | 46 | 1,10E-02 |
| **negative regulation of cell death** | 46 | 1,20E-02 |
| **carbohydrate derivative metabolic process** | 46 | 2,10E-02 |
| **positive regulation of protein modification process** | 46 | 2,20E-02 |
| **actin filament-based process** | 45 | 9,20E-07 |
| **positive regulation of intracellular signal transduction** | 45 | 1,60E-03 |
| **negative regulation of signal transduction** | 45 | 1,40E-02 |
| **positive regulation of transcription from RNA polymerase II promoter** | 45 | 2,10E-02 |
| **regulation of transferase activity** | 44 | 1,10E-04 |
| **leukocyte activation** | 44 | 2,40E-04 |
| **cell morphogenesis involved in differentiation** | 44 | 2,60E-04 |
| **positive regulation of protein phosphorylation** | 44 | 2,20E-03 |
| **negative regulation of apoptotic process** | 44 | 5,20E-03 |
| **response to lipid** | 44 | 1,30E-02 |
| **regulation of response to external stimulus** | 43 | 2,00E-04 |
| **inflammatory response** | 42 | 2,40E-06 |
| **positive regulation of transport** | 42 | 3,60E-02 |
| **response to organic cyclic compound** | 42 | 3,60E-02 |
| **regulation of kinase activity** | 41 | 5,00E-05 |
| **single organism cell adhesion** | 41 | 1,40E-03 |
| **cellular lipid metabolic process** | 41 | 1,50E-02 |
| **cytokine production** | 40 | 1,50E-05 |
| **regulation of immune response** | 40 | 1,70E-05 |
| **negative regulation of catalytic activity** | 40 | 6,60E-05 |
| **negative regulation of protein metabolic process** | 40 | 8,20E-02 |
| **regulation of defense response** | 39 | 2,10E-05 |
| **negative regulation of developmental process** | 39 | 1,20E-02 |
| **actin cytoskeleton organization** | 38 | 8,90E-06 |
| **regulation of MAPK cascade** | 38 | 4,60E-04 |
| **single organismal cell-cell adhesion** | 38 | 2,20E-03 |
| **oxidation-reduction process** | 38 | 6,10E-02 |
| **organophosphate metabolic process** | 38 | 8,30E-02 |
| **regulation of cytokine production** | 37 | 2,70E-05 |
| **regulation of protein kinase activity** | 37 | 1,60E-04 |
| **innate immune response** | 37 | 3,60E-04 |
| **negative regulation of cell proliferation** | 37 | 4,30E-04 |
| **response to bacterium** | 37 | 8,10E-04 |
| **signal transduction by protein phosphorylation** | 37 | 1,50E-03 |
| **response to hormone** | 37 | 2,00E-02 |
| **cation transport** | 37 | 6,80E-02 |
| **regulation of nervous system development** | 37 | 7,80E-02 |
| **vasculature development** | 36 | 1,80E-03 |
| **MAPK cascade** | 36 | 2,40E-03 |
| **blood vessel development** | 35 | 1,30E-03 |
| **embryonic morphogenesis** | 35 | 1,60E-03 |
| **regulation of cellular component biogenesis** | 35 | 2,90E-02 |
| **enzyme linked receptor protein signaling pathway** | 35 | 4,60E-02 |
| **response to wounding** | 34 | 6,20E-05 |
| **leukocyte differentiation** | 34 | 1,50E-04 |
| **regulation of cell adhesion** | 34 | 2,40E-03 |
| **metal ion transport** | 34 | 4,10E-02 |
| **tube development** | 33 | 1,60E-02 |
| **single-organism catabolic process** | 33 | 2,10E-02 |
| **carbohydrate metabolic process** | 33 | 2,10E-02 |
| **regulation of neurogenesis** | 33 | 9,60E-02 |
| **chemotaxis** | 32 | 5,40E-04 |
| **taxis** | 32 | 5,60E-04 |
| **wound healing** | 31 | 8,20E-06 |
| **blood vessel morphogenesis** | 31 | 1,20E-03 |
| **positive regulation of apoptotic process** | 31 | 3,40E-03 |
| **positive regulation of programmed cell death** | 31 | 3,80E-03 |
| **heart development** | 31 | 6,20E-03 |
| **regulation of proteolysis** | 31 | 8,20E-03 |
| **positive regulation of cell death** | 31 | 9,10E-03 |
| **response to cytokine** | 31 | 2,50E-02 |
| **lymphocyte activation** | 31 | 3,00E-02 |
| **developmental growth** | 31 | 5,00E-02 |
| **positive regulation of cellular component movement** | 30 | 3,00E-04 |
| **skeletal system development** | 30 | 7,50E-04 |
| **single-organism carbohydrate metabolic process** | 30 | 1,50E-02 |
| **tissue morphogenesis** | 30 | 2,60E-02 |
| **regulation of growth** | 30 | 5,10E-02 |
| **positive regulation of cell motility** | 29 | 4,60E-04 |
| **positive regulation of locomotion** | 29 | 7,10E-04 |
| **positive regulation of immune response** | 29 | 7,10E-04 |
| **positive regulation of transferase activity** | 29 | 1,30E-03 |
| **lipid biosynthetic process** | 29 | 5,30E-03 |
| **negative regulation of immune system process** | 28 | 1,30E-04 |
| **regulation of protein serine/threonine kinase activity** | 28 | 1,30E-04 |
| **response to molecule of bacterial origin** | 28 | 1,90E-04 |
| **positive regulation of cell migration** | 28 | 6,00E-04 |
| **positive regulation of hydrolase activity** | 28 | 1,20E-03 |
| **gland development** | 28 | 2,30E-03 |
| **regulation of cell activation** | 28 | 4,80E-03 |
| **endocytosis** | 28 | 6,10E-03 |
| **morphogenesis of an epithelium** | 28 | 8,00E-03 |
| **cellular response to cytokine stimulus** | 28 | 8,60E-03 |
| **regulation of ion transport** | 28 | 2,10E-02 |
| **defense response to other organism** | 28 | 3,90E-02 |
| **interspecies interaction between organisms** | 28 | 5,60E-02 |
| **symbiosis, encompassing mutualism through parasitism** | 28 | 5,60E-02 |
| **carbohydrate derivative biosynthetic process** | 28 | 9,00E-02 |
| **negative regulation of cellular component organization** | 28 | 9,60E-02 |
| **positive regulation of kinase activity** | 27 | 6,10E-04 |
| **angiogenesis** | 27 | 1,20E-03 |
| **positive regulation of cell development** | 27 | 4,30E-02 |
| **response to growth factor** | 27 | 6,70E-02 |
| **positive regulation of cytokine production** | 26 | 2,00E-04 |
| **response to lipopolysaccharide** | 26 | 6,10E-04 |
| **regulation of cytoskeleton organization** | 26 | 2,40E-03 |
| **regulation of multi-organism process** | 26 | 2,40E-03 |
| **embryonic organ development** | 26 | 9,70E-03 |
| **small GTPase mediated signal transduction** | 26 | 1,10E-02 |
| **viral process** | 26 | 6,20E-02 |
| **multi-organism cellular process** | 26 | 6,70E-02 |
| **cellular response to growth factor stimulus** | 26 | 7,20E-02 |
| **positive regulation of organelle organization** | 26 | 7,50E-02 |
| **myeloid cell differentiation** | 25 | 8,40E-04 |
| **positive regulation of protein kinase activity** | 25 | 9,00E-04 |
| **regulation of immune effector process** | 25 | 2,70E-03 |
| **transmembrane receptor protein tyrosine kinase signaling pathway** | 25 | 2,10E-02 |
| **negative regulation of phosphorus metabolic process** | 25 | 5,80E-02 |
| **negative regulation of phosphate metabolic process** | 25 | 5,80E-02 |
| **regulation of cell morphogenesis** | 25 | 8,40E-02 |
| **leukocyte migration** | 24 | 6,10E-05 |
| **regulation of actin filament-based process** | 24 | 4,10E-04 |
| **ameboidal-type cell migration** | 24 | 4,10E-04 |
| **positive regulation of MAPK cascade** | 24 | 1,70E-02 |
| **leukocyte cell-cell adhesion** | 24 | 4,00E-02 |
| **regulation of anatomical structure size** | 24 | 6,40E-02 |
| **positive regulation of nervous system development** | 24 | 9,20E-02 |
| **regulation of MAP kinase activity** | 23 | 5,30E-05 |
| **activation of immune response** | 23 | 3,00E-04 |
| **actin filament organization** | 23 | 6,90E-04 |
| **negative regulation of hydrolase activity** | 23 | 1,80E-03 |
| **urogenital system development** | 23 | 1,90E-03 |
| **epithelial tube morphogenesis** | 23 | 2,40E-03 |
| **ossification** | 23 | 5,80E-03 |
| **tube morphogenesis** | 23 | 8,40E-03 |
| **regulation of leukocyte activation** | 23 | 3,70E-02 |
| **reproductive system development** | 23 | 5,10E-02 |
| **cellular response to organic cyclic compound** | 23 | 7,90E-02 |
| **adaptive immune response** | 22 | 9,20E-03 |
| **pattern specification process** | 22 | 5,70E-02 |
| **reproductive structure development** | 22 | 7,50E-02 |
| **regulation of system process** | 22 | 9,20E-02 |
| **immune response-regulating signaling pathway** | 21 | 4,80E-04 |
| **regulation of peptidase activity** | 21 | 6,40E-03 |
| **regulation of metal ion transport** | 21 | 8,10E-03 |
| **blood circulation** | 21 | 5,60E-02 |
| **circulatory system process** | 21 | 6,10E-02 |
| **T cell activation** | 21 | 7,80E-02 |
| **T cell aggregation** | 21 | 7,80E-02 |
| **lymphocyte aggregation** | 21 | 7,90E-02 |
| **leukocyte aggregation** | 21 | 8,80E-02 |
| **regulation of innate immune response** | 20 | 4,20E-05 |
| **negative regulation of cell migration** | 20 | 6,90E-05 |
| **negative regulation of cell motility** | 20 | 1,40E-04 |
| **negative regulation of cellular component movement** | 20 | 6,40E-04 |
| **negative regulation of locomotion** | 20 | 7,00E-04 |
| **kidney development** | 20 | 2,40E-03 |
| **renal system development** | 20 | 4,60E-03 |
| **leukocyte mediated immunity** | 20 | 7,20E-03 |
| **regulation of body fluid levels** | 20 | 1,10E-02 |
| **epithelial cell proliferation** | 20 | 2,30E-02 |
| **regulation of cell-cell adhesion** | 20 | 2,50E-02 |
| **regulation of transmembrane transport** | 20 | 4,20E-02 |
| **muscle cell differentiation** | 20 | 5,20E-02 |
| **positive regulation of cellular component biogenesis** | 20 | 8,70E-02 |
