# Supplementary material for: IKAROS is required for the measured response of NOTCH target genes upon external NOTCH signaling
Source: PLoS Genet. 2021 Mar 26;17(3):e1009478. doi: 10.1371/journal.pgen.1009478 (PMC8026084; doi:10.1371/journal.pgen.1009478)
Supplement: S7 Table — (DOCX) [file pgen.1009478.s007.docx]

**Table S7**. Tumor suppressors and oncogenes included in the list of the 223 genes characterized by the ‘additive effect’ (see Fig 2A).

| Protein | Details if needed | Tumor Suppressor | Oncogene | Tissus |
| --- | --- | --- | --- | --- |
| SMAD6 |  | X |  | NSCLC [1], oral squamous cell carcinoma [2] |
|  |  |  | X | Breast cancer [3], pancreactic cancer [4] |
| TGIF1 |  |  | X | Colorectal cancer [5], NSCLC [6], esophageal squamous cell carcinoma [5], triple-negative breast cancer [7] |
|  |  | X |  | MLL-rearranged AML [8] |
| CDH5 |  |  | X | Gastric cancer [9, 10] glioblastoma [11] |
| P21^WAF1/CIP1^ |  | X |  | Prostate cancer [12], endometrial cancer [13], thyroid cancer [14], esophageal cancer [15], AML [16, 17], CML [18], promyelocytic leukemia [19], osteosarcoma [20], colorectal cancer [21, 22] |
|  |  |  | X | Prostate cancer [23], hepatocarcinoma [24] |
| DLC1 |  | X |  | Gastric cancer [25], hepatocarcinoma [26, 27], lung squamous cell carcinoma (LSC), lung adenocarcinoma, breast cancer [27] |
| EIF2AK2 |  | X |  | highly metastatic breast cancer, prostate cancer and colorectal adenocarcinoma [28] |
| TP53 |  | X |  | Several cancers (reviewed in [29]) |
| ARF4 |  |  | X | Breast, lung cancer and glioma [30], epithelial ovarian cancer [31] |
| AXL |  |  | X | Ovarian [32], colorectal cancer [33], breast cancer [34], and others [35, 36], NSCLC [37], AML [38, 39], B-CLL [40], CML [41] |
| CD38 |  | X |  | CML [42], B-ALL [43], prostate cancer [44] |
|  |  |  | X | Lung [45], cervical carcinoma [46, 47], multiple myeloma [48], Immunosuppression and tumor escape [49], T-CLL [50], B-CLL [51] |
| SH3RF1 |  | X |  | Skin cancer [52], lung adenocarcinoma [53] |
| ADAR |  |  | X | NSCLC [54] |
| GAS6 (binds to TAM family receptors (example: AXL) |  |  | X | Colon , thyroid, breast, lung carcinomas, ovarian cancer and others (reviewed in [55] and AML [56, 57] |
|  |  | X |  | Colorectal cancer [58] |
| IL6 |  |  | X | Ovarian cancer [59], gastric cancer [60], hematological malignancies (reviewed in [61] |
| SGK1 |  |  | X | Colorectal cancer [62], glioblastoma multiforme [63], thyroid cancer [64], NSCLC [65], breast cancer [66, 67] |
| MDM2 |  |  | X | Several cancers (reviewed in [68, 69] |
| CSF-1 |  |  | X | Myeloproliferative neoplasms [70], tumor-associated macrophages [71], breast cancer [72], lymphoid neoplasms [73] |
| TRAF5 |  | X |  | Diffuse large B-cell lymphoma [74] |
| MAGE-D1 (or NRAGE) |  | X |  | Breast [75], pancreatic [76], colorectal cancers [77] |
| SOX12 |  |  | X | Hepatocellular carcinoma [78, 79], AML [80] |
| AHR |  |  | X | Several cancers (reviewed in [81, 82] |
| SNAI1 |  |  | X | Breast cancer [83], ovarian carcinoma [84], prostate cancer [85], AML [86] |
| MAFb |  |  | X | T-ALL [87], nasopharyngeal carcinoma [88] |
| PTGER4 |  | X |  | B-lymphoma [89] |
|  |  |  | X | Gastric cancer [90], breast cancer [91], AML [92] |
| NFKBIE |  | X |  | primary mediastinal B-cell lymphoma [93], B-CLL [94] |
| CD300a |  |  | X | AML [95], diffuse large B-cell lymphoma [96] |
|  |  | X |  | NSCLC [97] |
| B2M |  |  | X | AML [98], ALL [99] |
| C1QA |  | X |  | Ovarian [100], prostate cancer [101], mammary carcinoma [102] |
| NOD1 |  | X |  | Breast [103] and colon cancers [104] |
| CCL24 |  |  | X | Hepatocellular carcinoma [105], colorectal neoplasm [106, 107] |
| CXCL16 |  |  | X | NSCLC [108], prostate, breast, ovarian, colon, liver [109] |
| MMP14 |  |  | X | Nasopharyngeal carcinoma [110], triple-negative breast cancer [111], lymphoma [112] (via IL6), AML [113] |
| MYLK |  | X |  | NSCLC [114] |
| SEMA4C |  |  | X | Breast and ovarian cancers [115, 116] |
| S1PR1 |  |  | X | AML [117], hodgkinien lymphoma [118], mammary carcinoma [119], B-lymphoma [120] |
|  |  | X |  | CLL [121] |
| ABCC1 (MRP1) |  |  | X | AML [122], glioblastoma [123], small cell lung cancer [124], epithelial ovarian [125], and colorectal cancer [126], CML [18] |
| LOX | Mature peptide |  | X | Myeloproliferative neoplasms [127, 128], oral squamous cell carcinoma [129], breast cancer [130] |
|  | pro-peptide | X |  | Gastric [131] and prostate cancer [132], hepatocellular carcinoma [133], osteosarcoma [134] |
| OXTR (Oxytocin) |  |  | X | Prostate cancer [135] |
|  |  | X |  | Ovarian [136] and breast cancer [137-139] |
| PGF (placenta growth factor) |  |  | X | Colorectal cancer [140], Ph+ALL [141], breast cancer [142] |
| MIXL1 |  |  | X | AML [143], non-Hodgkin and Hodgkin lymphomas [144] |
| ADM (Adrenomedullin) |  |  | X | Ovarian, breast, lung, prostate cancers and glioblastoma, melanoma [145], AML [146] |
| ECE-1 (Endothelin converting enzyme 1) |  |  | X | Breast [147] and ovarian [148] |
| ZFP36L1 |  | X |  | T-ALL [149], AML [150], B-malignancies [151], bladder and breast cancer [152], colorectal cancer [153] |
| TRIM26 |  | X |  | Hepatocellular carcinoma [154], NSCLC [155] |
| COL5A1 |  |  | X | Lung adenocarcinoma [156], breast invasive ductal carcinoma [157] |
| ITGAX |  |  | X | Ovarian [158] and prostate cancers [159] |
| LAMA4 |  |  | X | Renal cell carcinoma [160], triple-negative breast cancer [161], gastric cancer [162] |
| PECAM-1 |  |  | X | Ph+ leukemias [163], AML [95] |
| SORBS3 |  | X |  | Hepatocellular carcinoma [164] |
| ALDOC |  |  | X | Colorectal cancer [165] |
| NR4A1 |  | X |  | AML [166], myeloproliferative neoplasms [167, 168], aggressive lymphoma [169], triple-negative breast cancer [170], gastric cancer [171] |
|  |  |  | X | NSCLC [172] |
| APELIN |  |  | X | Gastric cancer [173], lymphoma [174] |
| CRLF1 |  |  | X | Papillary thyroid carcinoma [175] |
| EPHB2 |  | X |  | Prostate [176] and colorectal cancers [177, 178] |
|  |  |  | X | Cervical cancer [179] |
| LIMK1 |  |  | X | NSCLC [180], pancreatic [181], gastric [182], colorectal [183] and colon cancers [184], AML [185] and highly invasive breast and prostate cancers [186] |
| MINK1 |  |  | X | Breast cancer [187] |
|  |  | X |  | In Ovarian Surface Epithelial Cells transformation [188] |
| PIP5K1A |  |  | X | Prostate [189] and breast cancers [190] |

**REFERENCES:**

1. Jeon HS, Dracheva T, Yang SH, Meerzaman D, Fukuoka J, Shakoori A, et al. SMAD6 contributes to patient survival in non-small cell lung cancer and its knockdown reestablishes TGF-beta homeostasis in lung cancer cells. Cancer Res. 2008;68(23):9686-92. Epub 2008/12/03. doi: 10.1158/0008-5472.CAN-08-1083. PubMed PMID: 19047146; PubMed Central PMCID: PMCPMC3617041.

2. Mangone FRR, Walder F, Maistro S, Pasini FS, Lehn CN, Carvalho MB, et al. Smad2 and Smad6 as predictors of overall survival in oral squamous cell carcinoma patients. Molecular Cancer. 2010;9(1):106. doi: 10.1186/1476-4598-9-106.

3. de Boeck M, Cui C, Mulder AA, Jost CR, Ikeno S, ten Dijke P. Smad6 determines BMP-regulated invasive behaviour of breast cancer cells in a zebrafish xenograft model. Scientific Reports. 2016;6:24968. doi: 10.1038/srep24968.

4. Kleeff J, Maruyama H, Friess H, Buchler MW, Falb D, Korc M. Smad6 suppresses TGF-beta-induced growth inhibition in COLO-357 pancreatic cancer cells and is overexpressed in pancreatic cancer. Biochem Biophys Res Commun. 1999;255(2):268-73. Epub 1999/03/02. doi: 10.1006/bbrc.1999.0171. PubMed PMID: 10049697.

5. Wang Y, Pan T, Li L, Wang H, Li J, Zhang D, et al. Knockdown of TGIF attenuates the proliferation and tumorigenicity of EC109 cells and promotes cisplatin-induced apoptosis. Oncology letters. 2017;14(6):6519-24. Epub 2017/09/21. doi: 10.3892/ol.2017.7009. PubMed PMID: 29344116.

6. Xiang G, Yi Y, Weiwei H, Weiming W. TGIF1 promoted the growth and migration of cancer cells in nonsmall cell lung cancer. Tumour biology : the journal of the International Society for Oncodevelopmental Biology and Medicine. 2015;36(12):9303-10. Epub 2015/06/25. doi: 10.1007/s13277-015-3676-8. PubMed PMID: 26104768.

7. Zhang MZ, Ferrigno O, Wang Z, Ohnishi M, Prunier C, Levy L, et al. TGIF governs a feed-forward network that empowers Wnt signaling to drive mammary tumorigenesis. Cancer Cell. 2015;27(4):547-60. Epub 2015/04/16. doi: 10.1016/j.ccell.2015.03.002. PubMed PMID: 25873176; PubMed Central PMCID: PMCPMC4398914.

8. Willer A, Jakobsen JS, Ohlsson E, Rapin N, Waage J, Billing M, et al. TGIF1 is a negative regulator of MLL-rearranged acute myeloid leukemia. Leukemia. 2015;29(5):1018-31. Epub 2014/10/29. doi: 10.1038/leu.2014.307. PubMed PMID: 25349154.

9. Inokuchi M, Higuchi K, Takagi Y, Tanioka T, Nakagawa M, Gokita K, et al. Cadherin 5 Is a Significant Risk Factor for Hematogenous Recurrence and a Prognostic Factor in Locally Advanced Gastric Cancer. Anticancer research. 2017;37(12):6807-13. Epub 2017/12/01. doi: 10.21873/anticanres.12141. PubMed PMID: 29187459.

10. Higuchi K, Inokuchi M, Takagi Y, Ishikawa T, Otsuki S, Uetake H, et al. Cadherin 5 expression correlates with poor survival in human gastric cancer. Journal of clinical pathology. 2017;70(3):217-21. Epub 2016/07/29. doi: 10.1136/jclinpath-2016-203640. PubMed PMID: 27466381.

11. Mao XG, Xue XY, Wang L, Zhang X, Yan M, Tu YY, et al. CDH5 is specifically activated in glioblastoma stemlike cells and contributes to vasculogenic mimicry induced by hypoxia. Neuro-oncology. 2013;15(7):865-79. Epub 2013/05/07. doi: 10.1093/neuonc/not029. PubMed PMID: 23645533; PubMed Central PMCID: PMCPMC3688011.

12. Li S, Wang C, Yu X, Wu H, Hu J, Wang S, et al. miR-3619-5p inhibits prostate cancer cell growth by activating CDKN1A expression. Oncology reports. 2017;37(1):241-8. Epub 2016/11/24. doi: 10.3892/or.2016.5250. PubMed PMID: 27878260.

13. Yamawaki K, Ishiguro T, Mori Y, Yoshihara K, Suda K, Tamura R, et al. Sox2-dependent inhibition of p21 is associated with poor prognosis of endometrial cancer. Cancer Sci. 2017;108(4):632-40. Epub 2017/02/12. doi: 10.1111/cas.13196. PubMed PMID: 28188685; PubMed Central PMCID: PMCPMC5406528.

14. Lei S-t, Shen F, Chen J-w, Feng J-h, Cai W-s, Shen L, et al. MiR-639 promoted cell proliferation and cell cycle in human thyroid cancer by suppressing CDKN1A expression. Biomedicine & Pharmacotherapy. 2016;84:1834-40. doi: <https://doi.org/10.1016/j.biopha.2016.10.087>.

15. Luo J, Zhang C, Wang C, Li L, Li C, Li Q, et al. Miz-1 promotes the proliferation of esophageal cancer cells via suppression of p21 and release of p21-arrested cyclin D1. Oncology reports. 2016;35(6):3532-40. Epub 2016/04/26. doi: 10.3892/or.2016.4731. PubMed PMID: 27109891.

16. Zhang X, Ma W, Cui J, Yao H, Zhou H, Ge Y, et al. Regulation of p21 by TWIST2 contributes to its tumor-suppressor function in human acute myeloid leukemia. Oncogene. 2015;34(23):3000-10. Epub 2014/08/05. doi: 10.1038/onc.2014.241. PubMed PMID: 25088197.

17. Geyer CR. Strategies to re-express epigenetically silenced p15(INK4b) and p21(WAF1) genes in acute myeloid leukemia. Epigenetics. 2010;5(8):696-703. Epub 2010/12/03. doi: 10.4161/epi.5.8.13276. PubMed PMID: 21124069.

18. Chen JR, Jia XH, Wang H, Yi YJ, Wang JY, Li YJ. Timosaponin A-III reverses multi-drug resistance in human chronic myelogenous leukemia K562/ADM cells via downregulation of MDR1 and MRP1 expression by inhibiting PI3K/Akt signaling pathway. International journal of oncology. 2016;48(5):2063-70. Epub 2016/03/18. doi: 10.3892/ijo.2016.3423. PubMed PMID: 26984633.

19. Atashrazm F, Lowenthal RM, Woods GM, Holloway AF, Karpiniec SS, Dickinson JL. Fucoidan Suppresses the Growth of Human Acute Promyelocytic Leukemia Cells In Vitro and In Vivo. J Cell Physiol. 2016;231(3):688-97. Epub 2015/08/05. doi: 10.1002/jcp.25119. PubMed PMID: 26241708.

20. Xu T, Jiang W, Fan L, Gao Q, Li G. Upregulation of long noncoding RNA Xist promotes proliferation of osteosarcoma by epigenetic silencing of P21. Oncotarget. 2017;8(60):101406-17. Epub 2017/12/20. doi: 10.18632/oncotarget.20738. PubMed PMID: 29254174; PubMed Central PMCID: PMCPMC5731884.

21. Zhang Q, Shim K, Wright K, Jurkevich A, Khare S. Atypical role of sprouty in p21 dependent inhibition of cell proliferation in colorectal cancer. Molecular carcinogenesis. 2016;55(9):1355-68. Epub 2015/08/22. doi: 10.1002/mc.22379. PubMed PMID: 26293890; PubMed Central PMCID: PMCPMC4873464.

22. Wang JL, Qi Z, Li YH, Zhao HM, Chen YG, Fu W. TGFbeta induced factor homeobox 1 promotes colorectal cancer development through activating Wnt/beta-catenin signaling. Oncotarget. 2017;8(41):70214-25. Epub 2017/10/21. doi: 10.18632/oncotarget.19603. PubMed PMID: 29050273; PubMed Central PMCID: PMCPMC5642548.

23. Jain AK, Raina K, Agarwal R. Deletion of p21/Cdkn1a confers protective effect against prostate tumorigenesis in transgenic adenocarcinoma of the mouse prostate model. Cell Cycle. 2013;12(10):1598-604. Epub 2013/04/30. doi: 10.4161/cc.24741. PubMed PMID: 23624841; PubMed Central PMCID: PMCPMC3680539.

24. Ohkoshi S, Yano M, Matsuda Y. Oncogenic role of p21 in hepatocarcinogenesis suggests a new treatment strategy. World journal of gastroenterology. 2015;21(42):12150-6. Epub 2015/11/14. doi: 10.3748/wjg.v21.i42.12150. PubMed PMID: 26576099.

25. Su Y, Lin L, Zhang J, Jiang Y, Pan C, Sun L, et al. Low expression of DLC1 is predictive of poor therapeutic efficiency of fluoropyrimidine and oxaliplatin as adjuvant chemotherapy in gastric cancer. Molecular medicine reports. 2015;12(4):5771-9. Epub 2015/08/05. doi: 10.3892/mmr.2015.4173. PubMed PMID: 26239822; PubMed Central PMCID: PMCPMC4581752.

26. Wu H-T, Xie C-R, Lv J, Qi H-Q, Wang F, Zhang S, et al. The tumor suppressor DLC1 inhibits cancer progression and oncogenic autophagy in hepatocellular carcinoma. Laboratory Investigation. 2018;98(8):1014-24. doi: 10.1038/s41374-018-0062-3.

27. Wang D, Qian X, Rajaram M, Durkin ME, Lowy DR. DLC1 is the principal biologically-relevant down-regulated DLC family member in several cancers. Oncotarget. 2016;7(29):45144-57. Epub 2016/05/14. doi: 10.18632/oncotarget.9266. PubMed PMID: 27174913; PubMed Central PMCID: PMCPMC5216712.

28. Kim TH, Cho SG. Kisspeptin inhibits cancer growth and metastasis via activation of EIF2AK2. Molecular medicine reports. 2017;16(5):7585-90. Epub 2017/09/26. doi: 10.3892/mmr.2017.7578. PubMed PMID: 28944853.

29. Aubrey BJ, Strasser A, Kelly GL. Tumor-Suppressor Functions of the TP53 Pathway. Cold Spring Harbor perspectives in medicine. 2016;6(5). Epub 2016/05/04. doi: 10.1101/cshperspect.a026062. PubMed PMID: 27141080; PubMed Central PMCID: PMCPMC4852799.

30. Casalou C, Faustino A, Barral DC. Arf proteins in cancer cell migration. Small GTPases. 2016;7(4):270-82. doi: 10.1080/21541248.2016.1228792. PubMed PMID: 27589148.

31. Wu Q, Ren X, Zhang Y, Fu X, Li Y, Peng Y, et al. MiR-221-3p targets ARF4 and inhibits the proliferation and migration of epithelial ovarian cancer cells. Biochem Biophys Res Commun. 2018;497(4):1162-70. Epub 2017/01/07. doi: 10.1016/j.bbrc.2017.01.002. PubMed PMID: 28057486.

32. Kanlikilicer P, Ozpolat B, Aslan B, Bayraktar R, Gurbuz N, Rodriguez-Aguayo C, et al. Therapeutic Targeting of AXL Receptor Tyrosine Kinase Inhibits Tumor Growth and Intraperitoneal Metastasis in Ovarian Cancer Models. Molecular therapy Nucleic acids. 2017;9:251-62. Epub 2017/12/17. doi: 10.1016/j.omtn.2017.06.023. PubMed PMID: 29246304; PubMed Central PMCID: PMCPMC5675720.

33. Uribe DJ, Mandell EK, Watson A, Martinez JD, Leighton JA, Ghosh S, et al. The receptor tyrosine kinase AXL promotes migration and invasion in colorectal cancer. PLoS One. 2017;12(7):e0179979. Epub 2017/07/21. doi: 10.1371/journal.pone.0179979. PubMed PMID: 28727830; PubMed Central PMCID: PMCPMC5519024.

34. Goyette MA, Duhamel S, Aubert L, Pelletier A, Savage P, Thibault MP, et al. The Receptor Tyrosine Kinase AXL Is Required at Multiple Steps of the Metastatic Cascade during HER2-Positive Breast Cancer Progression. Cell Rep. 2018;23(5):1476-90. Epub 2018/05/03. doi: 10.1016/j.celrep.2018.04.019. PubMed PMID: 29719259.

35. Corno C, Gatti L, Lanzi C, Zaffaroni N, Colombo D, Perego P. Role of the Receptor Tyrosine Kinase Axl and its Targeting in Cancer Cells. Current medicinal chemistry. 2016;23(15):1496-512. Epub 2016/04/07. PubMed PMID: 27048336.

36. Rankin EB, Giaccia AJ. The Receptor Tyrosine Kinase AXL in Cancer Progression. Cancers. 2016;8(11). Epub 2016/11/12. doi: 10.3390/cancers8110103. PubMed PMID: 27834845; PubMed Central PMCID: PMCPMC5126763.

37. Zhang G, Wang M, Zhao H, Cui W. Function of Axl receptor tyrosine kinase in non-small cell lung cancer. Oncol Lett. 2018;15(3):2726-34. Epub 2018/02/13. doi: 10.3892/ol.2017.7694. PubMed PMID: 29434997; PubMed Central PMCID: PMCPMC5778882.

38. Hong CC, Lay JD, Huang JS, Cheng AL, Tang JL, Lin MT, et al. Receptor tyrosine kinase AXL is induced by chemotherapy drugs and overexpression of AXL confers drug resistance in acute myeloid leukemia. Cancer letters. 2008;268(2):314-24. Epub 2008/05/27. doi: 10.1016/j.canlet.2008.04.017. PubMed PMID: 18502572.

39. Janning M, Ben-Batalla I, Loges S. Axl inhibition: a potential road to a novel acute myeloid leukemia therapy? Expert review of hematology. 2015;8(2):135-8. Epub 2015/01/13. doi: 10.1586/17474086.2015.997704. PubMed PMID: 25578023.

40. Sinha S, Boysen J, Nelson M, Warner SL, Bearss D, Kay NE, et al. Axl activates fibroblast growth factor receptor pathway to potentiate survival signals in B-cell chronic lymphocytic leukemia cells. Leukemia. 2016;30(6):1431-6. Epub 2015/11/26. doi: 10.1038/leu.2015.323. PubMed PMID: 26598018; PubMed Central PMCID: PMCPMC4879100.

41. Dufies M, Jacquel A, Belhacene N, Robert G, Cluzeau T, Luciano F, et al. Mechanisms of AXL overexpression and function in Imatinib-resistant chronic myeloid leukemia cells. Oncotarget. 2011;2(11):874-85. Epub 2011/12/06. doi: 10.18632/oncotarget.360. PubMed PMID: 22141136; PubMed Central PMCID: PMCPMC3259992.

42. Wang Z, Liu Z, Wu X, Chu S, Wang J, Yuan H, et al. ATRA-induced cellular differentiation and CD38 expression inhibits acquisition of BCR-ABL mutations for CML acquired resistance. PLoS Genet. 2014;10(6):e1004414. Epub 2014/06/27. doi: 10.1371/journal.pgen.1004414. PubMed PMID: 24967705; PubMed Central PMCID: PMCPMC4072521.

43. Jiang Z, Wu D, Lin S, Li P. CD34 and CD38 are prognostic biomarkers for acute B lymphoblastic leukemia. Biomarker research. 2016;4:23. Epub 2016/12/27. doi: 10.1186/s40364-016-0080-5. PubMed PMID: 28018598; PubMed Central PMCID: PMCPMC5159997.

44. Liu X, Grogan TR, Hieronymus H, Hashimoto T, Mottahedeh J, Cheng D, et al. Low CD38 Identifies Progenitor-like Inflammation-Associated Luminal Cells that Can Initiate Human Prostate Cancer and Predict Poor Outcome. Cell Rep. 2016;17(10):2596-606. Epub 2016/12/08. doi: 10.1016/j.celrep.2016.11.010. PubMed PMID: 27926864; PubMed Central PMCID: PMCPMC5367888.

45. Bu X, Kato J, Hong JA, Merino MJ, Schrump DS, Lund FE, et al. CD38 knockout suppresses tumorigenesis in mice and clonogenic growth of human lung cancer cells. Carcinogenesis. 2018;39(2):242-51. Epub 2017/12/12. doi: 10.1093/carcin/bgx137. PubMed PMID: 29228209; PubMed Central PMCID: PMCPMC5862338.

46. Liao S, Xiao S, Zhu G, Zheng D, He J, Pei Z, et al. CD38 is highly expressed and affects the PI3K/Akt signaling pathway in cervical cancer. Oncology reports. 2014;32(6):2703-9. Epub 2014/10/14. doi: 10.3892/or.2014.3537. PubMed PMID: 25310288.

47. Liao S, Xiao S, Chen H, Zhang M, Chen Z, Long Y, et al. CD38 enhances the proliferation and inhibits the apoptosis of cervical cancer cells by affecting the mitochondria functions. Molecular carcinogenesis. 2017;56(10):2245-57. Epub 2017/05/26. doi: 10.1002/mc.22677. PubMed PMID: 28544069.

48. van de Donk NW, Janmaat ML, Mutis T, Lammerts van Bueren JJ, Ahmadi T, Sasser AK, et al. Monoclonal antibodies targeting CD38 in hematological malignancies and beyond. Immunological reviews. 2016;270(1):95-112. Epub 2016/02/13. doi: 10.1111/imr.12389. PubMed PMID: 26864107; PubMed Central PMCID: PMCPMC4755228.

49. Chen L, Diao L, Yang Y, Yi X, Rodriguez BL, Li Y, et al. CD38-mediated immunosuppression as a mechanism of tumor cell escape from PD-1/PD-L1 blockade. Cancer Discovery. 2018:CD-17-1033. doi: 10.1158/2159-8290.CD-17-1033.

50. Manna A, Lewis-Tuffin LJ, Ailawadhi S, Chanan-Khan AA, Paulus A. Using anti-CD38 immunotherapy to enhance anti-tumor T-cell immunity in chronic lymphocytic leukemia (CLL). The Journal of Immunology. 2018;200(1 Supplement):58.17.

51. Xu W, Li JY, Wu YJ, Yu H, Shen QD, Tian T, et al. CD38 as a prognostic factor in Chinese patients with chronic lymphocytic leukaemia. Leukemia research. 2009;33(2):237-43. Epub 2008/08/05. doi: 10.1016/j.leukres.2008.06.026. PubMed PMID: 18674817.

52. Ying Z, Sandoval M, Beronja S. Oncogenic activation of PI3K induces progenitor cell differentiation to suppress epidermal growth. Nature cell biology. 2018;20(11):1256-66. Epub 2018/10/27. doi: 10.1038/s41556-018-0218-9. PubMed PMID: 30361695; PubMed Central PMCID: PMCPMC6291208.

53. Wang G, Bie F, Qu X, Yang X, Liu S, Wang Y, et al. Expression profiling of ubiquitin-related genes in LKB1 mutant lung adenocarcinoma. Sci Rep. 2018;8(1):13221. Epub 2018/09/07. doi: 10.1038/s41598-018-31592-2. PubMed PMID: 30185829; PubMed Central PMCID: PMCPMC6125361.

54. Amin EM, Liu Y, Deng S, Tan KS, Chudgar N, Mayo MW, et al. The RNA-editing enzyme ADAR promotes lung adenocarcinoma migration and invasion by stabilizing &lt;em&gt;FAK&lt;/em&gt. Science Signaling. 2017;10(497):eaah3941. doi: 10.1126/scisignal.aah3941.

55. Wu G, Ma Z, Hu W, Wang D, Gong B, Fan C, et al. Molecular insights of Gas6/TAM in cancer development and therapy. Cell Death &Amp; Disease. 2017;8:e2700. doi: 10.1038/cddis.2017.113.

56. Lee-Sherick AB, Eisenman KM, Sather S, McGranahan A, Armistead PM, McGary CS, et al. Aberrant Mer receptor tyrosine kinase expression contributes to leukemogenesis in acute myeloid leukemia. Oncogene. 2013;32(46):5359-68. Epub 2013/03/12. doi: 10.1038/onc.2013.40. PubMed PMID: 23474756; PubMed Central PMCID: PMCPMC3898106.

57. Whitman SP, Kohlschmidt J, Maharry K, Volinia S, Mrozek K, Nicolet D, et al. GAS6 expression identifies high-risk adult AML patients: potential implications for therapy. Leukemia. 2014;28(6):1252-8. Epub 2013/12/12. doi: 10.1038/leu.2013.371. PubMed PMID: 24326683; PubMed Central PMCID: PMCPMC4047202.

58. Akitake-Kawano R, Seno H, Nakatsuji M, Kimura Y, Nakanishi Y, Yoshioka T, et al. Inhibitory role of Gas6 in intestinal tumorigenesis. Carcinogenesis. 2013;34(7):1567-74. Epub 2013/02/23. doi: 10.1093/carcin/bgt069. PubMed PMID: 23430954.

59. Zou M, Zhang X, Xu C. IL6-induced metastasis modulators p-STAT3, MMP-2 and MMP-9 are targets of 3,3'-diindolylmethane in ovarian cancer cells. Cellular oncology (Dordrecht). 2016;39(1):47-57. Epub 2015/10/30. doi: 10.1007/s13402-015-0251-7. PubMed PMID: 26510945.

60. Wu X, Tao P, Zhou Q, Li J, Yu Z, Wang X, et al. IL-6 secreted by cancer-associated fibroblasts promotes epithelial-mesenchymal transition and metastasis of gastric cancer via JAK2/STAT3 signaling pathway. Oncotarget. 2017;8(13):20741-50. Epub 2017/02/12. doi: 10.18632/oncotarget.15119. PubMed PMID: 28186964; PubMed Central PMCID: PMCPMC5400541.

61. Burger R. Impact of interleukin-6 in hematological malignancies. Transfusion medicine and hemotherapy : offizielles Organ der Deutschen Gesellschaft fur Transfusionsmedizin und Immunhamatologie. 2013;40(5):336-43. Epub 2013/07/19. doi: 10.1159/000354194. PubMed PMID: 24273487.

62. Liang X, Lan C, Jiao G, Fu W, Long X, An Y, et al. Therapeutic inhibition of SGK1 suppresses colorectal cancer. Experimental &Amp; Molecular Medicine. 2017;49:e399. doi: 10.1038/emm.2017.184 <https://www.nature.com/articles/emm2017184#supplementary-information>.

63. Lehrer S, Rheinstein PH, Rosenzweig KE. Glioblastoma Multiforme: Fewer Tumor Copy Number Segments of the SGK1 Gene Are Associated with Poorer Survival. Cancer genomics & proteomics. 2018;15(4):273-8. Epub 2018/07/07. doi: 10.21873/cgp.20085. PubMed PMID: 29976632; PubMed Central PMCID: PMCPMC6070715.

64. Orlacchio A, Ranieri M, Brave M, Arciuch VA, Forde T, De Martino D, et al. SGK1 Is a Critical Component of an AKT-Independent Pathway Essential for PI3K-Mediated Tumor Development and Maintenance. Cancer Res. 2017;77(24):6914-26. Epub 2017/10/22. doi: 10.1158/0008-5472.Can-17-2105. PubMed PMID: 29055016; PubMed Central PMCID: PMCPMC5732884.

65. Tang Z, Shen Q, Xie H, Zhou Z, Shi G, Zhang C, et al. Serum and glucocorticoid-regulated kinase 1 (SGK1) is a predictor of poor prognosis in non-small cell lung cancer, and its dynamic pattern following treatment with SGK1 inhibitor and gamma-ray irradiation was elucidated. Oncology reports. 2018;39(3):1505-15. Epub 2018/01/13. doi: 10.3892/or.2018.6181. PubMed PMID: 29328462.

66. Eirew P, Steif A, Khattra J, Ha G, Yap D, Farahani H, et al. Dynamics of genomic clones in breast cancer patient xenografts at single-cell resolution. Nature. 2015;518(7539):422-6. Epub 2014/12/04. doi: 10.1038/nature13952. PubMed PMID: 25470049; PubMed Central PMCID: PMCPMC4864027.

67. Sahoo S, Brickley DR, Kocherginsky M, Conzen SD. Coordinate expression of the PI3-kinase downstream effectors serum and glucocorticoid-induced kinase (SGK-1) and Akt-1 in human breast cancer. European journal of cancer (Oxford, England : 1990). 2005;41(17):2754-9. Epub 2005/10/26. doi: 10.1016/j.ejca.2005.07.018. PubMed PMID: 16246546.

68. Zhao Y, Yu H, Hu W. The regulation of MDM2 oncogene and its impact on human cancers. Acta biochimica et biophysica Sinica. 2014;46(3):180-9. Epub 2014/01/03. doi: 10.1093/abbs/gmt147. PubMed PMID: 24389645.

69. Karni-Schmidt O, Lokshin M, Prives C. The Roles of MDM2 and MDMX in Cancer. Annual review of pathology. 2016;11:617-44. Epub 2016/03/30. doi: 10.1146/annurev-pathol-012414-040349. PubMed PMID: 27022975; PubMed Central PMCID: PMCPMC6028239.

70. Gilbert HS, Praloran V, Stanley ER. Increased circulating CSF-1 (M-CSF) in myeloproliferative disease: association with myeloid metaplasia and peripheral bone marrow extension. Blood. 1989;74(4):1231-4. Epub 1989/09/01. PubMed PMID: 2669997.

71. Thompson TW, Jackson BT, Li PJ, Wang J, Kim AB, Huang KTH, et al. Tumor-derived CSF-1 induces the NKG2D ligand RAE-1delta on tumor-infiltrating macrophages. eLife. 2018;7. Epub 2018/05/15. doi: 10.7554/eLife.32919. PubMed PMID: 29757143; PubMed Central PMCID: PMCPMC5991831.

72. Richardsen E, Uglehus RD, Johnsen SH, Busund LT. Macrophage-colony stimulating factor (CSF1) predicts breast cancer progression and mortality. Anticancer research. 2015;35(2):865-74. Epub 2015/02/11. PubMed PMID: 25667468.

73. Janowska-Wieczorek A, Belch AR, Jacobs A, Bowen D, Padua RA, Paietta E, et al. Increased circulating colony-stimulating factor-1 in patients with preleukemia, leukemia, and lymphoid malignancies. Blood. 1991;77(8):1796-803. Epub 1991/04/15. PubMed PMID: 2015402.

74. Compagno M, Lim WK, Grunn A, Nandula SV, Brahmachary M, Shen Q, et al. Mutations of multiple genes cause deregulation of NF-kappaB in diffuse large B-cell lymphoma. Nature. 2009;459(7247):717-21. Epub 2009/05/05. doi: 10.1038/nature07968. PubMed PMID: 19412164; PubMed Central PMCID: PMCPMC2973325.

75. Du Q, Zhang Y, Tian XX, Li Y, Fang WG. MAGE-D1 inhibits proliferation, migration and invasion of human breast cancer cells. Oncology reports. 2009;22(3):659-65. Epub 2009/07/30. PubMed PMID: 19639218.

76. Chu CS, Xue B, Tu C, Feng ZH, Shi YH, Miao Y, et al. NRAGE suppresses metastasis of melanoma and pancreatic cancer in vitro and in vivo. Cancer letters. 2007;250(2):268-75. Epub 2006/12/05. doi: 10.1016/j.canlet.2006.10.020. PubMed PMID: 17140727.

77. Zeng Z-l, Wu W-j, Yang J, Tang Z-j, Chen D-l, Qiu M-z, et al. Prognostic relevance of melanoma antigen D1 expression in colorectal carcinoma. Journal of translational medicine. 2012;10:181-. doi: 10.1186/1479-5876-10-181. PubMed PMID: 22935435.

78. Yuan P, Meng L, Wang N. SOX12 upregulation is associated with metastasis of hepatocellular carcinoma and increases CDK4 and IGF2BP1 expression. European review for medical and pharmacological sciences. 2017;21(17):3821-6. Epub 2017/10/05. PubMed PMID: 28975985.

79. Huang W, Chen Z, Shang X, Tian D, Wang D, Wu K, et al. Sox12, a direct target of FoxQ1, promotes hepatocellular carcinoma metastasis through up-regulating Twist1 and FGFBP1. Hepatology. 2015;61(6):1920-33. Epub 2015/02/24. doi: 10.1002/hep.27756. PubMed PMID: 25704764.

80. Wan H, Cai J, Chen F, Zhu J, Zhong J, Zhong H. SOX12: a novel potential target for acute myeloid leukaemia. Br J Haematol. 2017;176(3):421-30. Epub 2016/11/20. doi: 10.1111/bjh.14425. PubMed PMID: 27858992.

81. Feng S, Cao Z, Wang X. Role of aryl hydrocarbon receptor in cancer. Biochimica et biophysica acta. 2013;1836(2):197-210. Epub 2013/05/29. doi: 10.1016/j.bbcan.2013.05.001. PubMed PMID: 23711559.

82. Dietrich C, Kaina B. The aryl hydrocarbon receptor (AhR) in the regulation of cell-cell contact and tumor growth. Carcinogenesis. 2010;31(8):1319-28. Epub 2010/01/29. doi: 10.1093/carcin/bgq028. PubMed PMID: 20106901; PubMed Central PMCID: PMCPMC6276890.

83. Dhasarathy A, Phadke D, Mav D, Shah RR, Wade PA. The transcription factors Snail and Slug activate the transforming growth factor-beta signaling pathway in breast cancer. PLoS One. 2011;6(10):e26514. Epub 2011/10/27. doi: 10.1371/journal.pone.0026514. PubMed PMID: 22028892; PubMed Central PMCID: PMCPMC3197668.

84. Jin H, Yu Y, Zhang T, Zhou X, Zhou J, Jia L, et al. Snail is critical for tumor growth and metastasis of ovarian carcinoma. International journal of cancer. 2010;126(9):2102-11. Epub 2009/10/02. doi: 10.1002/ijc.24901. PubMed PMID: 19795442.

85. Deep G, Jain AK, Ramteke A, Ting H, Vijendra KC, Gangar SC, et al. SNAI1 is critical for the aggressiveness of prostate cancer cells with low E-cadherin. Mol Cancer. 2014;13:37. Epub 2014/02/26. doi: 10.1186/1476-4598-13-37. PubMed PMID: 24565133; PubMed Central PMCID: PMCPMC3937432.

86. Carmichael CL, Goossens S, Wang J, Nguyen T, Haigh K, Berx G, et al. The EMT Modulator SNAI1 Drives AML Development Via Its Interaction with the Chromatin Modulator LSD1. Blood. 2016;128(22):2688.

87. Pajcini KV, Xu L, Shao L, Petrovic J, Palasiewicz K, Ohtani Y, et al. MAFB enhances oncogenic Notch signaling in T cell acute lymphoblastic leukemia. Science Signaling. 2017;10(505):eaam6846. doi: 10.1126/scisignal.aam6846.

88. Yang W, Lan X, Li D, Li T, Lu S. MiR-223 targeting MAFB suppresses proliferation and migration of nasopharyngeal carcinoma cells. BMC cancer. 2015;15:461-. doi: 10.1186/s12885-015-1464-x. PubMed PMID: 26055874.

89. Murn J, Alibert O, Wu N, Tendil S, Gidrol X. Prostaglandin E2 regulates B cell proliferation through a candidate tumor suppressor, Ptger4. J Exp Med. 2008;205(13):3091-103. Epub 2008/12/17. doi: 10.1084/jem.20081163. PubMed PMID: 19075289; PubMed Central PMCID: PMCPMC2605229.

90. Heinrichs SKM, Hess T, Becker J, Hamann L, Vashist YK, Butterbach K, et al. Evidence for PTGER4, PSCA, and MBOAT7 as risk genes for gastric cancer on the genome and transcriptome level. Cancer medicine. 2018;7(10):5057-65. doi: 10.1002/cam4.1719. PubMed PMID: 30191681.

91. Hiken JF, McDonald JI, Decker KF, Sanchez C, Hoog J, VanderKraats ND, et al. Epigenetic activation of the prostaglandin receptor EP4 promotes resistance to endocrine therapy for breast cancer. Oncogene. 2017;36(16):2319-27. Epub 2016/11/22. doi: 10.1038/onc.2016.397. PubMed PMID: 27869171; PubMed Central PMCID: PMCPMC5398938.

92. Shin TH, Brynczka C, Dayyani F, Rivera MN, Sweetser DA. TLE4 regulation of wnt-mediated inflammation underlies its role as a tumor suppressor in myeloid leukemia. Leukemia research. 2016;48:46-56. Epub 2016/07/21. doi: 10.1016/j.leukres.2016.07.002. PubMed PMID: 27486062.

93. Mansouri L, Noerenberg D, Young E, Mylonas E, Abdulla M, Frick M, et al. Frequent NFKBIE deletions are associated with poor outcome in primary mediastinal B-cell lymphoma. Blood. 2016;128(23):2666-70. Epub 2016/09/28. doi: 10.1182/blood-2016-03-704528. PubMed PMID: 27670424.

94. Mansouri L, Sutton L-A, Ljungström V, Bondza S, Arngården L, Bhoi S, et al. Functional loss of IκBε leads to NF-κB deregulation in aggressive chronic lymphocytic leukemia. The Journal of Experimental Medicine. 2015;212(6):833. doi: 10.1084/jem.20142009.

95. Sun X, Huang S, Wang X, Zhang X, Wang X. CD300A promotes tumor progression by PECAM1, ADCY7 and AKT pathway in acute myeloid leukemia. Oncotarget. 2018;9(44):27574-84. doi: 10.18632/oncotarget.24164. PubMed PMID: 29938007.

96. Jiang L, Xu Y, Zeng X, Fang J, Morse HC, 3rd, Zhou JX. Suppression of CD300A inhibits the growth of diffuse large B-cell lymphoma. Oncotarget. 2015;6(31):31191-202. doi: 10.18632/oncotarget.5152. PubMed PMID: 26435477.

97. Tang Z, Cai H, Wang R, Cui Y. Overexpression of CD300A inhibits progression of NSCLC through downregulating Wnt/β-catenin pathway. OncoTargets and therapy. 2018;11:8875-83. doi: 10.2147/OTT.S185521. PubMed PMID: 30573974.

98. Melillo L, Cascavilla N, Lombardi G, Carotenuto M, Musto P. Prognostic relevance of serum beta 2-microglobulin in acute myeloid leukemia. Leukemia. 1992;6(10):1076-8. Epub 1992/10/01. PubMed PMID: 1405762.

99. Thomas DA, Brien S, Faderl S, Cortes J, Borthakur G, Burger JA, et al. Prognostic Significance of Beta-2 Microglobulin (B2M) in Adult Acute Lymphoblastic Leukemia (ALL). Blood. 2009;114(22):4679.

100. Kaur A, Sultan SHA, Murugaiah V, Pathan AA, Alhamlan FS, Karteris E, et al. Human C1q Induces Apoptosis in an Ovarian Cancer Cell Line via Tumor Necrosis Factor Pathway. Frontiers in immunology. 2016;7:599-. doi: 10.3389/fimmu.2016.00599. PubMed PMID: 28066412.

101. Hong Q, Sze CI, Lin SR, Lee MH, He RY, Schultz L, et al. Complement C1q activates tumor suppressor WWOX to induce apoptosis in prostate cancer cells. PLoS One. 2009;4(6):e5755. Epub 2009/06/02. doi: 10.1371/journal.pone.0005755. PubMed PMID: 19484134; PubMed Central PMCID: PMCPMC2685983.

102. Bandini S, Macagno M, Hysi A, Lanzardo S, Conti L, Bello A, et al. The non-inflammatory role of C1q during Her2/neu-driven mammary carcinogenesis. Oncoimmunology. 2016;5(12):e1253653-e. doi: 10.1080/2162402X.2016.1253653. PubMed PMID: 28123895.

103. Velloso FJ, Sogayar MC, Correa RG. Expression and in vitro assessment of tumorigenicity for NOD1 and NOD2 receptors in breast cancer cell lines. BMC research notes. 2018;11(1):222-. doi: 10.1186/s13104-018-3335-4. PubMed PMID: 29615116.

104. Chen GY, Shaw MH, Redondo G, Núñez G. The Innate Immune Receptor Nod1 Protects the Intestine from Inflammation-Induced Tumorigenesis. Cancer Research. 2008;68(24):10060. doi: 10.1158/0008-5472.CAN-08-2061.

105. Jin L, Liu W-R, Tian M-X, Jiang X-F, Wang H, Zhou P-Y, et al. CCL24 contributes to HCC malignancy via RhoB- VEGFA-VEGFR2 angiogenesis pathway and indicates poor prognosis. Oncotarget. 2016;8(3):5135-48. doi: 10.18632/oncotarget.14095. PubMed PMID: 28042950.

106. Cho H, Lim S-J, Won KY, Bae GE, Kim GY, Min JW, et al. Eosinophils in Colorectal Neoplasms Associated with Expression of CCL11 and CCL24. Journal of pathology and translational medicine. 2016;50(1):45-51. Epub 2015/12/14. doi: 10.4132/jptm.2015.10.16. PubMed PMID: 26657310.

107. Alderton GK. T cell opposition. Nature Reviews Cancer. 2011;11:232. doi: 10.1038/nrc3046.

108. Ke C, Ren Y, Lv L, Hu W, Zhou W. Association between CXCL16/CXCR6 expression and the clinicopathological features of patients with non-small cell lung cancer. Oncology letters. 2017;13(6):4661-8. Epub 2017/04/24. doi: 10.3892/ol.2017.6088. PubMed PMID: 28599467.

109. Darash-Yahana M, Gillespie J, Hewitt S, Chen Y-Y, Maeda S, Stein I, et al. CXCL16 and CXCR6 may promote inflammation-associated cancer growth through autocrine effects on cancer cells and paracrine effects on leukocytes. Cancer Research. 2007;67(9 Supplement):5122.

110. Yan T, Lin Z, Jiang J, Lu S, Chen M, Que H, et al. MMP14 regulates cell migration and invasion through epithelial-mesenchymal transition in nasopharyngeal carcinoma. American journal of translational research. 2015;7(5):950-8. PubMed PMID: 26175856.

111. Ling B, Watt K, Banerjee S, Newsted D, Truesdell P, Adams J, et al. A novel immunotherapy targeting MMP-14 limits hypoxia, immune suppression and metastasis in triple-negative breast cancer models. Oncotarget. 2017;8(35):58372-85. doi: 10.18632/oncotarget.17702. PubMed PMID: 28938563.

112. Cathcart JM, Banach A, Liu A, Chen J, Goligorsky M, Cao J. Interleukin-6 increases matrix metalloproteinase-14 (MMP-14) levels via down-regulation of p53 to drive cancer progression. Oncotarget. 2016;7(38):61107-20. doi: 10.18632/oncotarget.11243. PubMed PMID: 27531896.

113. Shirvaikar N, Jalili A, Mirza I, Ilnitsky S, Korol C, Larratt LM, et al. MMP-14 Mediates Migration of Acute Myelogenous Leukemia Cells. Blood. 2008;112(11):2943.

114. Tan X, Chen M. MYLK and MYL9 expression in non-small cell lung cancer identified by bioinformatics analysis of public expression data. Tumour biology : the journal of the International Society for Oncodevelopmental Biology and Medicine. 2014;35(12):12189-200. Epub 2014/09/03. doi: 10.1007/s13277-014-2527-3. PubMed PMID: 25179839.

115. Gurrapu S, Pupo E, Franzolin G, Lanzetti L, Tamagnone L. Sema4C/PlexinB2 signaling controls breast cancer cell growth, hormonal dependence and tumorigenic potential. Cell Death Differ. 2018;25(7):1259-75. Epub 2018/03/21. doi: 10.1038/s41418-018-0097-4. PubMed PMID: 29555978; PubMed Central PMCID: PMCPMC6030176.

116. Wei J-C, Yang J, Liu D, Wu M-F, Qiao L, Wang J-N, et al. Tumor-associated Lymphatic Endothelial Cells Promote Lymphatic Metastasis By Highly Expressing and Secreting SEMA4C. Clinical Cancer Research. 2017;23(1):214. doi: 10.1158/1078-0432.CCR-16-0741.

117. Xu XQ, Huang CM, Zhang YF, Chen L, Cheng H, Wang JM. S1PR1 mediates antiapoptotic/proproliferative processes in human acute myeloid leukemia cells. Molecular medicine reports. 2016;14(4):3369-75. Epub 2016/08/31. doi: 10.3892/mmr.2016.5629. PubMed PMID: 27572094.

118. Vrzalikova K, Ibrahim M, Vockerodt M, Perry T, Margielewska S, Lupino L, et al. S1PR1 drives a feedforward signalling loop to regulate BATF3 and the transcriptional programme of Hodgkin lymphoma cells. Leukemia. 2018;32(1):214-23. Epub 2017/09/08. doi: 10.1038/leu.2017.275. PubMed PMID: 28878352; PubMed Central PMCID: PMCPMC5737877.

119. Weichand B, Popp R, Dziumbla S, Mora J, Strack E, Elwakeel E, et al. S1PR1 on tumor-associated macrophages promotes lymphangiogenesis and metastasis via NLRP3/IL-1beta. J Exp Med. 2017;214(9):2695-713. Epub 2017/07/26. doi: 10.1084/jem.20160392. PubMed PMID: 28739604; PubMed Central PMCID: PMCPMC5584110.

120. Liu Y, Deng J, Wang L, Lee H, Armstrong B, Scuto A, et al. S1PR1 is an effective target to block STAT3 signaling in activated B cell-like diffuse large B-cell lymphoma. Blood. 2012;120(7):1458-65. doi: 10.1182/blood-2011-12-399030. PubMed PMID: 22745305.

121. Patrussi L, Capitani N, Martini V, Pizzi M, Trimarco V, Frezzato F, et al. Enhanced Chemokine Receptor Recycling and Impaired S1P1 Expression Promote Leukemic Cell Infiltration of Lymph Nodes in Chronic Lymphocytic Leukemia. Cancer Res. 2015;75(19):4153-63. Epub 2015/08/19. doi: 10.1158/0008-5472.Can-15-0986. PubMed PMID: 26282174.

122. Paprocka M, Bielawska-Pohl A, Rossowska J, Krawczenko A, Dus D, Kielbinski M, et al. MRP1 protein expression in leukemic stem cells as a negative prognostic marker in acute myeloid leukemia patients. European journal of haematology. 2017;99(5):415-22. Epub 2017/08/15. doi: 10.1111/ejh.12938. PubMed PMID: 28805931.

123. Guimaraes L, Rocha GDG, Queiroz RM, Martins CA, Takiya CM, Gattass CR. Pomolic acid induces apoptosis and inhibits multidrug resistance protein MRP1 and migration in glioblastoma cells. Oncology reports. 2017;38(4):2525-34. Epub 2017/08/30. doi: 10.3892/or.2017.5895. PubMed PMID: 28849227.

124. Liu H, Wu X, Huang J, Peng J, Guo L. miR-7 modulates chemoresistance of small cell lung cancer by repressing MRP1/ABCC1. International journal of experimental pathology. 2015;96(4):240-7. Epub 2015/06/26. doi: 10.1111/iep.12131. PubMed PMID: 26108539; PubMed Central PMCID: PMCPMC4561560.

125. Sedlakova I, Laco J, Caltova K, Cervinka M, Tosner J, Rezac A, et al. Clinical significance of the resistance proteins LRP, Pgp, MRP1, MRP3, and MRP5 in epithelial ovarian cancer. International journal of gynecological cancer : official journal of the International Gynecological Cancer Society. 2015;25(2):236-43. Epub 2015/01/17. doi: 10.1097/igc.0000000000000354. PubMed PMID: 25594141.

126. Abdallah EA, Fanelli MF, Souza ESV, Machado Netto MC, Gasparini Junior JL, Araujo DV, et al. MRP1 expression in CTCs confers resistance to irinotecan-based chemotherapy in metastatic colorectal cancer. International journal of cancer. 2016;139(4):890-8. Epub 2016/03/08. doi: 10.1002/ijc.30082. PubMed PMID: 26950035.

127. Tadmor T, Bejar J, Attias D, Mischenko E, Sabo E, Neufeld G, et al. The expression of lysyl-oxidase gene family members in myeloproliferative neoplasms. Am J Hematol. 2013;88(5):355-8. Epub 2013/03/16. doi: 10.1002/ajh.23409. PubMed PMID: 23494965.

128. Matsuura S, Mi R, Koupenova M, Eliades A, Patterson S, Toselli P, et al. Lysyl oxidase is associated with increased thrombosis and platelet reactivity. Blood. 2016;127(11):1493. doi: 10.1182/blood-2015-02-629667.

129. Shih YH, Chang KW, Chen MY, Yu CC, Lin DJ, Hsia SM, et al. Lysyl oxidase and enhancement of cell proliferation and angiogenesis in oral squamous cell carcinoma. Head & neck. 2013;35(2):250-6. Epub 2012/03/01. doi: 10.1002/hed.22959. PubMed PMID: 22367676.

130. Kirschmann DA, Seftor EA, Fong SF, Nieva DR, Sullivan CM, Edwards EM, et al. A molecular role for lysyl oxidase in breast cancer invasion. Cancer Res. 2002;62(15):4478-83. Epub 2002/08/03. PubMed PMID: 12154058.

131. Kaneda A, Wakazono K, Tsukamoto T, Watanabe N, Yagi Y, Tatematsu M, et al. Lysyl oxidase is a tumor suppressor gene inactivated by methylation and loss of heterozygosity in human gastric cancers. Cancer Res. 2004;64(18):6410-5. Epub 2004/09/18. doi: 10.1158/0008-5472.Can-04-1543. PubMed PMID: 15374948.

132. Bais MV, Ozdener GB, Sonenshein GE, Trackman PC. Effects of tumor-suppressor lysyl oxidase propeptide on prostate cancer xenograft growth and its direct interactions with DNA repair pathways. Oncogene. 2015;34(15):1928-37. Epub 2014/06/03. doi: 10.1038/onc.2014.147. PubMed PMID: 24882580; PubMed Central PMCID: PMCPMC4254378.

133. Zheng Y, Wang X, Wang H, Yan W, Zhang Q, Chang X. Expression of the lysyl oxidase propeptide in hepatocellular carcinoma and its clinical relevance. Oncology reports. 2014;31(4):1669-76. Epub 2014/02/28. doi: 10.3892/or.2014.3044. PubMed PMID: 24573150.

134. Xu X, Wang B, Xu Y. Expression of lysyl oxidase in human osteosarcoma and its clinical significance: a tumor suppressive role of LOX in human osteosarcoma cells. International journal of oncology. 2013;43(5):1578-86. Epub 2013/08/24. doi: 10.3892/ijo.2013.2067. PubMed PMID: 23970168.

135. Zhong M, Boseman ML, Millena AC, Khan SA. Oxytocin induces the migration of prostate cancer cells: involvement of the Gi-coupled signaling pathway. Molecular cancer research : MCR. 2010;8(8):1164-72. Epub 2010/07/27. doi: 10.1158/1541-7786.MCR-09-0329. PubMed PMID: 20663860.

136. Morita T, Shibata K, Kikkawa F, Kajiyama H, Ino K, Mizutani S. Oxytocin inhibits the progression of human ovarian carcinoma cells in vitro and in vivo. International journal of cancer. 2004;109(4):525-32. Epub 2004/03/03. doi: 10.1002/ijc.20017. PubMed PMID: 14991573.

137. Cassoni P, Sapino A, Papotti M, Bussolati G. Oxytocin and oxytocin-analogue F314 inhibit cell proliferation and tumor growth of rat and mouse mammary carcinomas. International journal of cancer. 1996;66(6):817-20. Epub 1996/06/11. doi: 10.1002/(sici)1097-0215(19960611)66:6<817::Aid-ijc18>3.0.Co;2-#. PubMed PMID: 8647655.

138. Cassoni P, Sapino A, Fortunati N, Munaron L, Chini B, Bussolati G. Oxytocin inhibits the proliferation of MDA-MB231 human breast-cancer cells via cyclic adenosine monophosphate and protein kinase A. International journal of cancer. 1997;72(2):340-4. Epub 1997/07/17. PubMed PMID: 9219843.

139. Benavente MA, Bianchi CP, Imperiale F, Aba MA. Antiproliferative Effects of Oxytocin and Desmopressin on Canine Mammary Cancer Cells. Frontiers in veterinary science. 2016;3:119. Epub 2017/01/14. doi: 10.3389/fvets.2016.00119. PubMed PMID: 28083539; PubMed Central PMCID: PMCPMC5183597.

140. Escudero-Esparza A, Martin TA, Davies ML, Jiang WG. PGF isoforms, PLGF-1 and PGF-2, in colorectal cancer and the prognostic significance. Cancer genomics & proteomics. 2009;6(4):239-46. Epub 2009/08/07. PubMed PMID: 19657001.

141. Ikai T, Miwa H, Shikami M, Hiramatsu A, Tajima E, Yamamoto H, et al. Placenta growth factor stimulates the growth of Philadelphia chromosome positive acute lymphoblastic leukemia cells by both autocrine and paracrine pathways. European journal of haematology. 2005;75(4):273-9. Epub 2005/09/09. doi: 10.1111/j.1600-0609.2005.00505.x. PubMed PMID: 16146532.

142. Parr C, Watkins G, Boulton M, Cai J, Jiang WG. Placenta growth factor is over-expressed and has prognostic value in human breast cancer. European journal of cancer (Oxford, England : 1990). 2005;41(18):2819-27. Epub 2005/11/09. doi: 10.1016/j.ejca.2005.07.022. PubMed PMID: 16275058.

143. Raymond A, Liu B, Liang H, Wei C, Guindani M, Lu Y, et al. A role for BMP-induced homeobox gene MIXL1 in acute myelogenous leukemia and identification of type I BMP receptor as a potential target for therapy. Oncotarget. 2014;5(24):12675-93. doi: 10.18632/oncotarget.2564. PubMed PMID: 25544748.

144. Drakos E, Rassidakis GZ, Leventaki V, Guo W, Medeiros LJ, Nagarajan L. Differential expression of the human MIXL1 gene product in non-Hodgkin and Hodgkin lymphomas. Human pathology. 2007;38(3):500-7. Epub 2007/02/17. doi: 10.1016/j.humpath.2006.09.020. PubMed PMID: 17303500.

145. Zudaire E, Martinez A, Cuttitta F. Adrenomedullin and cancer. Regulatory peptides. 2003;112(1-3):175-83. Epub 2003/04/02. PubMed PMID: 12667640.

146. Di Liddo R, Bridi D, Gottardi M, De Angeli S, Grandi C, Tasso A, et al. Adrenomedullin in the growth modulation and differentiation of acute myeloid leukemia cells. International journal of oncology. 2016;48(4):1659-69. Epub 2016/02/06. doi: 10.3892/ijo.2016.3370. PubMed PMID: 26847772.

147. Smollich M, Gotte M, Yip GW, Yong ES, Kersting C, Fischgrabe J, et al. On the role of endothelin-converting enzyme-1 (ECE-1) and neprilysin in human breast cancer. Breast cancer research and treatment. 2007;106(3):361-9. Epub 2007/02/14. doi: 10.1007/s10549-007-9516-9. PubMed PMID: 17295044.

148. Rayhman O, Klipper E, Muller L, Davidson B, Reich R, Meidan R. Small Interfering RNA Molecules Targeting Endothelin-Converting Enzyme-1 Inhibit Endothelin-1 Synthesis and the Invasive Phenotype of Ovarian Carcinoma Cells. Cancer Research. 2008;68(22):9265. doi: 10.1158/0008-5472.CAN-08-2093.

149. Hodson DJ, Janas ML, Galloway A, Bell SE, Andrews S, Li CM, et al. Deletion of the RNA-binding proteins ZFP36L1 and ZFP36L2 leads to perturbed thymic development and T lymphoblastic leukemia. Nat Immunol. 2010;11(8):717-24. Epub 2010/07/14. doi: 10.1038/ni.1901. PubMed PMID: 20622884; PubMed Central PMCID: PMCPMC2953641.

150. Chen MT, Dong L, Zhang XH, Yin XL, Ning HM, Shen C, et al. ZFP36L1 promotes monocyte/macrophage differentiation by repressing CDK6. Sci Rep. 2015;5:16229. Epub 2015/11/07. doi: 10.1038/srep16229. PubMed PMID: 26542173; PubMed Central PMCID: PMCPMC4635361.

151. Zekavati A, Nasir A, Alcaraz A, Aldrovandi M, Marsh P, Norton JD, et al. Post-transcriptional regulation of BCL2 mRNA by the RNA-binding protein ZFP36L1 in malignant B cells. PLoS One. 2014;9(7):e102625. Epub 2014/07/12. doi: 10.1371/journal.pone.0102625. PubMed PMID: 25014217; PubMed Central PMCID: PMCPMC4094554.

152. Loh XY, Ding LW, Koeffler HP. Abstract 4494: Tumor suppressive role of ZFP36L1 by suppressing HIF1α and Cyclin D1 in bladder and breast cancer. Cancer Research. 2017;77(13 Supplement):4494. doi: 10.1158/1538-7445.AM2017-4494.

153. Suk F-M, Chang C-C, Lin R-J, Lin S-Y, Chen Y-T, Liang Y-C. MCPIP3 as a Potential Metastasis Suppressor Gene in Human Colorectal Cancer. International journal of molecular sciences. 2018;19(5):1350. doi: 10.3390/ijms19051350. PubMed PMID: 29751537.

154. Wang Y, He D, Yang L, Wen B, Dai J, Zhang Q, et al. TRIM26 functions as a novel tumor suppressor of hepatocellular carcinoma and its downregulation contributes to worse prognosis. Biochem Biophys Res Commun. 2015;463(3):458-65. Epub 2015/06/06. doi: 10.1016/j.bbrc.2015.05.117. PubMed PMID: 26043685.

155. Yi J, Huang D, Li X, Jiang G, Dong J, Liu Y. TRIM26 acts as a tumor suppressor in non-small-cell lung cancer2016. 6385-90 p.

156. Liu W, Wei H, Gao Z, Chen G, Liu Y, Gao X, et al. COL5A1 may contribute the metastasis of lung adenocarcinoma. Gene. 2018;665:57-66. Epub 2018/04/28. doi: 10.1016/j.gene.2018.04.066. PubMed PMID: 29702185.

157. Ren W, Zhang Y, Zhang L, Lin Q, Zhang J, Xu G. Overexpression of collagen type V alpha1 chain in human breast invasive ductal carcinoma is mediated by TGF-beta1. International journal of oncology. 2018. Epub 2018/03/24. doi: 10.3892/ijo.2018.4317. PubMed PMID: 29568948.

158. Wang J, Yang L, Liang F, Chen Y, Yang G. Integrin alpha x stimulates cancer angiogenesis through PI3K/Akt signaling-mediated VEGFR2/VEGF-A overexpression in blood vessel endothelial cells: WANG et al2018.

159. Williams KA, Lee M, Hu Y, Andreas J, Patel SJ, Zhang S, et al. A systems genetics approach identifies CXCL14, ITGAX, and LPCAT2 as novel aggressive prostate cancer susceptibility genes. PLoS genetics. 2014;10(11):e1004809-e. doi: 10.1371/journal.pgen.1004809. PubMed PMID: 25411967.

160. Wragg JW, Finnity JP, Anderson JA, Ferguson HJM, Porfiri E, Bhatt RI, et al. MCAM and LAMA4 Are Highly Enriched in Tumor Blood Vessels of Renal Cell Carcinoma and Predict Patient Outcome. Cancer research. 2016;76(8):2314-26. Epub 2016/02/26. doi: 10.1158/0008-5472.CAN-15-1364. PubMed PMID: 26921326.

161. Yang ZX, Zhang B, Wei J, Jiang GQ, Wu YL, Leng BJ, et al. MiR-539 inhibits proliferation and migration of triple-negative breast cancer cells by down-regulating LAMA4 expression. Cancer cell international. 2018;18:16. Epub 2018/02/13. doi: 10.1186/s12935-018-0512-4. PubMed PMID: 29434522; PubMed Central PMCID: PMCPMC5791727.

162. Wang X, Hou Q, Zhou X. LAMA4 expression is activated by zinc finger Eboxbinding homeobox 1 and independently predicts poor overall survival in gastric cancer. Oncology reports. 2018;40(3):1725-33. Epub 2018/07/18. doi: 10.3892/or.2018.6564. PubMed PMID: 30015861.

163. Wu N, Kurosu T, Oshikawa G, Nagao T, Miura O. PECAM-1 is involved in BCR/ABL signaling and may downregulate imatinib-induced apoptosis of Philadelphia chromosome-positive leukemia cells. International journal of oncology. 2013;42(2):419-28. Epub 2012/12/13. doi: 10.3892/ijo.2012.1729. PubMed PMID: 23233201; PubMed Central PMCID: PMCPMC3583636.

164. Ploeger C, Waldburger N, Fraas A, Goeppert B, Pusch S, Breuhahn K, et al. Chromosome 8p tumor suppressor genes SH2D4A and SORBS3 cooperate to inhibit interleukin-6 signaling in hepatocellular carcinoma. Hepatology. 2016;64(3):828-42. Epub 2016/06/18. doi: 10.1002/hep.28684. PubMed PMID: 27311882; PubMed Central PMCID: PMCPMC5098049.

165. Caspi M, Perry G, Skalka N, Meisel S, Firsow A, Amit M, et al. Aldolase positively regulates of the canonical Wnt signaling pathway. Molecular cancer. 2014;13:164-. doi: 10.1186/1476-4598-13-164. PubMed PMID: 24993527.

166. Mullican SE, Zhang S, Konopleva M, Ruvolo V, Andreeff M, Milbrandt J, et al. Abrogation of nuclear receptors Nr4a3 and Nr4a1 leads to development of acute myeloid leukemia. Nat Med. 2007;13(6):730-5. Epub 2007/05/23. doi: 10.1038/nm1579. PubMed PMID: 17515897.

167. Ramirez-Herrick AM, Mullican SE, Sheehan AM, Conneely OM. Reduced NR4A gene dosage leads to mixed myelodysplastic/myeloproliferative neoplasms in mice. Blood. 2011;117(9):2681-90. Epub 2011/01/06. doi: 10.1182/blood-2010-02-267906. PubMed PMID: 21205929; PubMed Central PMCID: PMCPMC3062356.

168. Wenzl K, Troppan K, Neumeister P, Deutsch AJ. The nuclear orphan receptor NR4A1 and NR4A3 as tumor suppressors in hematologic neoplasms. Current drug targets. 2015;16(1):38-46. Epub 2014/11/21. PubMed PMID: 25410408.

169. Deutsch AJ, Rinner B, Wenzl K, Pichler M, Troppan K, Steinbauer E, et al. NR4A1-mediated apoptosis suppresses lymphomagenesis and is associated with a favorable cancer-specific survival in patients with aggressive B-cell lymphomas. Blood. 2014;123(15):2367-77. Epub 2014/02/21. doi: 10.1182/blood-2013-08-518878. PubMed PMID: 24553175.

170. Wu H, Bi J, Peng Y, Huo L, Yu X, Yang Z, et al. Nuclear receptor NR4A1 is a tumor suppressor down-regulated in triple-negative breast cancer. Oncotarget. 2017;8(33):54364-77. doi: 10.18632/oncotarget.17532. PubMed PMID: 28903348.

171. Yan H, Xiao F, Zou J, Qiu C, Sun W, Gu M, et al. NR4A1-induced increase in the sensitivity of a human gastric cancer line to TNFα-mediated apoptosis is associated with the inhibition of JNK/Parkin-dependent mitophagy. International journal of oncology. 2017;52(2):367-78. doi: 10.3892/ijo.2017.4216. PubMed PMID: 29207128.

172. Zhu B, Yang J-R, Jia Y, Zhang P, Shen L, Li X-L, et al. Overexpression of NR4A1 is associated with tumor recurrence and poor survival in non-small-cell lung carcinoma. Oncotarget. 2017;8(69):113977-86. doi: 10.18632/oncotarget.23048. PubMed PMID: 29371962.

173. Feng M, Yao G, Yu H, Qing Y, Wang K. Tumor apelin, not serum apelin, is associated with the clinical features and prognosis of gastric cancer. BMC Cancer. 2016;16(1):794. Epub 2016/10/14. doi: 10.1186/s12885-016-2815-y. PubMed PMID: 27733135; PubMed Central PMCID: PMCPMC5062883.

174. Berta J, Hoda MA, Laszlo V, Rozsas A, Garay T, Torok S, et al. Apelin promotes lymphangiogenesis and lymph node metastasis. Oncotarget. 2014;5(12):4426-37. doi: 10.18632/oncotarget.2032. PubMed PMID: 24962866.

175. Yu S-T, Zhong Q, Chen R-H, Han P, Li S-B, Zhang H, et al. CRLF1 promotes malignant phenotypes of papillary thyroid carcinoma by activating the MAPK/ERK and PI3K/AKT pathways. Cell death & disease. 2018;9(3):371-. doi: 10.1038/s41419-018-0352-0. PubMed PMID: 29515111.

176. Huusko P, Ponciano-Jackson D, Wolf M, Kiefer JA, Azorsa DO, Tuzmen S, et al. Nonsense-mediated decay microarray analysis identifies mutations of EPHB2 in human prostate cancer. Nat Genet. 2004;36(9):979-83. Epub 2004/08/10. doi: 10.1038/ng1408. PubMed PMID: 15300251.

177. Jubb AM, Zhong F, Bheddah S, Grabsch HI, Frantz GD, Mueller W, et al. EphB2 is a prognostic factor in colorectal cancer. Clin Cancer Res. 2005;11(14):5181-7. Epub 2005/07/22. doi: 10.1158/1078-0432.Ccr-05-0143. PubMed PMID: 16033834.

178. Guo DL, Zhang J, Yuen ST, Tsui WY, Chan AS, Ho C, et al. Reduced expression of EphB2 that parallels invasion and metastasis in colorectal tumours. Carcinogenesis. 2006;27(3):454-64. Epub 2005/11/08. doi: 10.1093/carcin/bgi259. PubMed PMID: 16272170.

179. Gao Q, Liu W, Cai J, Li M, Gao Y, Lin W, et al. EphB2 promotes cervical cancer progression by inducing epithelial-mesenchymal transition2014. 372-81 p.

180. Jang I, Jeon BT, Jeong EA, Kim E-J, Kang D, Lee JS, et al. Pak1/LIMK1/Cofilin Pathway Contributes to Tumor Migration and Invasion in Human Non-Small Cell Lung Carcinomas and Cell Lines. The Korean journal of physiology & pharmacology : official journal of the Korean Physiological Society and the Korean Society of Pharmacology. 2012;16(3):159-65. Epub 2012/06/26. doi: 10.4196/kjpp.2012.16.3.159. PubMed PMID: 22802696.

181. Vlecken DH, Bagowski CP. LIMK1 and LIMK2 are important for metastatic behavior and tumor cell-induced angiogenesis of pancreatic cancer cells. Zebrafish. 2009;6(4):433-9. Epub 2010/01/06. doi: 10.1089/zeb.2009.0602. PubMed PMID: 20047470.

182. You T, Gao W, Wei J, Jin X, Zhao Z, Wang C, et al. Overexpression of LIMK1 promotes tumor growth and metastasis in gastric cancer. Biomed Pharmacother. 2015;69:96-101. doi: 10.1016/j.biopha.2014.11.011. PubMed PMID: 25661344.

183. Zhang Y, Li A, Shi J, Fang Y, Gu C, Cai J, et al. Imbalanced LIMK1 and LIMK2 expression leads to human colorectal cancer progression and metastasis via promoting β-catenin nuclear translocation. Cell Death & Disease. 2018;9(7):749. doi: 10.1038/s41419-018-0766-8.

184. Su J, Zhou Y, Pan Z, Shi L, Yang J, Liao A, et al. Downregulation of LIMK1-ADF/cofilin by DADS inhibits the migration and invasion of colon cancer. Scientific reports. 2017;7:45624-. doi: 10.1038/srep45624. PubMed PMID: 28358024.

185. Pandey R, Mali R, Chatterjee A, Paublant F, Prudent R, Lafanechere L, et al. Role of LIM Kinase in Oncogenic Signaling from FLT3 and KIT Receptors and Its Targeting in Myeloid Leukemia. Blood. 2015;126(23):1257.

186. Antonipillai J, Francis S. Inhibition of LIM kinase inhibits cancer growth2018.

187. Daulat AM, Bertucci F, Audebert S, Serge A, Finetti P, Josselin E, et al. PRICKLE1 Contributes to Cancer Cell Dissemination through Its Interaction with mTORC2. Dev Cell. 2016;37(4):311-25. Epub 2016/05/18. doi: 10.1016/j.devcel.2016.04.011. PubMed PMID: 27184734.

188. Nicke B, Bastien J, Khanna SJ, Warne PH, Cowling V, Cook SJ, et al. Involvement of MINK, a Ste20 Family Kinase, in Ras Oncogene-Induced Growth Arrest in Human Ovarian Surface Epithelial Cells. Molecular Cell. 2005;20(5):673-85. doi: <https://doi.org/10.1016/j.molcel.2005.10.038>.

189. Semenas J, Hedblom A, Miftakhova RR, Sarwar M, Larsson R, Shcherbina L, et al. The role of PI3K/AKT-related PIP5K1α and the discovery of its selective inhibitor for treatment of advanced prostate cancer. Proceedings of the National Academy of Sciences. 2014;111(35):E3689. doi: 10.1073/pnas.1405801111.

190. Sarwar M, Syed Khaja AS, Aleskandarany M, Karlsson R, Althobiti M, Ødum N, et al. The role of PIP5K1α/pAKT and targeted inhibition of growth of subtypes of breast cancer using PIP5K1α inhibitor. Oncogene. 2018. doi: 10.1038/s41388-018-0438-2.
