# Supplementary material for: IKAROS is required for the measured response of NOTCH target genes upon external NOTCH signaling
Source: PLoS Genet. 2021 Mar 26;17(3):e1009478. doi: 10.1371/journal.pgen.1009478 (PMC8026084; doi:10.1371/journal.pgen.1009478)
Supplement: S8 Table — (DOCX) [file pgen.1009478.s008.docx]

**Table S8**. Tumor suppressors and oncogenes included in the list of the 21 genes characterized by the ‘desensitize effect’ (see Fig 2B).

| Protein | Details if needed | Tumor suppressor | Oncogene | Tissus |
| --- | --- | --- | --- | --- |
| LRIG1 |  | X |  | Malignant glioma [1], duodenal adenomas [2], breast cancer [3], EGFR-mutant NSCLC [4] |
| NRARP |  |  | X | Thyroid cancer [5]; breast cancer [6], NSCLC [7] |
| PRDM16 | short isoform |  | X | Prostate cancer [8], AML [9] |
|  | long isoform | X |  | Renal cell carcinoma [10], MLL-AF9 Leukemic Transformation [11] |
| PADI-2 |  |  | X | Prostate cancer [12], breast cancer [13], skin neoplasia [14], multiple myeloma [15] |
| GPR37 |  |  | X | Gastric cancer [16], multiple myeloma [17] |
|  |  | X |  | Hepatocellular carcinoma [18] |
| NKAPL |  | X |  | Hepatocellular carcinoma [19] |
| B4GALNT3 |  | X |  | Neuroblastoma [20] |
|  |  |  | X | Colorectal cancer [21], papillary thyroid carcinoma [22] |

**REFERENCES:**

1. Mao F, Holmlund C, Faraz M, Wang W, Bergenheim T, Kvarnbrink S, et al. Lrig1 is a haploinsufficient tumor suppressor gene in malignant glioma. Oncogenesis. 2018;7(2):13-. doi: 10.1038/s41389-017-0012-8. PubMed PMID: 29391393.

2. Powell AE, Wang Y, Li Y, Poulin EJ, Means AL, Washington MK, et al. The pan-ErbB negative regulator Lrig1 is an intestinal stem cell marker that functions as a tumor suppressor. Cell. 2012;149(1):146-58. Epub 2012/04/03. doi: 10.1016/j.cell.2012.02.042. PubMed PMID: 22464327; PubMed Central PMCID: PMCPMC3563328.

3. Krig SR, Frietze S, Simion C, Miller JK, Fry WHD, Rafidi H, et al. Lrig1 Is an Estrogen-Regulated Growth Suppressor and Correlates with Longer Relapse-Free Survival in ERα-Positive Breast Cancer. Molecular Cancer Research. 2011;9(10):1406. doi: 10.1158/1541-7786.MCR-11-0227.

4. Torigoe H, Yamamoto H, Sakaguchi M, Youyi C, Namba K, Sato H, et al. Tumor-suppressive effect of LRIG1, a negative regulator of ErbB, in non-small cell lung cancer harboring mutant EGFR. Carcinogenesis. 2018;39(5):719-27. Epub 2018/03/17. doi: 10.1093/carcin/bgy044. PubMed PMID: 29546323.

5. Chu BF, Qin YY, Zhang SL, Quan ZW, Zhang MD, Bi JW. Downregulation of Notch-regulated Ankyrin Repeat Protein Exerts Antitumor Activities against Growth of Thyroid Cancer. Chinese medical journal. 2016;129(13):1544-52. Epub 2016/07/02. doi: 10.4103/0366-6999.184465. PubMed PMID: 27364790; PubMed Central PMCID: PMCPMC4931260.

6. Imaoka T, Okutani T, Daino K, Iizuka D, Nishimura M, Shimada Y. Overexpression of NOTCH-regulated ankyrin repeat protein is associated with breast cancer cell proliferation. Anticancer research. 2014;34(5):2165-71. Epub 2014/04/30. PubMed PMID: 24778018.

7. Liao Y, Chen J, Ma J, Mao Q, Wei R, Zheng J. Notch-regulated ankyrin-repeat protein is a novel tissue biomarker that predicts poor prognosis in non-small cell lung cancer. Oncology letters. 2018;16(2):1885-91. Epub 2018/05/30. doi: 10.3892/ol.2018.8826. PubMed PMID: 30008880.

8. Zhu S, Xu Y, Song M, Chen G, Wang H, Zhao Y, et al. PRDM16 is associated with evasion of apoptosis by prostatic cancer cells according to RNA interference screening. Molecular medicine reports. 2016;14(4):3357-61. Epub 2016/08/12. doi: 10.3892/mmr.2016.5605. PubMed PMID: 27511603.

9. Yamato G, Yamaguchi H, Handa H, Shiba N, Kawamura M, Wakita S, et al. Clinical features and prognostic impact of PRDM16 expression in adult acute myeloid leukemia. 2017;56(11):800-9. doi: doi:10.1002/gcc.22483.

10. Kundu A, Kho E-Y, Shelar SB, Nam H, Brinkley G, Darshan S, et al. Abstract 4483: Functional implications of &lt;em&gt;PRDM16&lt;/em&gt; loss in kidney cancer. Cancer Research. 2018;78(13 Supplement):4483. doi: 10.1158/1538-7445.AM2018-4483.

11. Zhou B, Wang J, Lee SY, Xiong J, Bhanu N, Guo Q, et al. PRDM16 Suppresses MLL1r Leukemia via Intrinsic Histone Methyltransferase Activity. Molecular cell. 2016;62(2):222-36. Epub 2016/04/14. doi: 10.1016/j.molcel.2016.03.010. PubMed PMID: 27151440.

12. Wang L, Song G, Zhang X, Feng T, Pan J, Chen W, et al. PADI2-Mediated Citrullination Promotes Prostate Cancer Progression. Cancer Res. 2017;77(21):5755-68. Epub 2017/08/19. doi: 10.1158/0008-5472.Can-17-0150. PubMed PMID: 28819028.

13. McElwee J, Mohanan S, Griffith O, Breuer H, Anguish L, Cherrington B, et al. Identification of PADI2 as a potential breast cancer biomarker and therapeutic target2012. 500 p.

14. McElwee JL, Mohanan S, Horibata S, Sams KL, Anguish LJ, McLean D, et al. PAD2 overexpression in transgenic mice promotes spontaneous skin neoplasia. Cancer Res. 2014;74(21):6306-17. Epub 2014/09/13. doi: 10.1158/0008-5472.Can-14-0749. PubMed PMID: 25213324.

15. McNee G, Eales KL, Wei W, Williams DS, Barkhuizen A, Bartlett DB, et al. Citrullination of histone H3 drives IL-6 production by bone marrow mesenchymal stem cells in MGUS and multiple myeloma. Leukemia. 2016;31:373. doi: 10.1038/leu.2016.187

<https://www.nature.com/articles/leu2016187#supplementary-information>.

16. Wang H, Hu L, Zang M, Zhang B, Duan Y, Fan Z, et al. REG4 promotes peritoneal metastasis of gastric cancer through GPR37. Oncotarget. 2016;7(19):27874-88. doi: 10.18632/oncotarget.8442. PubMed PMID: 27036049.

17. Huang X, Wang Y, Nan X, He S, Xu X, Zhu X, et al. The role of the orphan G protein-coupled receptor 37 (GPR37) in multiple myeloma cells2013.

18. Liu F, Zhu C, Huang X, Cai J, Wang H, Wang X, et al. A low level of GPR37 is associated with human hepatocellular carcinoma progression and poor patient survival. Pathology, research and practice. 2014;210(12):885-92. Epub 2014/08/30. doi: 10.1016/j.prp.2014.07.011. PubMed PMID: 25169131.

19. Ng PKS, Lau CPY, Lam EKY, Li SSK, Lui VWY, Yeo W, et al. Hypermethylation of NF-κB-Activating Protein-Like (NKAPL) Promoter in Hepatocellular Carcinoma Suppresses Its Expression and Predicts a Poor Prognosis. Dig Dis Sci. 2018;63(3):676-86. doi: 10.1007/s10620-018-4929-3. PubMed PMID: 29353445.

20. Hsu W-M, Che M-I, Liao Y-F, Chang H-H, Chen C-H, Huang Y-M, et al. B4GALNT3 expression predicts a favorable prognosis and suppresses cell migration and invasion via β₁ integrin signaling in neuroblastoma. The American journal of pathology. 2011;179(3):1394-404. doi: 10.1016/j.ajpath.2011.05.025. PubMed PMID: 21741930.

21. Che M-I, Huang J, Hung J-S, Lin Y-C, Huang M-J, Lai H-S, et al. β1, 4-N-acetylgalactosaminyltransferase III modulates cancer stemness through EGFR signaling pathway in colon cancer cells. Oncotarget. 2014;5(11):3673-84. doi: 10.18632/oncotarget.1981. PubMed PMID: 25003232.

22. Costa V, Esposito R, Ziviello C, Sepe R, Bim LV, Cacciola NA, et al. New somatic mutations and WNK1-B4GALNT3 gene fusion in papillary thyroid carcinoma. Oncotarget. 2015;6(13):11242-51. Epub 2015/03/25. doi: 10.18632/oncotarget.3593. PubMed PMID: 25803323; PubMed Central PMCID: PMCPMC4484453.
