# Supplementary material for: IKAROS is required for the measured response of NOTCH target genes upon external NOTCH signaling
Source: PLoS Genet. 2021 Mar 26;17(3):e1009478. doi: 10.1371/journal.pgen.1009478 (PMC8026084; doi:10.1371/journal.pgen.1009478)
Supplement: S9 Table — (DOCX) [file pgen.1009478.s009.docx]

**Table S9**. List of the 223 genes characterized by the ‘additive effect’ (see Fig 2A).

| Smtnl2 | Serinc2 | S1pr1 | Gm6548 | Mnda | Entpd1 |
| --- | --- | --- | --- | --- | --- |
| Mocs1 | Rasgef1b | Gm15471 | Cdc42ep2 | Gbp9 | Efnb3 |
| Ttc23 | Tmem27 | H2-Q2 | AC142191.1 | Ephb2 | Csf1 |
| Slc25a44 | Adamts3 | Atp1b2 | C1ra | Deptor | Usp42 |
| Myo15 | Pisd-ps2 | B2m | Sh3rf1 | Trim26 | Adora3 |
| Cdh5 | Serpinb6a | Aqp9 | Clec2d | Disp2 | Adar |
| Fam65c | Aldh1a3 | Mixl1 | Ahr | Tor1aip1 | AC155814.1 |
| Cd97 | Hexim2 | Arf4 | Irf9 | Gas6 | C1qc |
| Gpr124 | Gltp | Slc5a3 | Gm11837 | Ccbe1 | Dlc1 |
| Fam160a1 | Itgax | 1700012L04Rik | Dcaf11 | 4930563E22Rik | Nudt13 |
| Maged1 | Ccl24 | Tmem86a | Psme2 | Tgif1 | Apln |
| Ap5s1 | Gm7592 | Luzp1 | AC124336.1 | Gapdh | Ankfy1 |
| Abcc1 | Mfap3l | Mdm2 | Ifi35 | Mcpt4 | Zfp334 |
| Ptger4 | Serpina3h | Zfp446 | Rnf31 | Snora28 | Kcnj12 |
| Dync1h1 | Wdr19 | C1qa | Nr4a1 | Smad6 | Mpzl3 |
| Mmp14 | Slc26a2 | Eif2ak2 | Tgfb1i1 | C920025E04Rik | Lama4 |
| Cox6b2 | Mink1 | Adra2b | Psme2b-ps | 9130020K20Rik | Spata31d1d |
| H2-M2 | Suv420h2 | Cd38 | Cdkn1a | Oxtr | Gm10651 |
| Mylk | Snai1 | AC164093.1 | Tnks1bp1 | AC154274.1 | H2-T24 |
| Glt28d2 | Med12l | Vps33b | Chd3 | Soga1 | Gm15920 |
| Ecm1 | Traf5 | AW011738 | Cdhr4 | Limk1 | Axl |
| Dnahc1 | Zfp595 | AC162692.1 | Npl | 2310042D19Rik | Doc2g |
| Mafb | Wdr91 | Zfp953 | Sorbs3 | Mustn1 | Pilra |
| Tmem47 | Itga5 | Cxcl16 | Zfp58 | Zfp112 | Sema4c |
| Pdzk1ip1 | Mtcp1 | Gm12522 | Kctd20 | Serpinb9 | Trim12c |
| Radil | Pip5k1a | Ak1 | Tnfrsf23 | Sytl5 | Aspa |
| Mmp13 | Ms4a7 | Pmepa1 | Hyal1 | Gm14005 | 4933433C11Rik |
| Chd5 | Nudt8 | Crtc1 | Cd300a | Notch3 | Zcchc2 |
| Sept1 | Pecam1 | Sgk1 | Col1a2 | Cdc34-ps | Pnpt1 |
| Tp53 | Ptk2b | C130050O18Rik | Cc2d2a | Pgf | Il6 |
| Ece1 | Acacb | St5 | Stac2 | Nod1 | Phf11c |
| Zfp36l1 | Cdc42ep5 | Tgif2 | AI606181 | Lox | Nsmaf |
| Sox12 | D630023F18Rik | Gpatch11 | Rbfox2 | Fam118b | A730061H03Rik |
| Fyco1 | Crlf1 | Aldoc | Nfkbie | Lair1 | Cd4 |
| Tenc1 | Lbh | Ogfr | Stxbp1 | Gm3695 | Zfp101 |
| Pls3 | Ly6c1 | Pyroxd2 | Il17rc | Ggt5 | 9830147E19Rik |
| Adm | Dzip1l | Col5a1 | Ppa1 | Gm13086 | A730011C13Rik |
| B230307C23Rik |  |  |  |  |  |
