# Supplementary figures and images for: IKAROS is required for the measured response of NOTCH target genes upon external NOTCH signaling
Source: PLoS Genet. 2021 Mar 26;17(3):e1009478. doi: 10.1371/journal.pgen.1009478 (PMC8026084; doi:10.1371/journal.pgen.1009478)

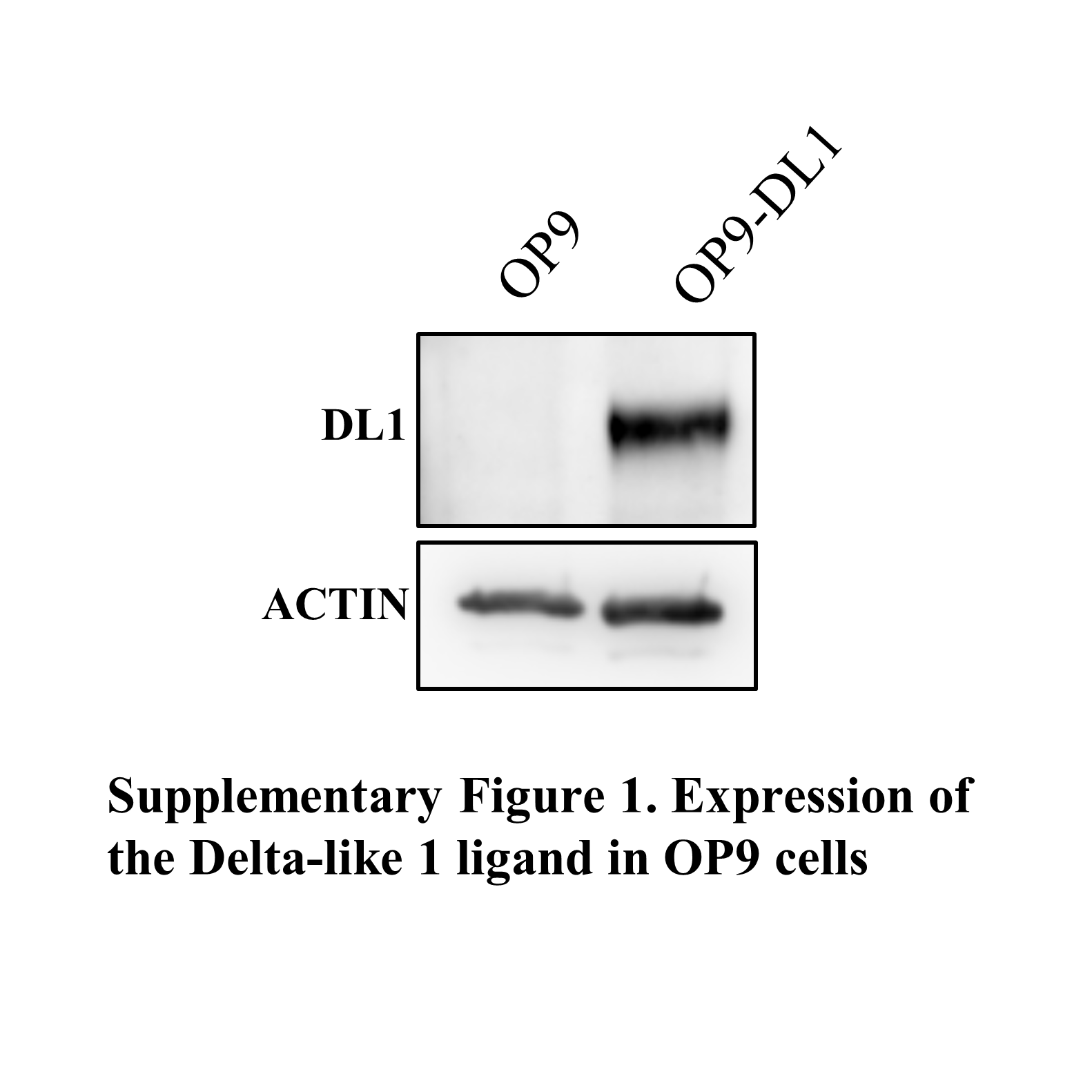

Supplement: S1 Fig — Western blot analysis of Delta-like 1 ligand (DL1) and ACTIN (loading control) expression in OP9 or OP9-DL1 total cell lysates. (TIF) [file pgen.1009478.s010.tif]

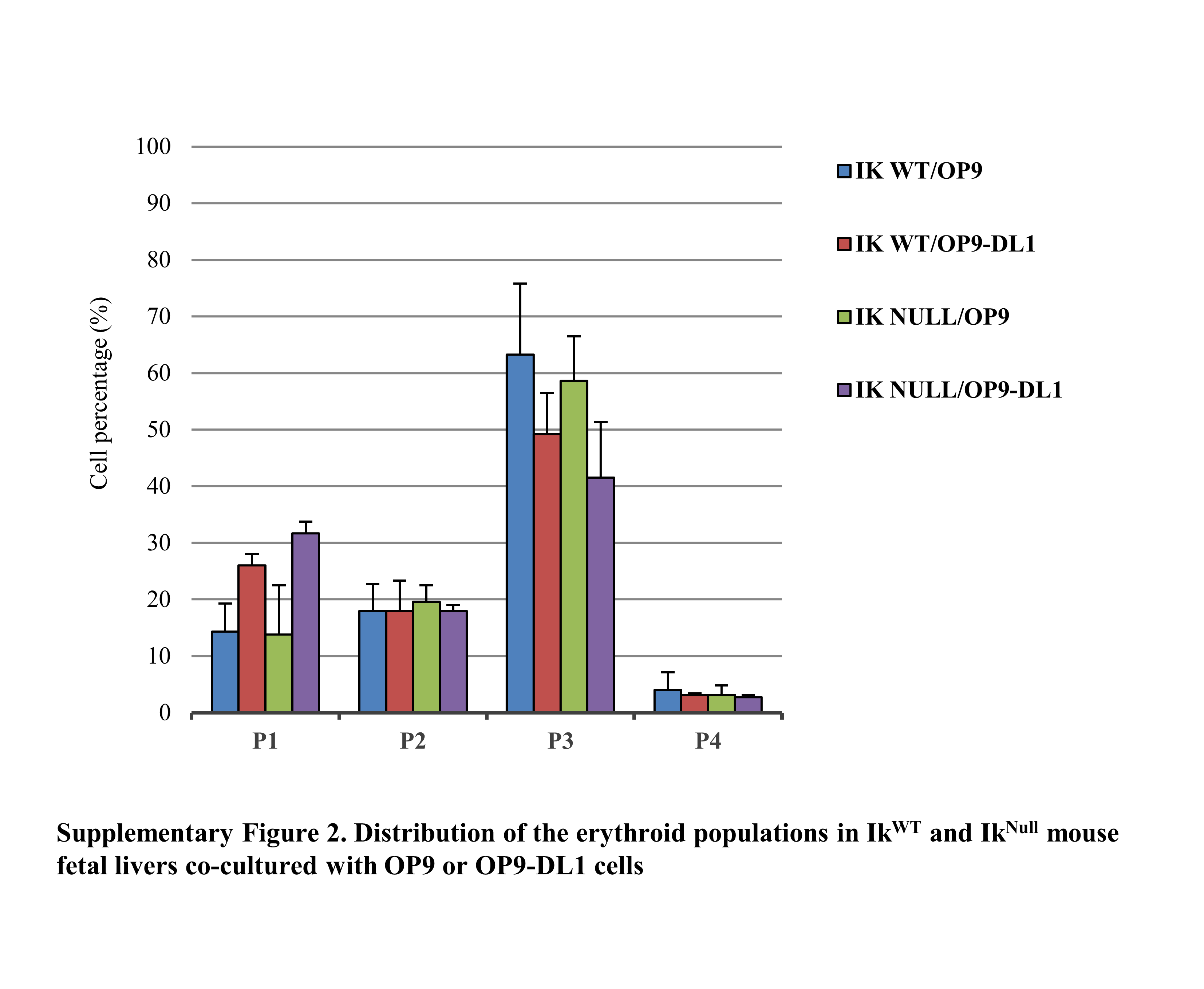

Supplement: S2 Fig — IkNull and IkWT e14.5 fetal livers were isolated and single-cell suspensions were co-cultured with OP9 or OP9-DL1 cells; cells were collected after 48h and analyzed by flow cytometry on the basis of Ter119 and CD71 expression levels: P1, CD71med/Ter119neg/low; P2, CD71high/Ter119neg/low; P3, CD71high/Ter119high; P4, CD71med/Ter119high; P1 is enriched in erythroid precursors (BFU-E: burst-forming unit-erythroid and CFU-E: colony-forming unit erythroid), P2 is enriched in proerythroblasts and early basophilic erythroblasts, P3 is enriched in basophilic and chromatophilic erythroblasts, and P4 is enriched in orthochromatic erythroblasts; x-axis: cell populations; y-axis: percentage of positive cells. (TIF) [file pgen.1009478.s011.tif]

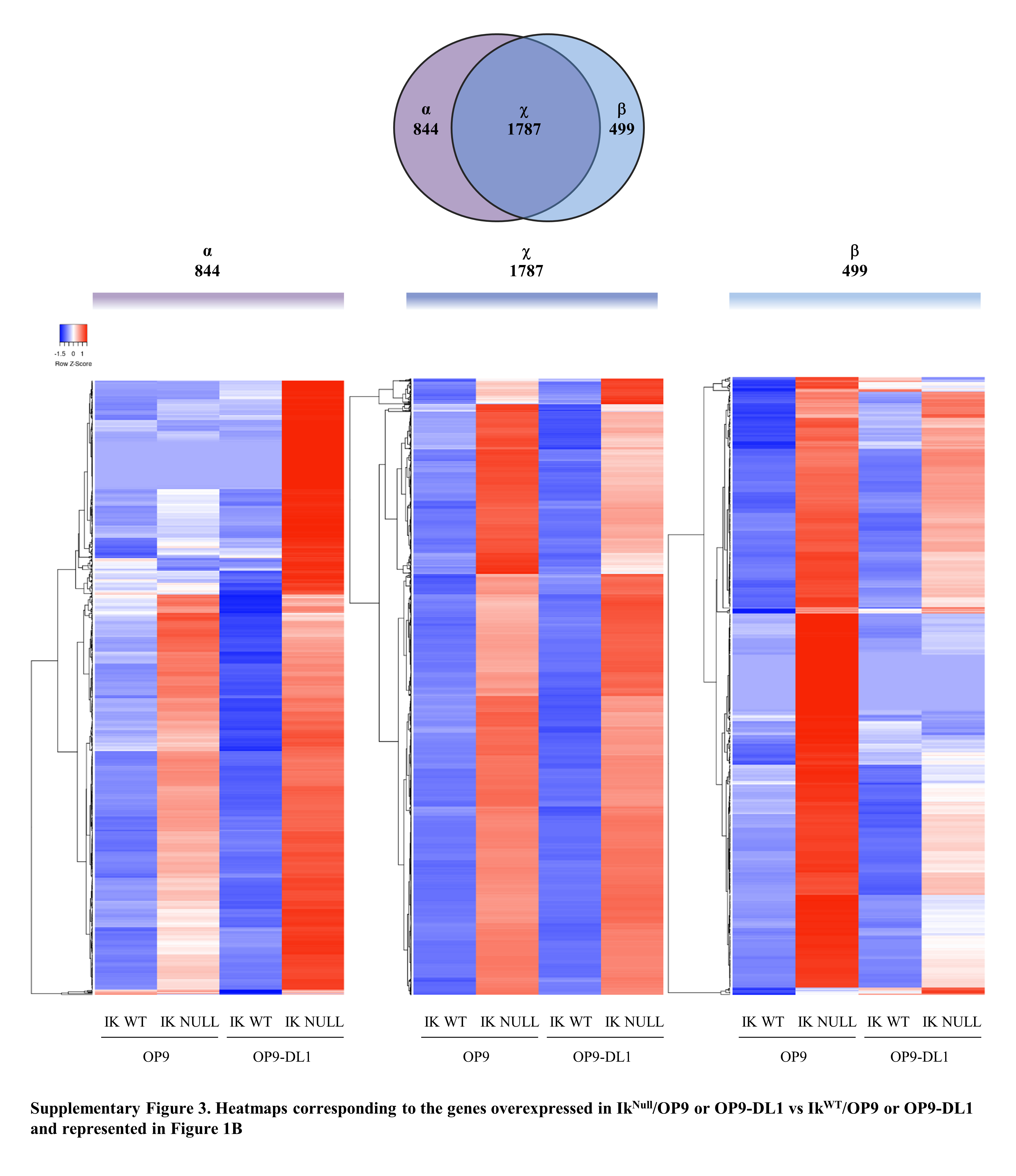

Supplement: S3 Fig — These heatmaps are complementary to Fig 1B. Colors on the heatmaps (depicted in the color key) represent the relative expression levels of individual genes in the four conditions used for this study (indicated at the bottom of the heatmaps). Unsupervised hierarchical clustering (Pearson correlation, average linkage) over the genes included in each group is presented on the left side of the heatmaps. (TIF) [file pgen.1009478.s012.tif]

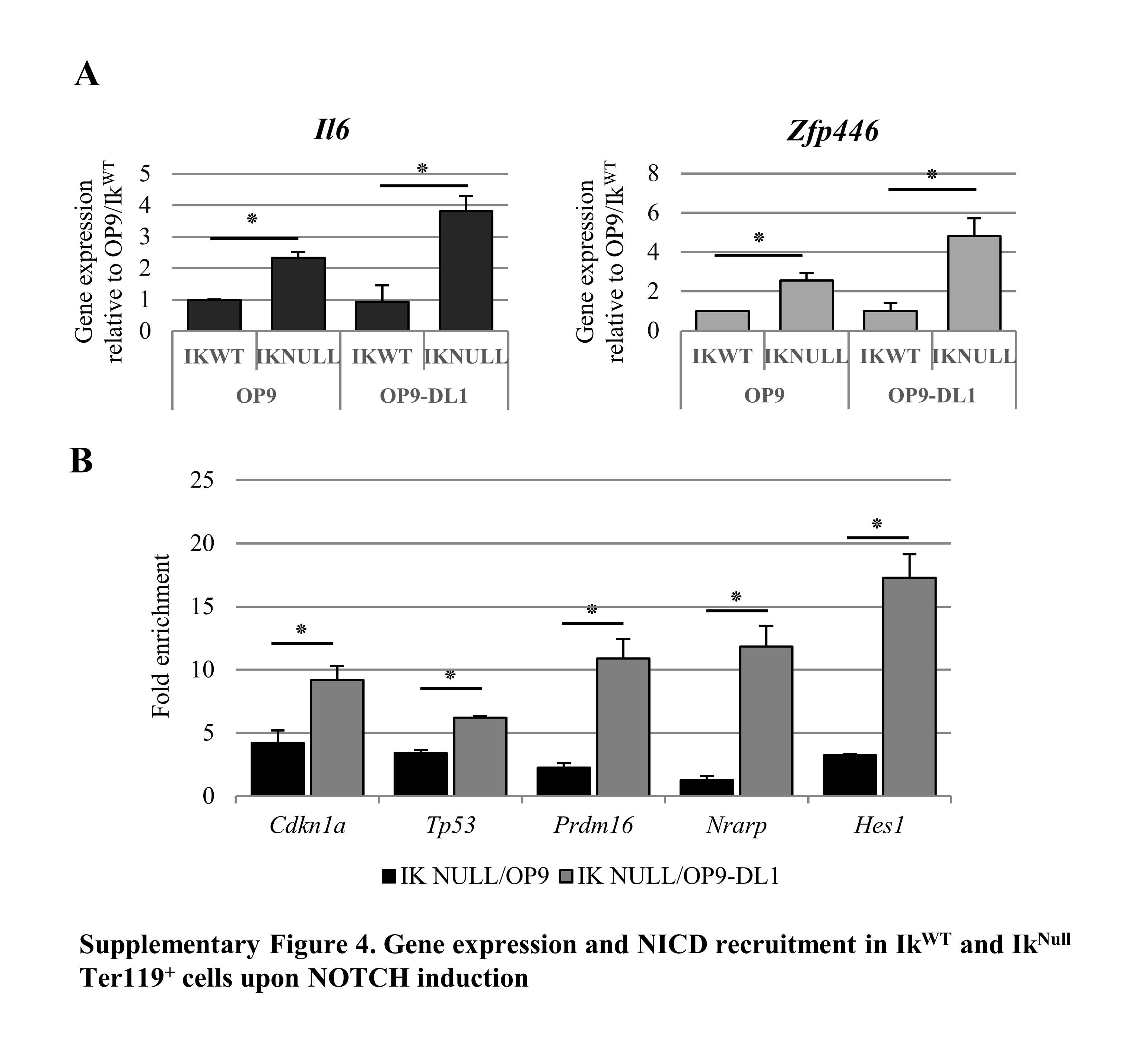

Supplement: S4 Fig — (A) Gene expression; the relative expression levels of Il6 and Zfp446 genes were measured by qRT-PCR, calculated according to the Pfaffl equation using Hprt as internal control and normalized to IkWT/OP9; y axis: relative RNA enrichment levels, ratios are represented by bars and are plotted as the mean ± Standard Deviation (SD) of the measurements; data shown are the results of three independent experiments; *: p ≤ 0.05 by Student’s t-test. (B) Chromatin immunoprecipitation assays in IkNull/OP9 or IkWT/OP9-DL1 cell with NICD antibodies; immunoprecipitated and unbound (input) chromatin samples were used as templates in qPCR analysis with primers specific for Cdkn1a, Tp53, Prmd16, Nrarp or Hes1 transcription start site (TSS) regions; y-axis: fold enrichment levels calculated according to the Pfaffl equation using the Thp1 promoter region as internal control, are represented by bars and plotted as the mean ± SD of the measurements; a value of 1 indicates no enrichment; data shown are the results of three independent experiments; TSS: transcription start site; *: p ≤ 0.05 by Student’s t-test. (TIF) [file pgen.1009478.s013.tif]

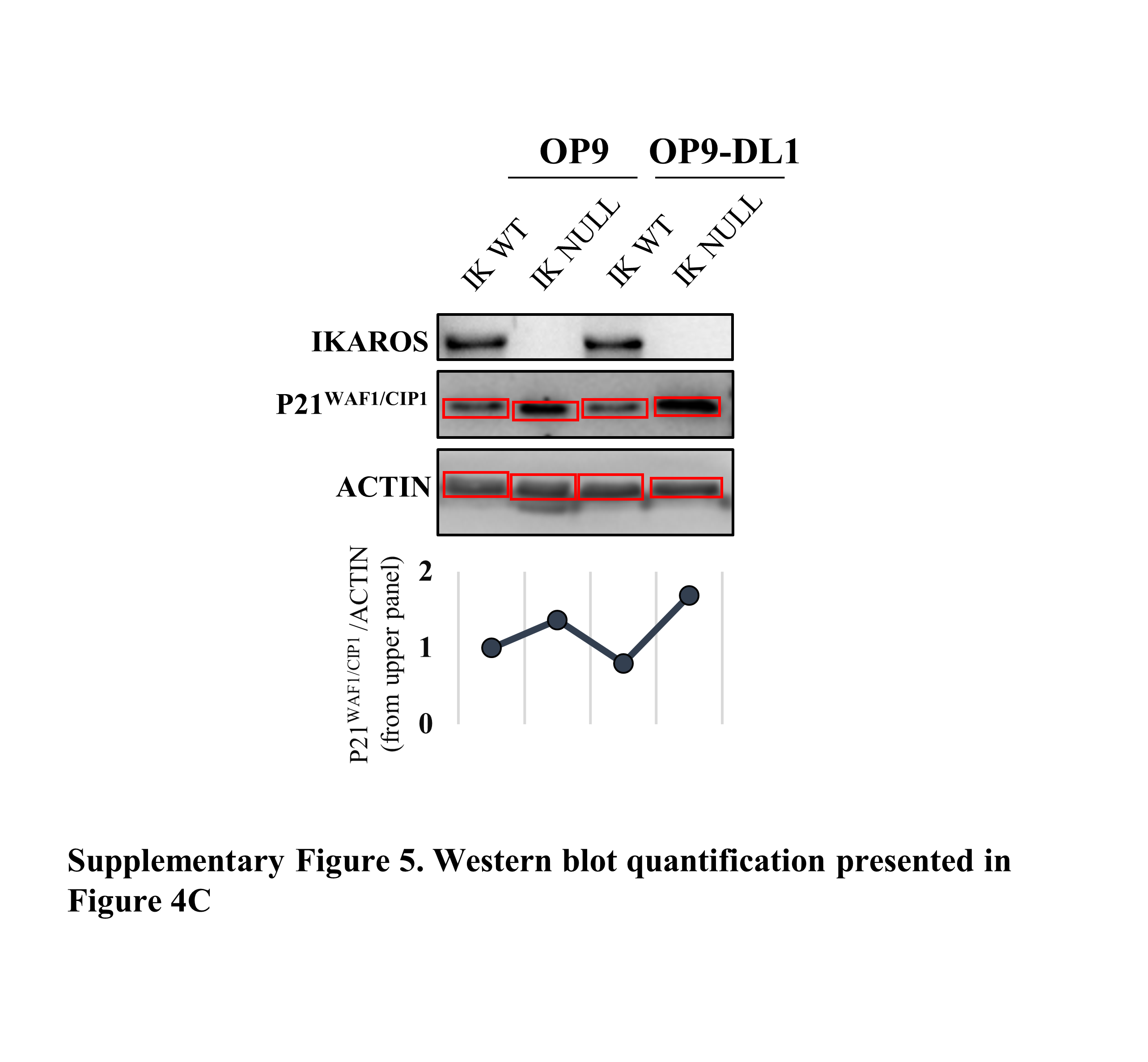

Supplement: S5 Fig — This figure provides further information regarding the quantification of the Western blot results presented in Fig 4C. The red boxes indicate selected areas subject to quantification. After background subtraction, the ratio of P21WAF1/CIP1/ACTIN was calculated and depicted as graph (bottom part of Fig 4C). The same procedure was used for the quantification of the Western blot results presented in Fig 4D. (TIF) [file pgen.1009478.s014.tif]

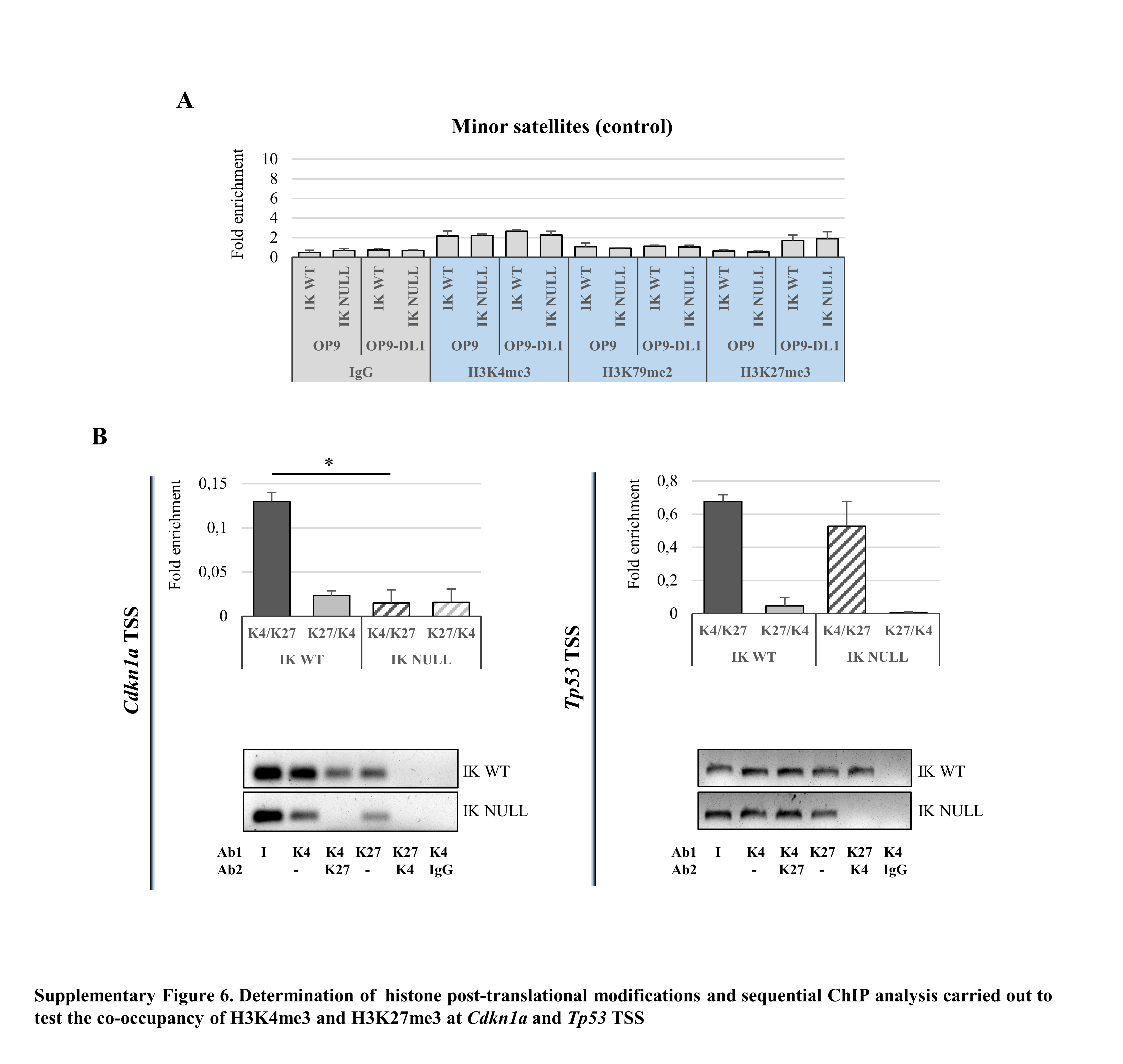

Supplement: S6 Fig — (A) ChIP assays with H3K4me3, H3K79me2, H3K27me3 antibodies (blue) or isotype-matched IgG (grey) were conducted in IkWT or IkNull Ter119+ cells co-cultured with OP9 or OP9-DL1 cells; immunoprecipitated and unbound (input) chromatin samples were used as templates in qPCR analyses with primers specific for the minor satellite repeat sequence, which is used as the control region for the ChIP results presented in Fig 5; y-axis: fold enrichment levels calculated according to the Pfaffl equation using Thp1 promoter region as internal control, are represented by bars and plotted as the mean ± Standard Deviation of the measurements; a value of 1 indicates no enrichment; data shown are the results of three independent experiments. (B) Sequential ChIP (re-ChIP) assays carried out on erythroid cells isolated from e14.5 IkWT or IkNull fetal livers with antibodies directed against H3K4me3, H3K27me3 or isotype-matched IgG. K4/K27 bars: H3K4me3 antibodies were used for the first round of precipitation and H3K27me3 antibodies for the second ChIP; K27/K4 bars: H3K27me3 antibodies were used for the first round of precipitation and H3K4me3 antibodies for the second ChIP. Top panels: immunoprecipitated and unbound (input, I) chromatin samples were used as templates in qPCR analysis with primers specific for Cdkn1a or Tp53 TSS; y-axis: fold enrichment levels calculated according to the “percentage input method” (https://www.thermofisher.com/ca/en/home/life-science/epigenetics-noncoding-rnaresearch/chromatin-remodeling/chromatin-immunoprecipitation-chip/chip-analysis.html) are represented by bars and plotted as the mean ± Standard Deviation of the measurements; in the re-ChIP samples, no amplification for Thp1 promoter region (used as internal control) could be observed; similarly, no amplification for Cdkn1a or Tp53 TSS regions was observed when isotype-matched IgG were used for the second round of precipitation; data shown are the results of three independent experiments; *: p ≤ 0.05 b [file pgen.1009478.s015.tif]

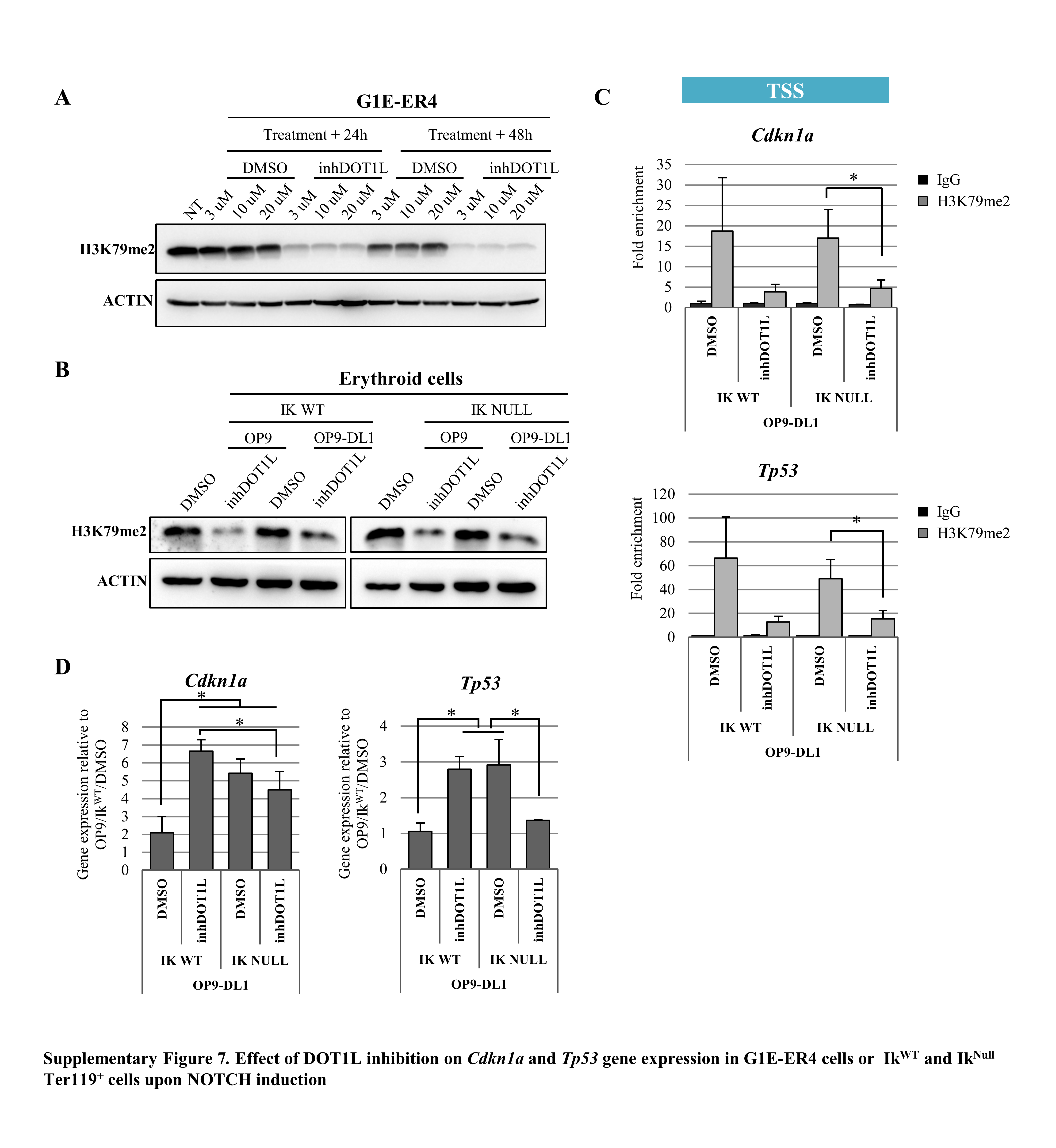

Supplement: S7 Fig — (A) Western blot analysis of H3K79me2 and ACTIN (loading control) in total lysates of G1E-ER4 cells treated for 24h or 48h with 3 μM, 10 μM or 20 μM DOT1L inhibitor (inhDOT1L: EPZ004777) or equal volume of DMSO diluent. (B) Western blot analysis of H3K79me2 and ACTIN (loading control) in total lysates of IkNull and IkWT Ter119+ cells treated with 3 μM DOT1L inhibitor or equal volume of DMSO and co-cultured with OP9 or OP9-DL1 cells for 48h. (C) ChIP assays with H3K79me2 antibodies or isotype-matched IgG conducted in IkWT or IkNull Ter119+ cells after 48h of co-culture with OP9-DL1 cells and treatment with 3 μM DOT1L inhibitor or equal volume of DMSO diluent; immunoprecipitated and unbound (input) chromatin samples were used as templates in qPCR analysis with primers specific for Cdkn1a or Tp53 TSS; y-axis: fold enrichment levels calculated according to the Pfaffl equation using the Thp1 promoter region as internal control, are represented by bars and plotted as the mean ± Standard Deviation (SD) of the measurements; a value of 1 indicates no enrichment; data shown are the results of three independent experiments; *: p ≤ 0.05 by Student’s t-test. (D) Gene expression; the relative expression levels of Cdkn1a and Tp53 genes in IkWT or IkNull Ter119+ cells after 48h of co-culture with OP9-DL1 cells and treatment with 3 μM DOT1L inhibitor or equal volume of DMSO diluent, were measured by qRT-qPCR, calculated according to the Pfaffl equation using Hprt as internal control and normalized to IkWT/OP9 DMSO-treated; y-axis: relative fold enrichment levels are represented by bars and plotted as the mean ± SD of the measurements; data shown are the results of three independent experiments; *: p ≤ 0.05 by Student’s t-test. (TIF) [file pgen.1009478.s016.tif]

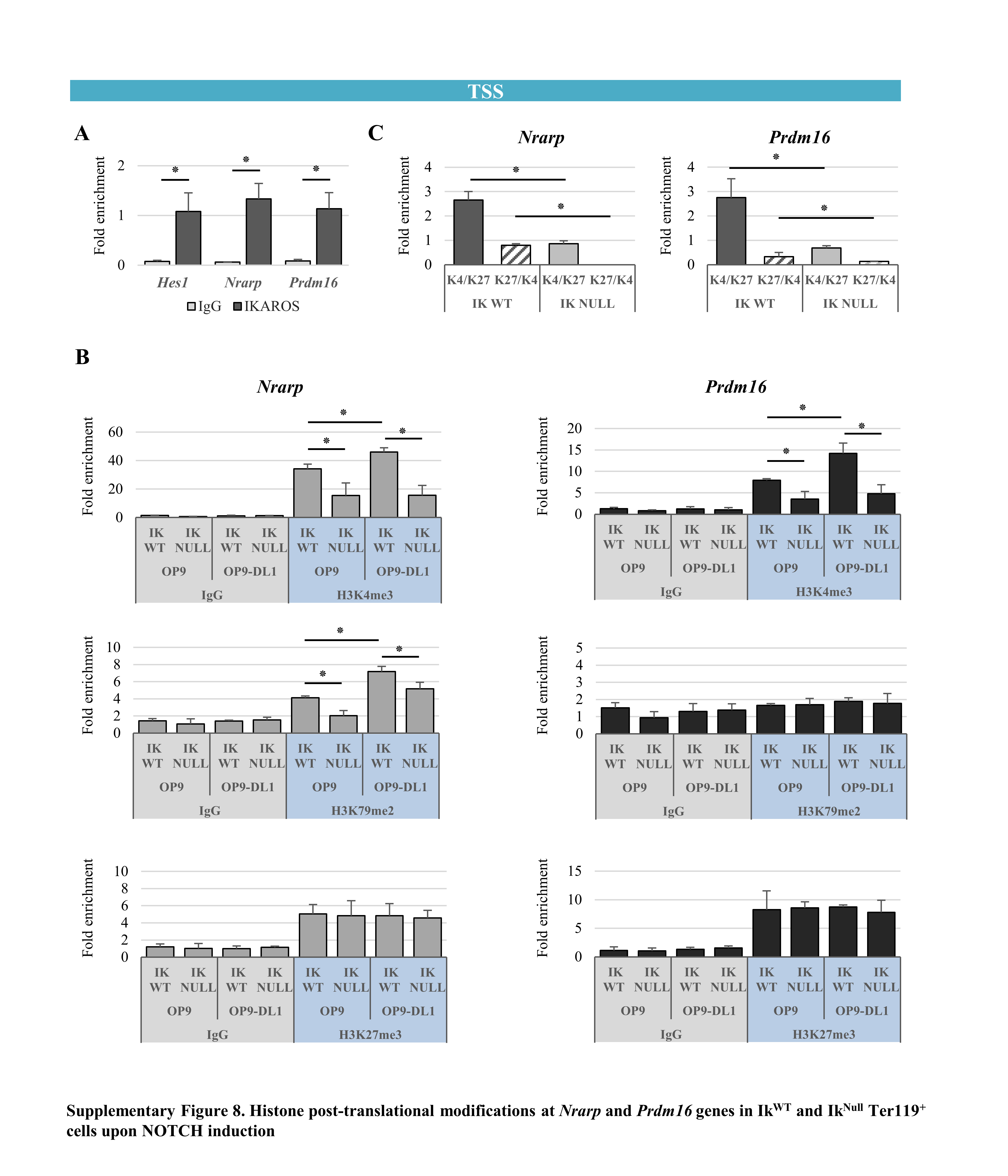

Supplement: S8 Fig — (A-B) ChIP assays with IKAROS, H3K4me3, H3K79me2, H3K27me3 antibodies (blue) or isotype-matched IgG (grey) were conducted in IkWT or IkNull fetal liver erythroid cells (panel A) or IkWT or IkNull Ter119+ cells co-cultured with OP9 or OP9-DL1 cells (panel B); immunoprecipitated and unbound (input) chromatin samples were used as templates in qPCR analysis with primers specific for Nrarp, Prdm16 TSS, Hes1 promoter (IKAROS positive control in panel A); the minor satellite region was the negative control for IKAROS ChIP assay (presented in Fig 3E), and for the H3K4me3, H3K79me2, H3K27me3 ChIP assays (presented in S6A Fig); y-axis: fold enrichment levels calculated according to the Pfaffl equation using the Thp1 promoter region as internal control, are represented by bars and plotted as the mean ± Standard Deviation of the measurements; a value of 1 indicates no enrichment; data shown are the results of three independent experiments; *: p ≤ 0.05 by Student’s t-test. (C) Sequential ChIP (re-ChIP) assays carried out on erythroid cells isolated from e14.5 IkWT or IkNull fetal livers with antibodies against H3K4me3 or H3K27me3. K4/K27: H3K4me3 antibodies were used for the first round of precipitation and H3K27me3 antibodies for the second ChIP; K27/K4: H3K27me3 antibodies were used for the first round of precipitation and H3K4me3 antibodies for the second ChIP. Immunoprecipitated and unbound (input) chromatin samples were used as templates in qPCR analysis with primers specific for Prdm16 or Nrarp TSS; y-axis: fold enrichment levels calculated according to the “percentage input method” (https://www.thermofisher.com/ca/en/home/life-science/epigenetics-noncoding-rnaresearch/chromatin-remodeling/chromatin-immunoprecipitation-chip/chip-analysis.html) are represented by bars and plotted as the mean ± Standard Deviation of the measurements; in the re-ChIP samples, no amplification for Thp1 promoter region (used as internal control) could be observed; as well, no amplification for P [file pgen.1009478.s017.tif]

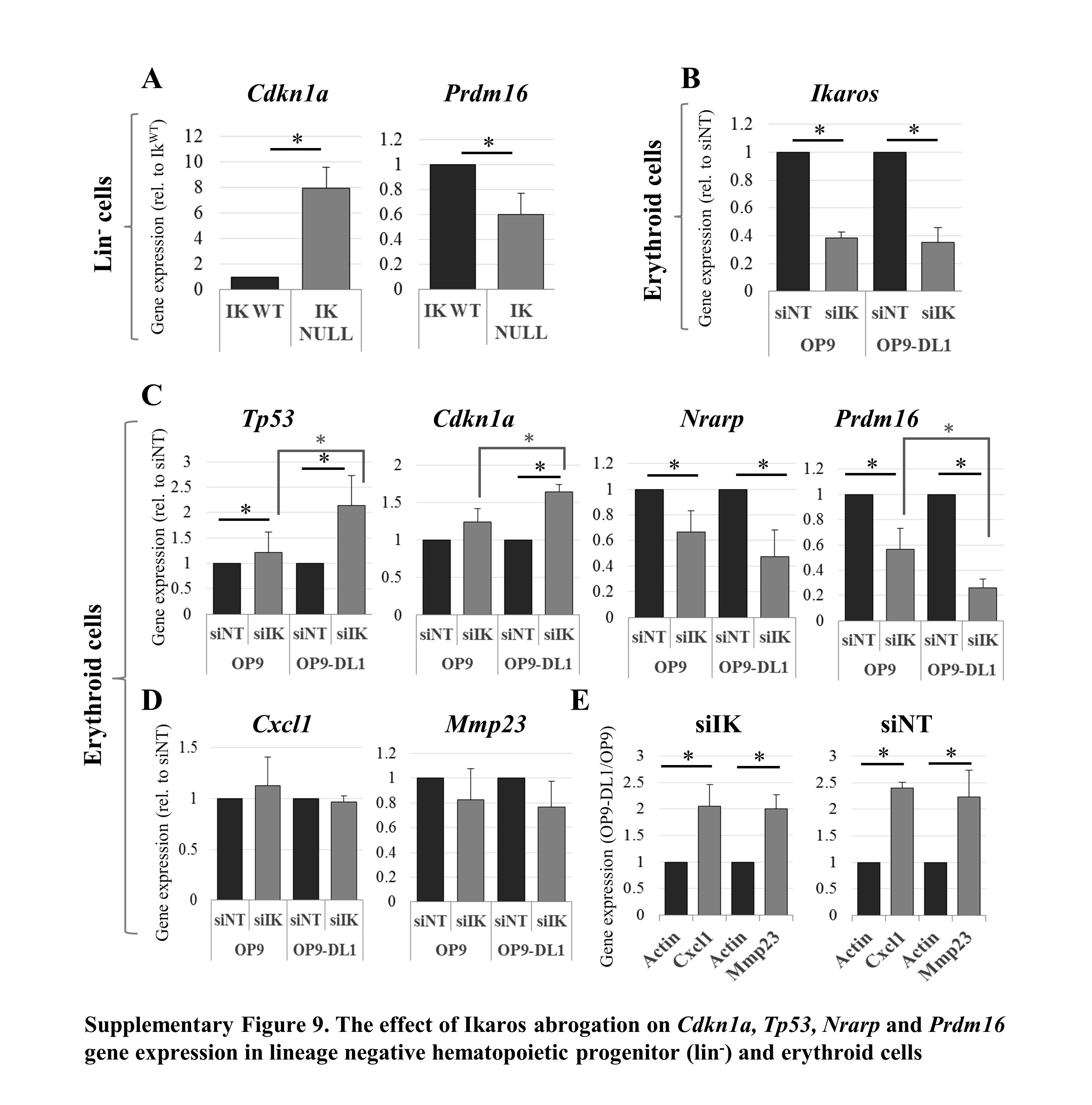

Supplement: S9 Fig — The relative expression level of Cdkn1a, Tp53, Nrarp, Prdm16, Cxcl1 and Mmp23 genes in IkNull lin- (A) as well as in Ikaros knock-down (siIK) erythroid cells (B-E) was measured by qRT-PCR, calculated according to the Pfaffl equation using Actin as internal control. (A) Ikaros target gene expression values in IkNull lin- cells were normalized to the values obtained in IkWT lin- cells. (B, C, D) Ikaros (B) or target gene (C, D) expression values in siIK-transfected erythroid cells (IkKD in the main text) were normalized to the values obtained in siNT (non-target siRNA)-transfected erythroid cells; (E) expression values in siIK-transfected (left panel) or siNT-transfected (right panel) erythroid cells co-cultured with OP9 or OP9-DL1 cells; gene expression values in siIK/OP9-DL1 or siNT/OP9-DL1 were normalized to the values obtained in siIK/OP9 or siNT/OP9 cells respectively; y axis: relative RNA enrichment levels, ratios are represented by bars and are plotted as the mean ± Standard Deviation (SD) of the measurements; data shown are the results of three independent experiments; *: p ≤ 0.05 by Student’s t-test. (TIF) [file pgen.1009478.s018.tif]
